# Alamandine attenuates ovariectomy-induced osteoporosis by promoting osteogenic differentiation via AMPK/eNOS axis

**DOI:** 10.1186/s12891-023-07159-2

**Published:** 2024-01-10

**Authors:** Wanxin Luo, Chen Yao, Jie Sun, Bo Zhang, Hao Chen, Jin Miao, Yafeng Zhang

**Affiliations:** 1 Department of Orthopaedics, Affiliated Hospital of Nantong University, 20 Xisi Road, Nantong City, 226001 Jiangsu Province PR China; 2 Laboratory Animal Center of Nantong University, Medical School of Nantong University, Nantong City, 226001 Jiangsu Province PR China

**Keywords:** Alamandine, Ovariectomy-induced osteoporosis, Renin angiotensin system, MrgD, AMPK/eNOS axis

## Abstract

**Background:**

Alamandine is a newly characterized peptide of renin angiotensin system. Our study aims to investigate the osteo-preservative effects of alamandine, explore underlying mechanism and bring a potential preventive strategy for postmenopausal osteoporosis in the future.

**Methods:**

An ovariectomy (OVX)-induced rat osteoporosis model was established for in vivo experiments. Micro-computed tomography and three-point bending test were used to evaluate bone strength. Histological femur slices were processed for immunohistochemistry (IHC). Bone turnover markers and nitric oxide (NO) concentrations in serum were determined with enzyme-linked immunosorbent assay (ELISA). The mouse embryo osteoblast precursor (MC3T3-E1) cells were used for in vitro experiments. The cell viability was analysed with a Cell Counting Kit‑8. We performed Alizarin Red S staining and alkaline phosphatase (ALP) activity assay to observe the differentiation status of osteoblasts. Western blotting was adopted to detect the expression of osteogenesis related proteins and AMP-activated protein kinase/endothelial nitric oxide synthase (AMPK/eNOS) in osteoblasts. DAF-FM diacetate was used for semi-quantitation of intracellular NO.

**Results:**

In OVX rats, alamandine alleviated osteoporosis and maintained bone strength. The IHC showed alamandine increased osteocalcin and collagen type I α1 (COL1A1) expression. The ELISA revealed alamandine decreased bone turnover markers and restored NO level in serum. In MC3T3-E1 cells, alamandine promoted osteogenic differentiation. Western blotting demonstrated that alamandine upregulated the expression of osteopontin, Runt-related transcription factor 2 and COL1A1. The intracellular NO was also raised by alamandine. Additionally, the activation of AMPK/eNOS axis mediated the effects of alamandine on MC3T3-E1 cells and bone tissue. PD123319 and dorsomorphin could repress the regulating effect of alamandine on bone metabolism.

**Conclusion:**

Alamandine attenuates ovariectomy-induced osteoporosis by promoting osteogenic differentiation via AMPK/eNOS axis.

**Supplementary Information:**

The online version contains supplementary material available at 10.1186/s12891-023-07159-2.

## Introduction

Osteoporosis is a systemic disorder of bone metabolism characterized by deterioration of bone microarchitecture, continuously decreased bone mass, increased bone fragility and elevated fracture risk [1]. Postmenopausal osteoporosis remains the most common type of osteoporosis that leads to fracture in millions of menopausal women worldwide [2]. Although estrogen deficiency is a major cause of postmenopausal osteoporosis, estrogen replacement therapy is not commonly recommended for osteoporosis treatment because of the potential risk of cardiovascular events and cancers of the breast and uterus [3–6].

The renin angiotensin system (RAS) is an elaborate endocrine system that has powerful effects on blood pressure and sodium homeostasis [7]. Angiotensin II (AngII), as the key member of classical RAS, plays a vital role in various biological actions [8]. Aberrant activation of AngII is related to the progression of the cardiovascular, renal, and liver diseases [9]. Notably, AngII was also found to inhibit osteogenic differentiation, provoke osteoclastic activity and accelerate osteoporosis in ovariectomized rats [10]. ACE inhibitors (ACEIs) and angiotensin type 1 receptor blockers (ARBs) may help maintain bone mass in ovariectomized rat [10–12]. Patients who were treated with ACEI had lower risks of bone loss and fragility fractures [13]. These studies suggested that local RAS was involved in bone metabolism and AngII may be a promising therapeutic target for postmenopausal osteoporosis.

Alamandine is a new peptide of the non-canonical RAS characterized in 2013 which is generated by hydrolysis of Angiotensin A or decarboxylation of Angiotensin-(1 − 7) [14]. Alamandine and its receptor, Mas-related G protein-coupled receptor member D (MrgD) are now considered novel members of the RAS protective arm because of its antagonistic effect against AngII [15, 16]. It has been proved that alamandine has similary functions with Angiotensin-(1 − 7) such as vasodilation, anti-inflammatory and anti-fibrosis [14, 17]. Additionally, alamandine was reported to have the function of inducing AMPK activation and nitric oxide (NO) production in cardiomyocytes [14, 18]. Interestingly, NO and AMPK were also documented to be associated with bone metabolism [19]. However, the role of alamandine on bone metabolism and postmenopausal osteoporosis remains unclear.

In this study, the potential effects of alamandine on postmenopausal osteoporosis were investigated in OVX-induced osteoporosis rat models and the underlying mechanisms were explored using in vitro cell models.

## Methods

### Inclusion complex and drug preparation

The inclusion compound HP-β-CD/alamandine was prepared for in vivo experiments as previously described [14]. (2-Hydroxypropyl)-β-Cyclodextrin (HP-β-CD) was purchased from Sigma-Aldrich (332,607, average Mw ~ 1,460, USA). Inclusion complex between the alamandine (TGpeptide, Nanjing, China) and the HP-β-CD was prepared by the freeze-drying process using the 1:1 molar ratio. An aqueous solution, using Milli-Q® water, of host and guest molecules was stirred for 3 h to ensure that equilibrium had been reached. Then, the solution was frozen in liquid nitrogen and lyophilized (LC-18 N-50 A Freeze-Dryer, LICHEN, China) for 48 h to obtain the solid inclusion complex and stored at -20℃ for later use. PD123319, the MrgD antagonist, was purchased from Abcam and dissolved in distilled water for oral gavage.

### Animals, groups, treatment and sampling

**Fig. 1:**
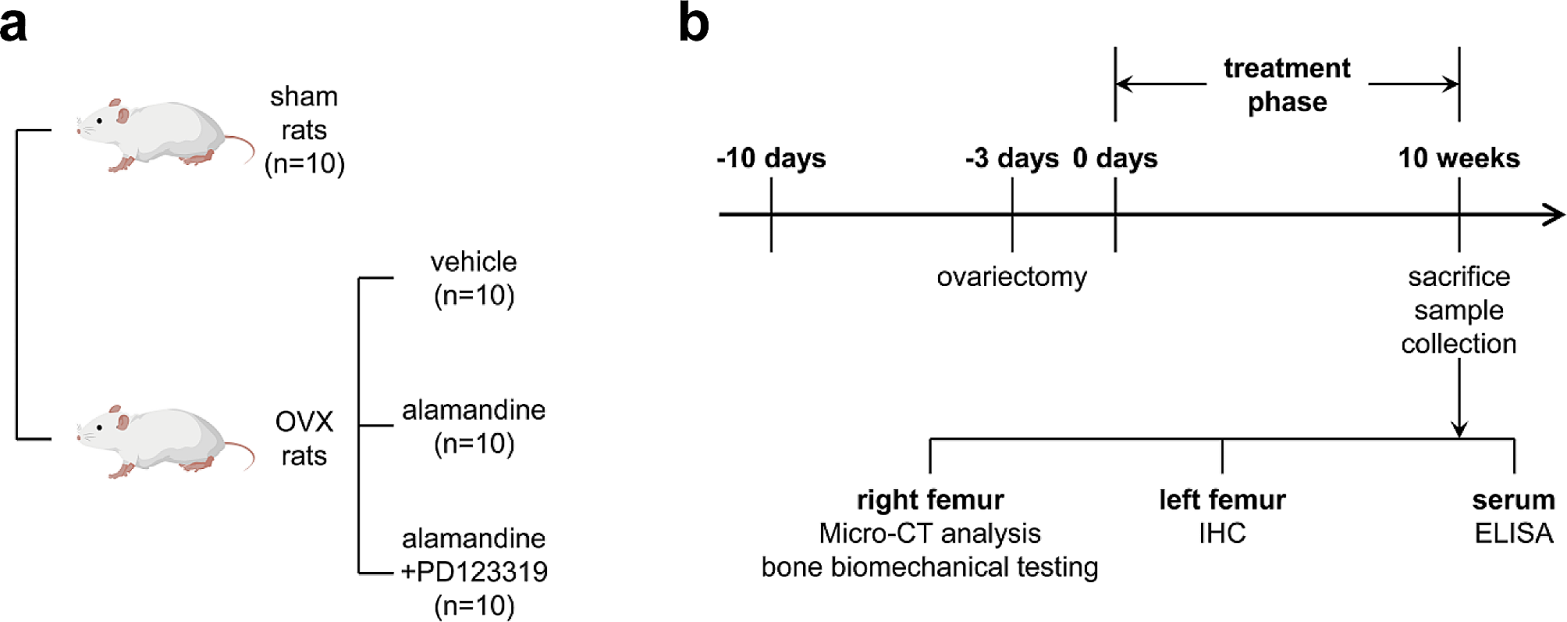
Animal protocol. Experimental grouping: Sham group, OVX group, OVX + alamandine group and OVX + alamandine + PD123319 group (**a**). Experimental timeline and the schematic diagram of tissue collection and detection (**b**)

Forty 8-week-old female Sprague-Dawley rats, weight 200-220 g, were purchased from Laboratory Animal Center of Nantong University, housed under the specific pathogen-free (SPF) conditions, and maintained under standard laboratory conditions (temperature, 25 ± 2℃; humidity, 50 ± 5%), with a 12 h:12 h light/dark cycle. All rats were received standard rat chow and water *ad libitum*. Overall animal experimental designs and schemes were approved by Institutional Animal Care and Use Committee (IACUC) of Medical School, Nantong University (No. IACUC20220113-1002). After one week of adaptive feeding, a bilateral OVX (thirty rats) or sham operation (ten rats) was performed using standard methods. Briefly, all rats were weighed and reciprocal ovariectomy was done under general anaesthesia (60 mg/kg ketamine and 10 mg/kg xylazine). Ovaries were extracted through a mid-abdominal incision. For the sham group, only a mid‐abdominal incision was made. After surgery, animals were treated with Benzylpenicillin Sodium (300,000 UI/kg, ip) and Flunixin Meglumine (2.5 mg/kg, sc) for 3 days. Thirty OVX animals were randomly divided into three experimental groups: OVX group, OVX + alamandine group and OVX + alamandine + PD123319 group, each group has ten rats. The OVX model rats received treatment once per day by oral gavage (using reusable oral gavage needles) of HP-β-CD (vehicle, 84 µg/Kg/day), alamandine included in HP-β-CD (134 µg/Kg/day, equivalent to 50 µg/Kg/day of alamandine) or PD123319 (0.3 mg/Kg/day) mixed with alamandine inclusion compounds, respectively. All gavage drugs were dissolved in distilled water before treatment. After a 10-week treatment period, the rats were sacrificed for analysis. After euthanasia by overdose of ketamine, the blood sample was collected from heart for serum isolation and then stored at -80℃ before use. The right side femur was harvested for measurement of bone mineral density (BMD) and bone micro-architecture by micro-computed tomography (Micro-CT) prior to mechanical testing and left side femur samples were harvested for histological and immunohistochemical evaluation. The animal protocol is shown in Fig. 1.

### Micro-computed tomography scanning and analysis

Micro-CT (SkyScan-1276 micro-computed tomography system, Bruker, Kontich, Belgium) was used to verify the osteoporotic condition of the bones. The right femurs were harvested and stored in -80 °C before CT scanning. Samples were scanned at 15 μm pixel resolution (1 mm aluminium filter, 85 kV, 200 µA). A total of 135 images were obtained from the distal region of each femur. The femur in Sham and OVX rats was visualized according to the sagittal and transverse planes by SkyScan CTVox software (Bruker, Karlsruhe, Germany). The analysis of the femur morphological parameter was evaluated by CTAn software (Bruker, Karlsruhe, Germany), including percent bone volume (BV/TV), bone mineral density (BMD), structure model index (SMI), trabecular number (Tb.N), trabecular thickness (Tb.Th) and trabecular separation (Tb.Sp). According to previous research, compared with cortical bone, the change of trabecular bone may reflect the degree or progress of osteoporosis more visually or directly. Therefore, we referred to some other studies and analysed the trabecular bone to evaluate the progress of bone loss in rat experiments [20–22]. SMI is used to characterize the degree of plate-like and rod-like bone trabeculae, the SMI value for absolute plate-like bone trabeculae is 0, and the SMI value for absolute rod-like bone trabeculae is 3. When osteoporosis occurs, the SMI value increases, the rod-like bone trabeculae increase, and the plate-like bone trabeculae decrease.

### Bone biomechanical testing

After micro-CT scan, the right side femur was tested for bone strength by three-point bending test [23]. The biomechanical testing was done using ElectroPuls E10000 Linear-Torsion all-electric dynamic test instrument (INSTRON, MA, USA). The right femur was positioned horizontally with the anterior surface downwards, and the span of the two support points below the bone was 20 mm. A displacement rate of 3 mm/min was selected for applying the loading vertically to mid-shaft for femurs. Load deformation curves were transferred to a personal computer and acquired by Team 490 software (version 4.10, Nicolet Instrument Technologies, WI, USA). Sigma Plot 7.0 software (Systat Software Inc, CA, USA) was used to smooth the load deformation curve and calculate the extrinsic material properties of the bone samples, including the ultimate load (N), ultimate displacement (mm), stiffness (N/mm), energy to failure (N•mm), bending stress (Mpa) and bending strain (a.u.).

### Bone turnover markers measurement

The serum levels of osteocalcin (OCN), cross-linked carboxy-terminal telopeptide of type 1 collagen (CTX-I) and nitric oxide (NO) were measured using commercially available ELISA kits according to the manufacturer’s instructions (Nanjing Jiancheng Biological Engineering Research Institute, China).

### Histological and immunohistochemical evaluation

For evaluation of bone histology, the left side femur was fixed in 4% paraformaldehyde for 48 h and then decalcified in 10% ethylenediaminetetraacetic acid for 3 weeks before being dehydrated with gradient alcohols and embedded in paraffin, and 5 μm thick contiguous sections were sliced for immunohistochemistry analysis. For IHC staining, slides were processed by heat-induced epitope retrieval using microwave oven heating in 0.01 M citrate buffer for 10 min. The sections were incubated in diluted normal serum for an hour and then incubated with primary antibodies, including antibodies against OCN (1:50, AB10911, Sigma-Aldrich, USA), COL1A1 (1:200, 72026T, Cell Signaling Technology, USA), pho-eNOS (Ser1177, 1:500, PA5-35879, Thermo Fisher, USA) and pho-AMPKα (1:200, 2535T, Cell Signaling Technology, USA) at 4 °C for 24 h. After that, the sections were rinsed with PBS three times and incubated with a biotinylated secondary antibody (dilution 1:150) for 1 h. Immunoreactivity was visualized by a solution of 0.01% H_2_O_2_ and 0.05% diaminobenzidine that generated a brown colour. Nuclei were presented with haematoxylin staining. All slides were observed by slice digital scanning (Pannoramic MIDI, 3DHISTECH, Hungary).

### Cell models and intervention

The mouse embryo osteoblast precursor (MC3T3-E1, subclone 14) cell line was obtained from the National Collection of Authenticated Cell Cultures (Shanghai, China) and routinely maintained in α-modified Eagle’s Minimum Essential Medium (α-MEM) complete medium supplemented with 10% (v/v) fetal bovine serum (Gibco, Life Technologies, Grand Island, NY, USA) and penicillin/streptomycin (pen/strep, 100 U/mL and 100 µg/mL; Gibco, USA) at 37 °C in 5% CO_2_. Thereafter, 50 µg/ml L-ascorbic acid (Sigma-Aldrich, USA), 10 mM β-glycerophosphate (Sigma-Aldrich, USA) and 10 nM dexamethasone (Sigma-Aldrich, USA) were added into α-MEM to induce osteogenic differentiation. Cell viability was estimated using the Cell Counting Kit-8 (CCK-8) assay (HY-K0301, MedChemExpress, USA). Cells were seeded into 96-well plates (5,000 cells/well) with diverse interventions, and the blank did not contain cells. At 24 and 48 h, the cells in each well were treated with 10 µL CCK-8 solution and incubated at 37 °C for 1 h. Cell viability was calculated by measuring the absorbance at 450 nm. Afterwards, the cells were counted and plated onto 6-well culture plates at a density of 1 × 10^5^ cells/well. Referring to CCK-8 results and previous studies [14, 24–26], 24 h after plating the osteoblasts, alamandine (100 nM; TGpeptide, Nanjing, China), PD123319 (1 µM, 30 min before alamandine; ab144564, Abcam, USA) and AMPK inhibitor compound C (dorsomorphin, 100 nM, 30 min before alamandine; HY-13,418 A, MedChemExpress, USA) were added. The culture medium was changed every 2 days. The groups used were: (1) Control, which received only medium; (2) alamandine (Ala); (3) alamandine + PD123319 (Ala + PD); (4) alamandine + compound C (Ala + CC).

### Alkaline phosphatase and alizarin Red S staining for mineralization

MC3T3-E1 cells were seeded in 6-well plates and cultured in osteogenic induction medium with stimulation for 14 days. Alkaline phosphatase (ALP) staining was performed with a BCIP/NBT (nitro-blue tetrazolium/5-bromo-4-chloro-3-indolylphosphate) alkaline phosphatase colour development kit (C3206, Beyotime, China) according to the manufacturer’s instructions. When the cells were induced for 28 days, Alizarin Red S (ARS) staining was performed to detect calcium deposits with modified Alizarin Red S stain kit for calcium (G3280, Solarbio, China). Stained plates were photographed using a digital camera. Images of stained cells were captured under a light microscope (BX41, Olympus, Japan) and five randomly selected fields (× 10 magnification) were photographed in each well, analysed by Image J software (version 1.53n, National Institutes of Health, USA) according to previous protocol [27–29].

### Western blotting

MC3T3-E1 cells were treated with various interventions in osteogenic medium for 48 h. After that, cells were lysed in lysis buffer (P0013, Beyotime, China) at 4 °C with protease and phosphatase inhibitors (P1045, Beyotime, China). The lysis mixture was centrifuged at 12,000 × rpm for 20 min at 4 °C, and the supernatant containing cellular proteins was used in following experiments. The protein concentration was measured by the Bicinchoninic Acid Protein Assay Kit (Bio-Rad, Hercules, CA, USA) according to the manufacturer’s protocol. Equal amounts (100 µg) of protein were denatured, separated on 8 − 12% sodium dodecyl sulphate–polyacrylamide gel electrophoresis (SDS-PAGE) and transferred to polyvinylidene difluoride (PVDF) membranes (Millipore, Bedford, MA, USA). The membranes were blocked with 5% BSA for 2 h at room temperature, then incubated with primary antibodies at 4ºC overnight. Next, membranes were incubated with horseradish peroxidase-conjugated secondary antibodies (Cell Signaling Technology, USA) for 1.5 h at room temperature. The primary antibodies used were MrgD (1:1000, ab155099, Abcam, USA), Runt-related transcription factor 2 (RUNX2, 1:1000, 12,556 S, Cell Signaling Technology, USA), Osteopontin (OPN, 1:1,000, 22952-1-AP, Proteintech, China), collagen type I α1 (COL1A1, 1:1000, 72026T, Cell Signaling Technology, USA), eNOS (1:1,000, 27120-1-AP, Proteintech, China), pho-eNOS (Ser1177, 1:1000, PA5-35879, Thermo Fisher, USA), phospho-adenosine 5‘-monophosphate-activated protein kinase α (Thr172, pho-AMPKα, 1:1000, 2535T, Cell Signaling Technology, USA), AMPKα (1:1000, 5831T, Cell Signaling Technology, USA), and GAPDH antibody (1:5000, 60004-1-Ig, Proteintech, China) used as loading control. The signals were visualized with ECL detection reagent (P0018FM, Beyotime, China) and semi-quantified with the Image J software (version 1.53n, National Institutes of Health, USA).

### Intracellular nitric oxide measurements

NO levels were measured using the NO indicator 3-Amino,4-aminomethyl-2’,7’-difluorescein, diacetate (DAF-FM DA, 5 µM, Beyotime, China) according to manufacturer’s directions. The cells were stimulated in osteogenic medium for 48 h and washed with PBS twice. Then, DAF-FM DA were added and incubated for 30 min at 37 °C. Changes in NO generation were visualized using a Leica fluorescence microscope (Leica THUNDER Imager, Wetzlar, Germany) and images were analysed by Image J software (version 1.53n, National Institutes of Health, USA).

### Statistical analyses

Statistical analyses were performed with IBM SPSS 15.0 statistics software (IBM Corp., Armonk, NY, USA), and data were presented as mean ± standard deviation (SD). All figures were drawn using GraphPad Prism 9.0 software (GraphPad Software, La Jolla, CA, USA). Statistical significance among multiple groups was evaluated by one-way analysis of variance (ANOVA) and Tukey post-hoc test. Data of CCK-8 was evaluated by two-way ANOVA. The statistical analyses were two-side and a p-value of less than 0.05 was regarded as statistically significant.

## Results

### Alamandine alleviated osteoporosis and maintained bone strength in OVX rats

**Fig. 2:**
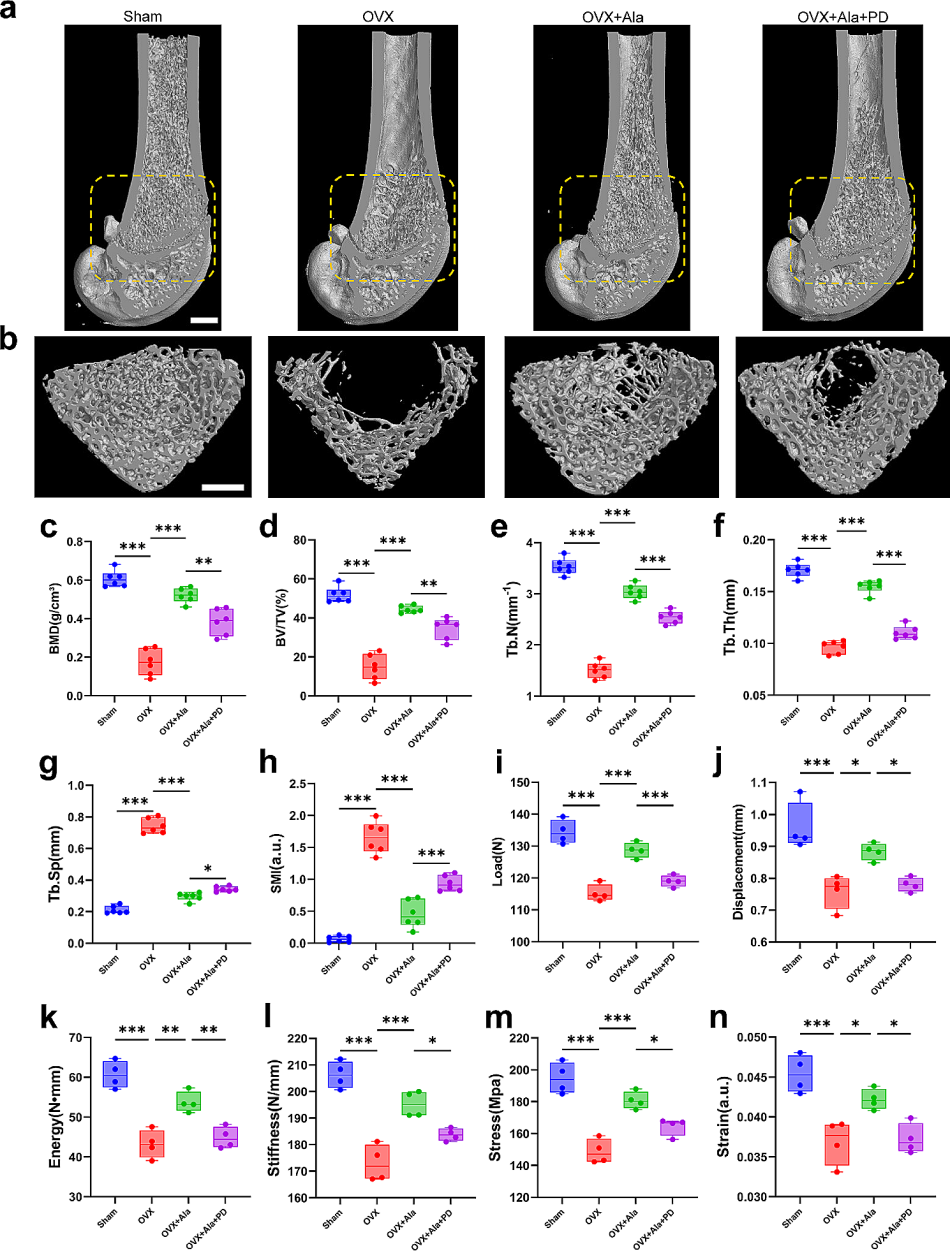
Representative Micro-CT three-dimensional images of trabecular bone microarchitecture with quantitative results of Micro-CT and three-point bending test. Sagittal and transverse planes of the femur were visualized. A region of interest (ROI) with 1.5 mm height was chosen starting 0.4 mm from the lowest end of the growth plate to the proximal end of the femur. Three-dimensional images of the right side distal femurs (**a**) and the trabecular bone microarchitecture (**b**). The scale bars represent 1 mm. Quantitative results of Micro-CT analysis expressed as BMD (**c**), BV/TV (**d**), Tb.N (**e)**, Tb.Th (**f**), Tb.Sp (**g**) and SMI (**h**). Sample size n = 6 specimens/group. Quantitative results of the mechanical properties (**i**–**n**). The histograms are ultimate load (**i**), ultimate displacement (**j**), energy to failure (**k**), stiffness (**l**), bending stress (**m**) and bending strain (**n**) of the femur diaphysis (cortical bone). Sample size n = 4 specimens/group. ***: *P* < 0.001, **: *P* < 0.01, *: *P* < 0.05

In micro-CT scanning, the sagittal and transverse slices clearly depicted the qualitative effects of different treatment on the trabecular bone of distal femur (Fig. 2a and b). After treatment with alamandine for 10 weeks, the microarchitecture parameters BMD, BV/TV, Tb.N and Tb.Th were significantly higher compared with the OVX group (Fig. 2c and f). The other parameters such as Tb.Sp, SMI were significantly lower than the OVX group (Fig. 2g and h). Furthermore, the three-point bending test showed that alamandine significantly increased the ultimate load, ultimate displacement, energy to failure, stiffness, bending stress and bending strain of the femur diaphysis compared with the OVX group (Fig. 2i and n). Meanwhile, the MrgD antagonist PD123319 blocked the effects of alamandine.

### Alamandine suppressed bone loss and decreased the level of bone turnover in OVX rats

**Fig. 3:**
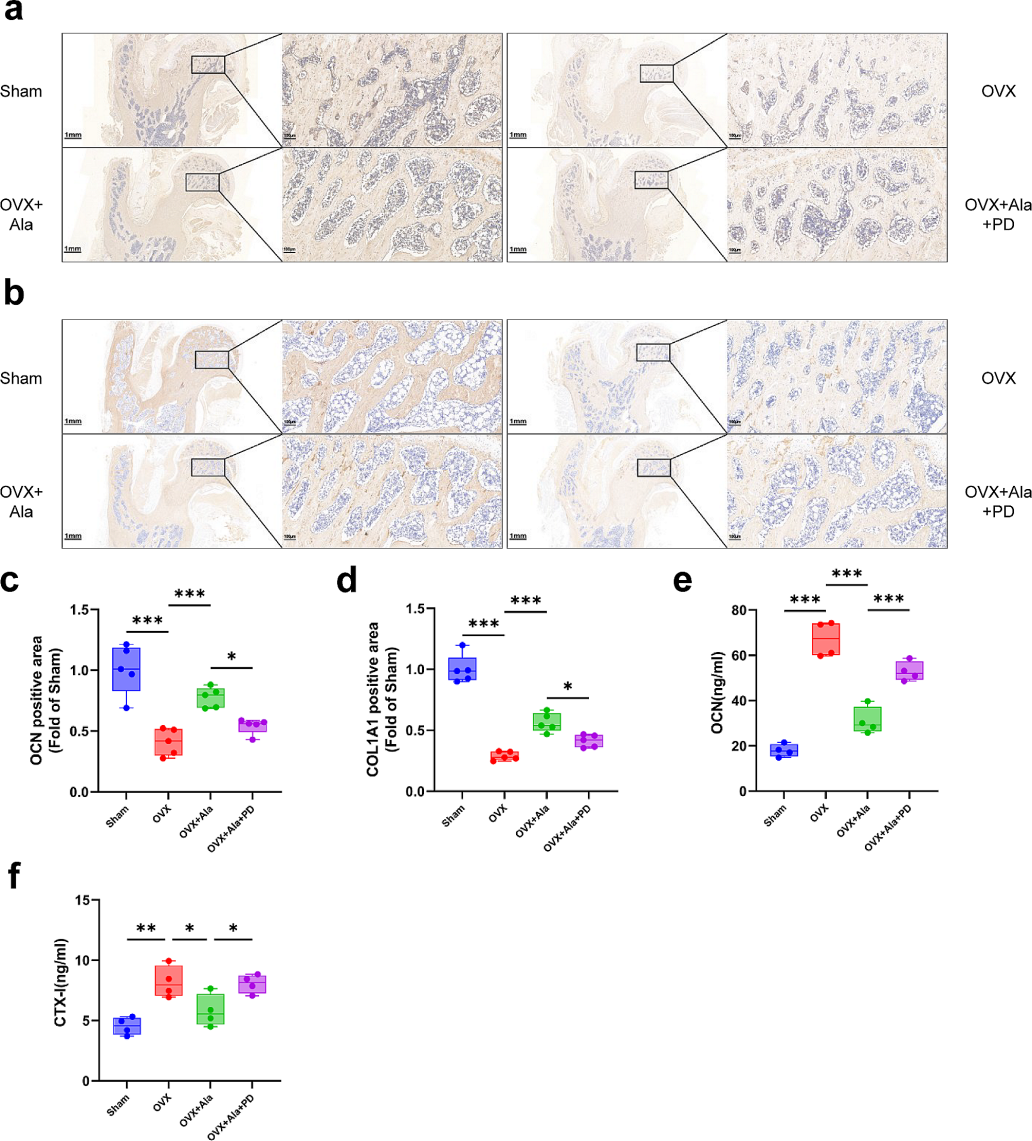
Effects of different treatment on the bone tissue and bone turnover markers of model rats. OCN (**a**) and COL1A1 (**b**) expression were detected with the sections of proximal femur (femoral head). The magnifications are × 1.5 (left parts) and × 10 (right parts), scale bars represent 1 mm and 100 μm respectively. Positive area (%) fold of Sham was semi-quantified by Image J software (**c**, **d**). Sample size n = 5 specimens /group. Serum concentrations of OCN (**e**) and CTX-I (**f**) were detected. Sample size n = 4 specimens/group. ***: *P* < 0.001, **: *P* < 0.01, *: *P* < 0.05

The immunohistochemistry analysis showed alamandine up-regulated expression of OCN and COL1A1 in bone tissue of OVX-induced osteoporosis rats (Fig. 3a and d). The concentrations of bone turnover markers OCN and CTX-I in serum were higher in OVX group compared with sham group and alamandine reversed these tendencies (Fig. 3e and f). PD123319 also inhibited the effects produced by alamandine. These experiments demonstrated that alamandine suppressed bone loss and decreased the level of bone turnover through MrgD in OVX-induced osteoporosis rats.

### Alamandine increased the osteogenic mineralization capacity and ALP activity in osteoblasts

**Fig. 4:**
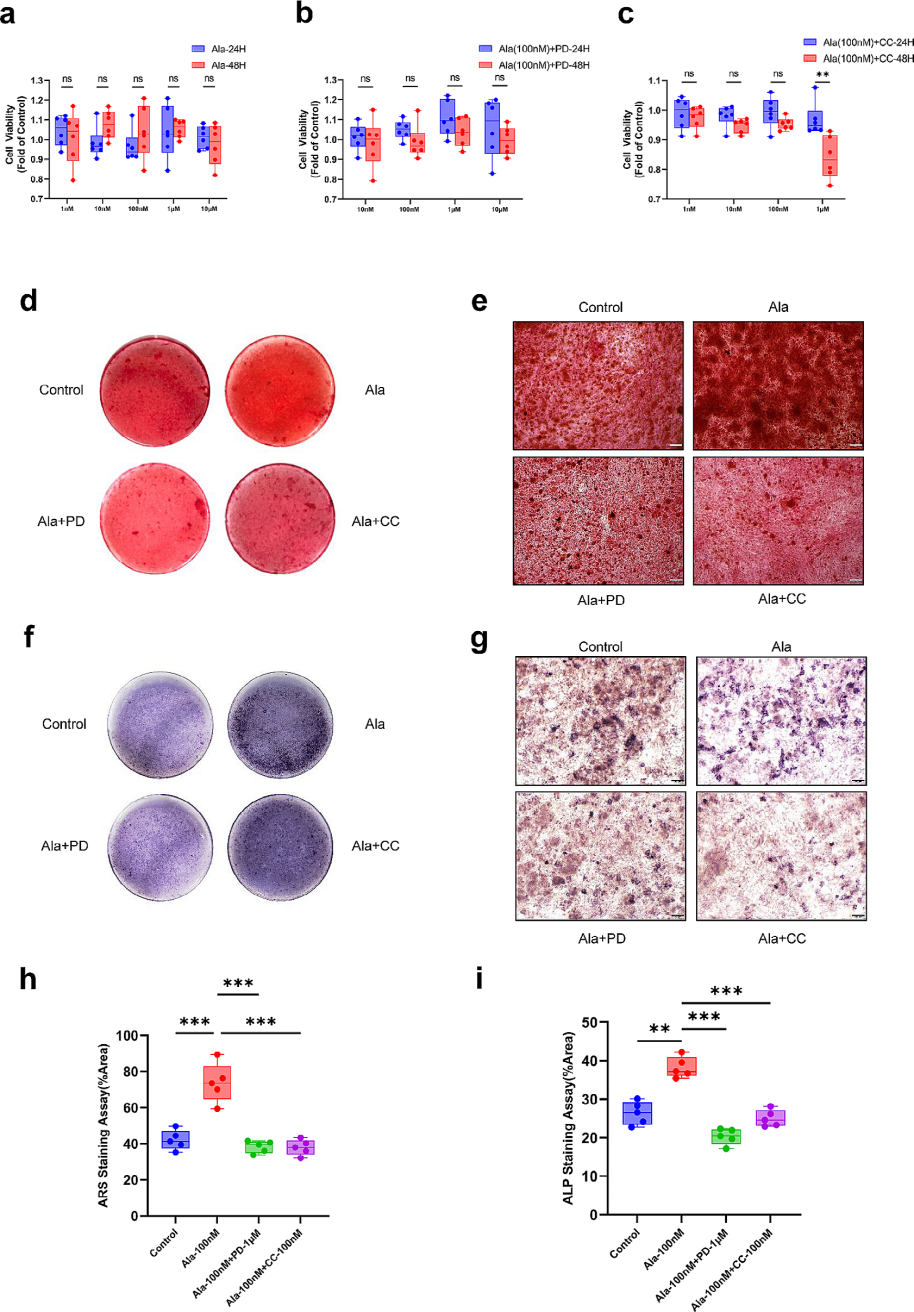
Effects of diverse intervention on cell viability and osteogenic differentiation of MC3T3-E1 cells. Cell viability under different concentrations of alamandine (**a**), PD123319 and compound C with 100 nM alamandine respectively (**b**, **c**). Sample size n = 6 wells/group. ARS staining after osteogenic induction for 21 days (**d**, **e**). ALP staining after osteogenic induction for 14 days (**f**, **g**). The magnification is × 10 and the scale bars represent 100 μm (**e**, **g**). Mineralized area (%) and ALP staining positive area (%) were used to semi-quantify the osteogenic mineralization capacity and ALP activity respectively (**h**, **i**). Sample size n = 5 images/group. ***: *P* < 0.001, **: *P* < 0.01, ns: *P* > 0.05

As shown by the CCK-8 assay, alamandine with different concentrations had no obvious effect on the cell viability of MC3T3-E1 cells in certain time (Fig. 4a). Then we chose intermediate concentration (100 nM) of alamandine to intervene cells with different concentrations of PD123319 and compound C. Similarly, alamandine with different concentrations of PD123319 did not affect the cell viability (Fig. 4b). However, a significant decline was observed in the group of alamandine with compound C (1 µM) (Fig. 4c). Therefore, the appropriate drug concentrations were selected to perform the following cell experiments (100 nM alamandine, 1 µM PD123319, 100 nM compound C). The ARS and ALP staining showed mineralized area (%) and ALP staining positive area (%) of MC3T3-E1 cells in alamandine group were significantly higher than other groups (Fig. 4d and g). Alamandine of 100 nM increased the calcium nodule formation and the ALP activity compared to control group, while PD123319 (1 µM) and compound C (100 nM) inhibited the enhancement of osteogenic mineralization capacity (Fig. 4h and i).

### Alamandine promoted the expression of osteogenesis related proteins and AMPK/eNOS in osteoblasts

**Fig. 5:**
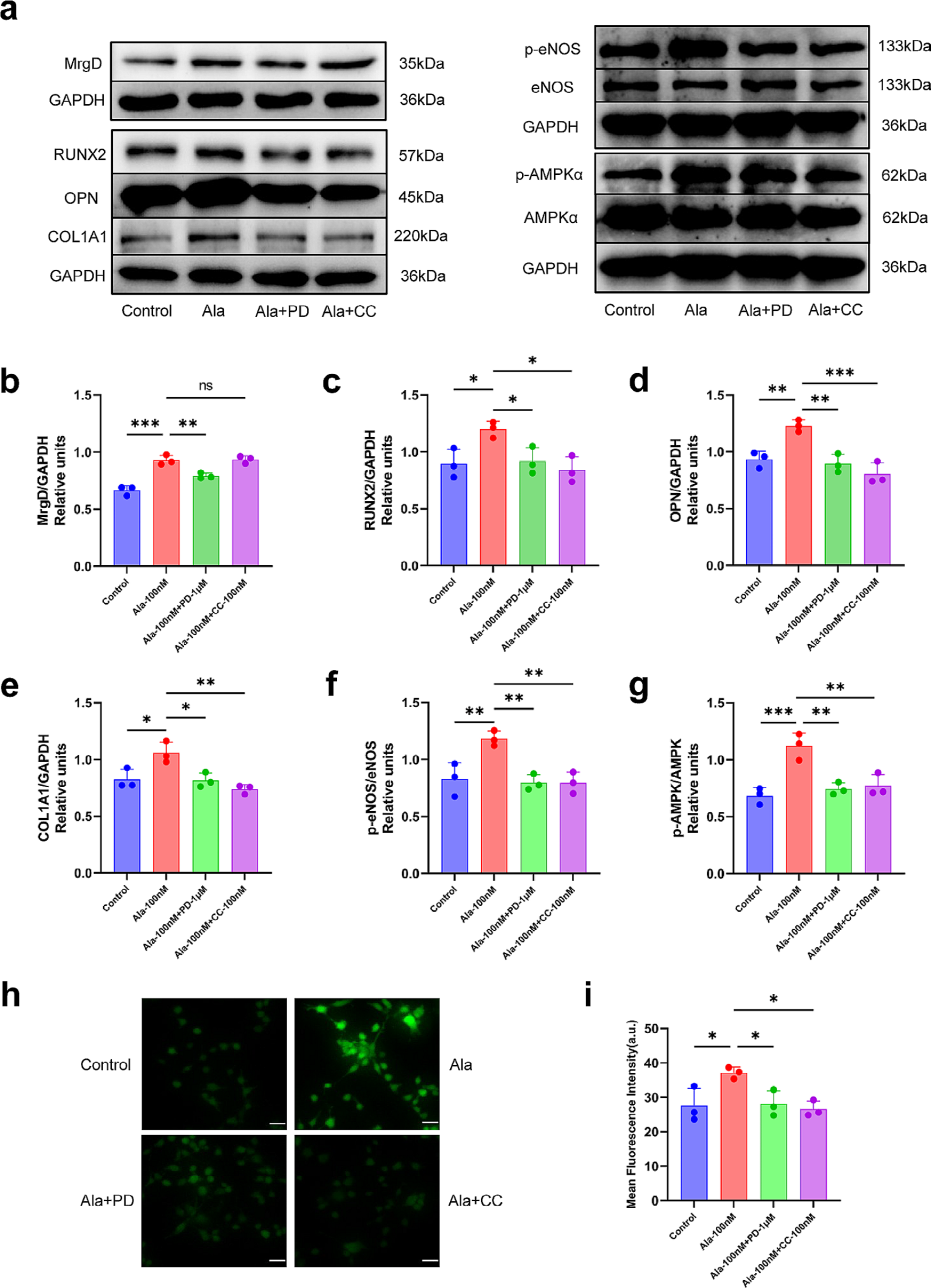
Effects of various interference on protein expression of MrgD, osteogenesis and AMPK/eNOS, and intracellular NO generation in MC3T3-E1 cells. The expression of MrgD (**a**, **b**). Representative images of osteogenic proteins (RUNX2, OPN and COL1A1) expression (**a**, **c**–**e**). The expression of pho-eNOS and eNOS (**a**, **f**). The expression of AMPKα and its phosphorylated form (**a**, **g**). The gels were cropped, the samples derive from the same experiment and the gels/blots were processed in parallel. The images of phosphorylated form and total amount of AMPKα and eNOS were obtained from the same membranes which were stripped and re-probed respectively. Semi-quantitation of intracellular NO was performed by DAF-FM diacetate (**h**, **i**). Sample size n = 3 images/group. The magnification is × 40 and the scale bars represent 10 μm. ***: *P* < 0.001, **: *P* < 0.01, *: *P* < 0.05, ns: *P* > 0.05

Western blotting showed MC3T3-E1 cells expressed MrgD, which was increased by alamandine. PD123319 inhibited MrgD, while the AMPK inhibitor compound C did not have such effect on MrgD (Fig. 5a and b). Alamandine up-regulated the expression of osteogenic proteins such as RUNX2, OPN and COL1A1 significantly compared with the control group, which were blocked by PD123319 and compound C (Fig. 5a, c and e). Alamandine promoted the expression of pho-eNOS and pho-AMPKα, which were inhibited by PD123319 and compound C (Fig. 5a, f and g). These results suggested Alamandine combined with MrgD promoted osteogenesis via AMPK/eNOS axis. After confirming the activation of eNOS, we furtherly observed that alamandine enhanced NO generation in MC3T3-E1 cells. Importantly, PD123319 and compound C attenuated alamandine-induced NO generation in MC3T3-E1 cells (Fig. 5h and i), suggesting key roles of MrgD and AMPK/eNOS axis in NO generation.

### Alamandine activated AMPK/eNOS axis and increased serum NO content in OVX rats

**Fig. 6:**
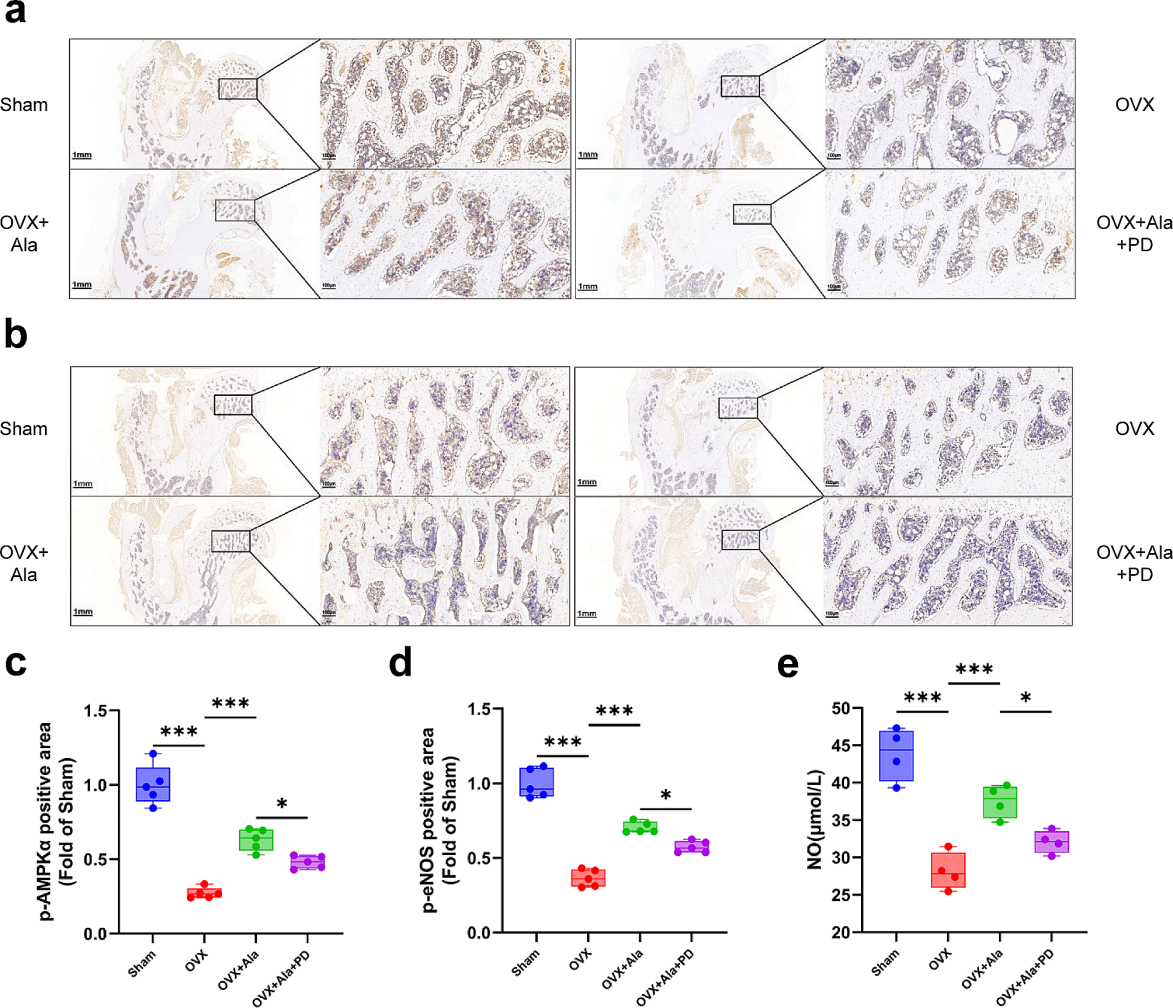
Effects of different treatment on the bone tissue and serum NO of model rats. pho-AMPKα (**a**) and pho-eNOS (**b**) expression were detected with the sections of proximal femur (femoral head). The magnifications are × 1.5 (left parts) and × 10 (right parts), scale bars represent 1 mm and 100 μm respectively. Positive area (%) fold of Sham was semi-quantified by Image J software (**c**, **d**). Sample size n = 5 specimens /group. Serum concentration of NO (**e**) was detected. Sample size n = 4 specimens/group. ***: *P* < 0.001, *: *P* < 0.05

In the bone tissue, the expression of pho-eNOS and pho-AMPKα decreased after OVX (Fig. 6a and d). Moreover, the serum NO concentration declined in OVX group (Fig. 6e). However, alamandine treatment reversed the trends all above in OVX rats and was also repressed by PD123319. Results of IHC analyses and serum NO determination validated the findings in cell experiments.

## Discussion

**Fig. 7:**
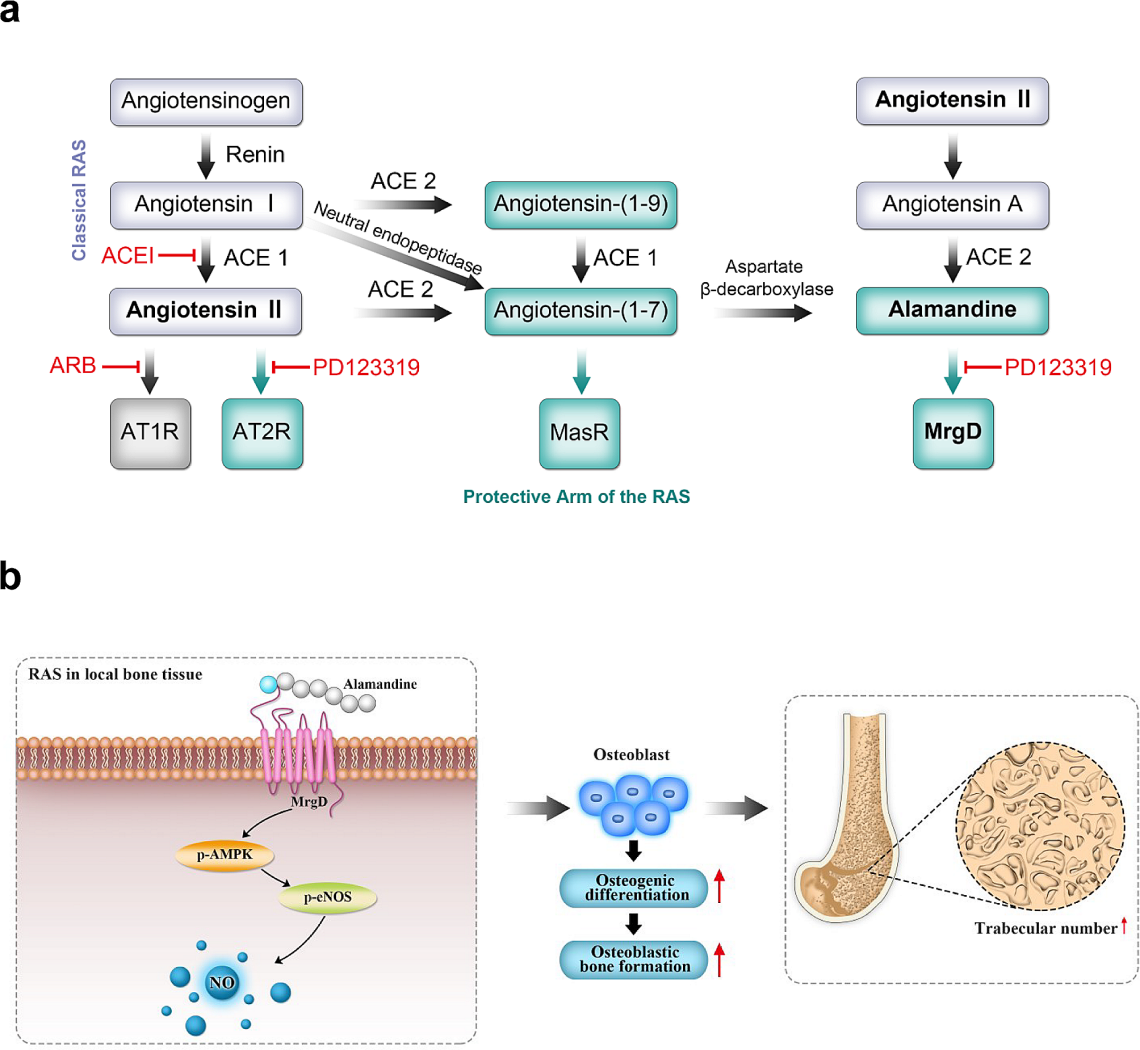
Classical vs. protective arm of the renin-angiotensin system (**a**). Alamandine combined with its receptor MrgD, attenuates OVX-induced osteoporosis by promoting osteogenic differentiation via AMPK/eNOS axis (**b**)

Numerous investigations reported that the local expression of RAS components such as renin, ACE-1, and AngII receptors in the skeletal system plays a vital role in local bone remodelling and participates in the progress of osteoporosis [10, 30, 31]. RAS inhibitors or other analogs may be a promising strategy for the therapy of osteoporosis. Alamandine is a vasoactive peptide of the non-classical RAS combined with the receptor MrgD (Fig. 7a), and it shows multiple protective effects against AngII like its precursor Angiotensin-(1 − 7) [15]. In this study, we firstly used the OVX rat models to assess the effects of oral alamandine on the estrogen-deficiency induced osteoporosis. In vivo experiments revealed that alamandine increased osteogenic markers, downregulated bone turnover markers, maintained bone strength and alleviated progress of osteoporosis in OVX rats. In in vitro experiments, we found alamandine promoted osteogenic differentiation of MC3T3-E1 cells. In terms of mechanism, we confirmed that alamandine upregulated expression of AMPK/eNOS axis and raised intracellular NO thereby. Additionally, the effects of alamandine on OVX rats or MC3T3-E1 cells above were blocked by MrgD blocker or AMPK inhibitor. In conclusion, we demonstrated that alamandine, combined with its receptor MrgD, alleviated OVX-induced bone loss by promoting osteoblast differentiation via AMPK/eNOS/NO pathway (Fig. 7b).

Postmenopausal osteoporosis is the most common form of osteoporosis associated with significant morbidity, mortality, deterioration in the quality of life and financial costs [3]. Estrogen deficiency is the main cause of postmenopausal osteoporosis. Estrogen can promote early osteoblast differentiation, stimulate collagen formation, and inhibit osteoclast activity [32]. Therefore, estrogen deficiency in postmenopausal women often causes attenuated osteogenic differentiation, increased osteoclast activity and bone turnover rate, which lead to increased bone resorption, decreased calcium salt deposition and bone mineral density [33]. Although estrogen replacement therapy can prevent bone loss to some extent, long-term estrogen treatment has various side-effects including breast tumors [4]. Accordingly, it is of great significance to explore new, safe and effective drugs for the treatment of postmenopausal osteoporosis. Our study preliminarily confirmed that alamandine could promote osteogenic differentiation, decrease bone turnover rate, and delay bone loss. In addition, it has previously been observed that alamandine has multi-system effects such as vasodilation, anti-inflammatory and anti-fibrosis [14, 34]. Moreover, no significant side-effects or adverse effects on animal health of alamandine have been found in the former research. As a physiologically existing active peptide, it has a relatively good biological safety [14], thus alamandine is expected to be a complementary treatment for postmenopausal osteoporosis at current stage.

NO is a biologically active neurotransmitter produced from L-arginine catalysed by three nitric oxide synthase (NOS) isoforms: endothelial NOS (eNOS), neuronal NOS (nNOS) and inducible NOS (iNOS) [35]. eNOS-derived NO increases osteoblastic bone formation [36] and directly inhibits osteoclast-mediated bone resorption [37]. The low level of NO can lead to enhanced cytokine induced bone resorption which is strongly associated with osteoporosis in postmenopausal estrogen deficient women [38]. Estrogen supplementation can prevent postmenopausal osteoporosis and exert a protective effect on bone tissue by promoting the release of NO from osteoblasts and osteoclasts [39]. Previous research revealed that the protective effect of estrogen on cardiovascular system is partially achieved by promoting the release of NO in vascular endothelium to dilate blood vessels [39]. We speculated that alamandine and estrogen may share many similarities in their biological effects because alamandine can also induce NO generation in the cardiovascular system, cause vasodilation and inhibiting cardiac hypertrophy [14, 24]. Alamandine has vasodilatory properties in the mouse vasculature, suggesting a signalling cascade linked to the stimulation of endothelial nitric oxide synthase (eNOS) in vascular endothelium [40]. Consequently, we supposed the relevant mechanism is also likely to play a role in regulating bone loss and conducted related experiment to verify our hypothesis.

AMP-activated protein kinase (AMPK) appears to be the main target for alamandine-induced NO formation [24]. Previous studies have shown that AMPK can regulate the differentiation and function of bone cells, and the mice with AMPKα or β subunit knockout developed a decline in bone volume [41–43]. Kanazawa et al. found the metformin promoted osteogenic differentiation by activating AMPK to increase the expression of eNOS [44]. In addition, metformin can stimulate osteogenesis in MC3T3-E1 cells, and the pro-osteogenic effect is reversed by inhibitor of AMPK, the compound C (dorsomorphin) [45]. Similar to previous research, we speculated and confirmed that alamandine binding with its receptor MrgD, can attenuate osteoporosis progression by regulating NO generation via AMPK/eNOS pathway.

Our study provided evidence that the AMPK and eNOS/NO system mediated the regulation of osteogenic differentiation by alamandine. In fact, the pathways by which alamandine produces nitric oxide are not unique [46]. PD123319, the MrgD antagonist, is also considered an angiotensin II type II receptor (AT2R) antagonist [47], although alamandine has rarely been reported as an AT2R ligand. Compound C may also inhibit the bone morphogenetic protein (BMP) signalling [48] which is associated with osteogenesis [49]. Besides the decreased osteogenesis, increased osteoclast activity is responsible for the elevated bone turnover rate in postmenopausal osteoporosis [50]. Previous studies reported that Angiotensin-(1 − 7) inhibited osteoclast differentiation [51, 52], and repressed osteoclastogenesis factors such as cathepsin K and MMP9 in RAW 264.7 cells via p38/ERK pathway [53]. These remind us that alamandine may also regulate osteoclast function, which needs to be clarified in subsequent studies. At last, we adopted the oral dose of alamandine in rats just according to methods reported previously in other diseases [14]. The most appropriate therapeutic dose of alamandine for osteoporosis is worth exploring, which have implications for subsequent studies and clinical translation.

In summary, the present study provided the evidence that alamandine stimulated the eNOS to regulate NO generation via activation of AMPK. Alamandine can attenuate the postmenopausal osteoporosis progression in OVX rats, and it may bring a potential preventive strategy for postmenopausal osteoporosis in the future.

### Electronic supplementary material

Below is the link to the electronic supplementary material.

Supplementary Material 1

Supplementary Material 2

Supplementary Material 3

Supplementary Material 4

## Data Availability

Yafeng Zhang is responsible for confirming the authenticity of the raw data. The datasets used and/or analysed during the current study are available from the corresponding author on reasonable request.

## Abbreviations

OVX: Ovariectomy
IHC: Immunohistochemistry
NO: Nitric oxide
ELISA: Enzyme-linked immunosorbent assay
ALP: Alkaline phosphatase
AMPK: AMP-activated protein kinase
eNOS: Endothelial nitric oxide synthase
COL1A1: Collagen type I α1
RAS: Renin angiotensin system
AngII: Angiotensin II
MrgD: Mas-related G protein-coupled receptor member D
BMD: Bone mineral density
BV/TV: Percent bone volume
SMI: Structure model index
Tb.N: Trabecular number
Tb.Th: Trabecular thickness
Tb.Sp: Trabecular separation
RUNX2: Runt-related transcription factor 2
OPN: Osteopontin
OCN: Osteocalcin
CTX-I: Cross-linked carboxy-terminal telopeptide of type 1 collagen
ARS: Alizarin Red S
ERK: Extracellular regulated protein kinases
