# Supplementary figures and images for: Alamandine attenuates ovariectomy-induced osteoporosis by promoting osteogenic differentiation via AMPK/eNOS axis
Source: BMC Musculoskelet Disord. 2024 Jan 10;25:45. doi: 10.1186/s12891-023-07159-2 (PMC10777585; doi:10.1186/s12891-023-07159-2)

## ARS Staining: ( $\times 10$ )

### ① PBS

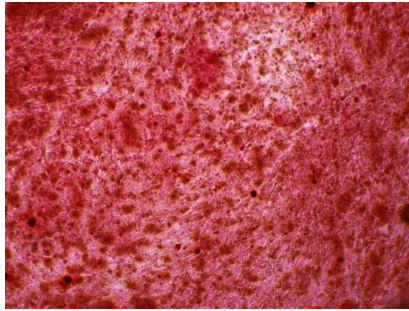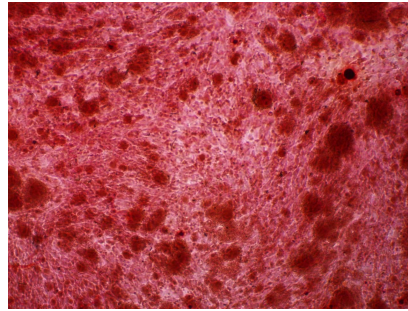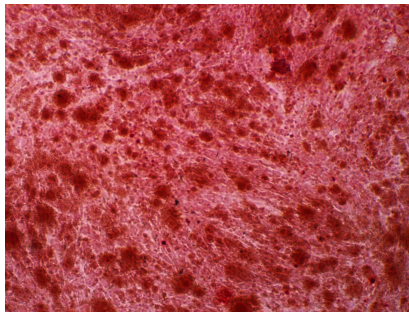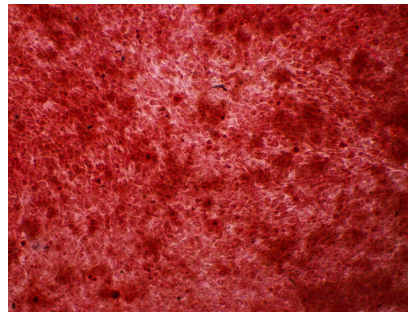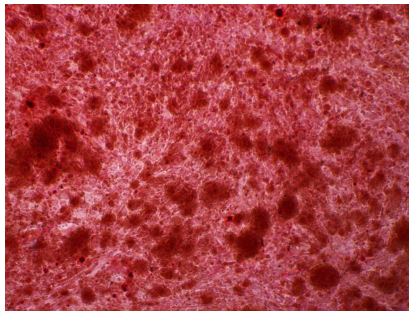

## ② Ala

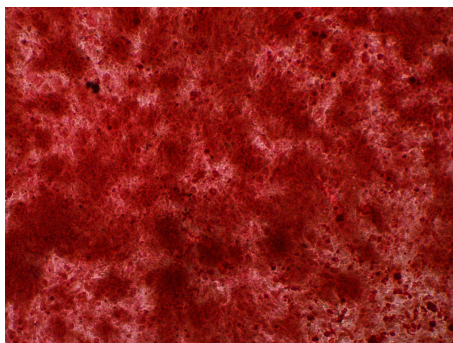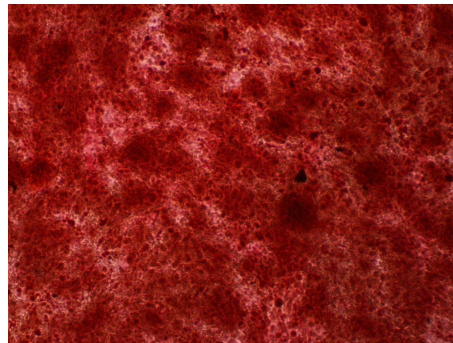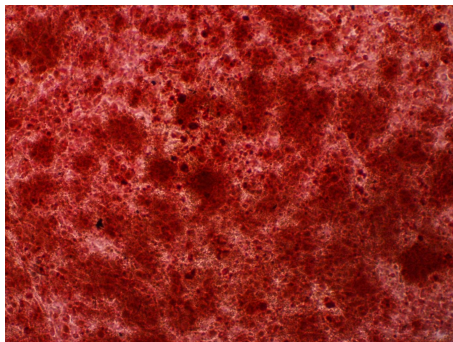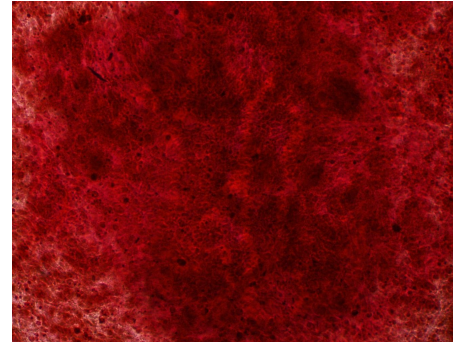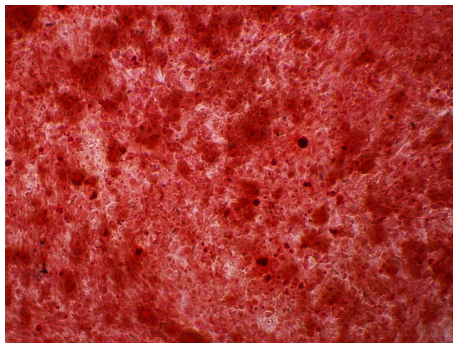

③ Ala+PD

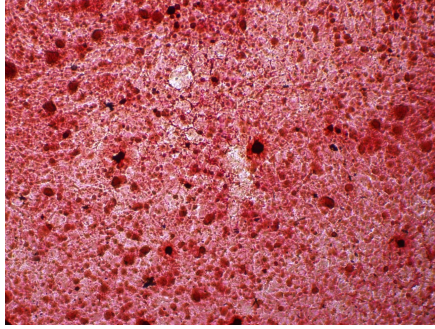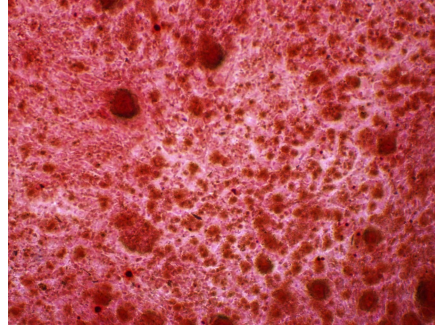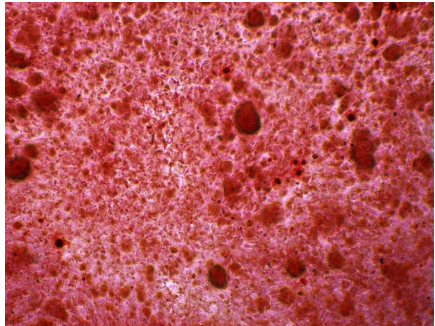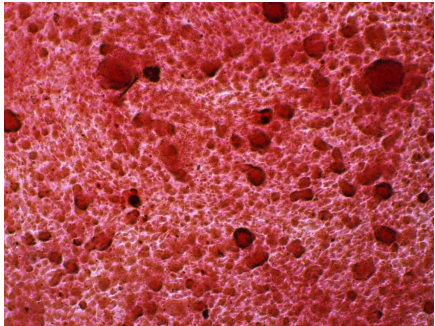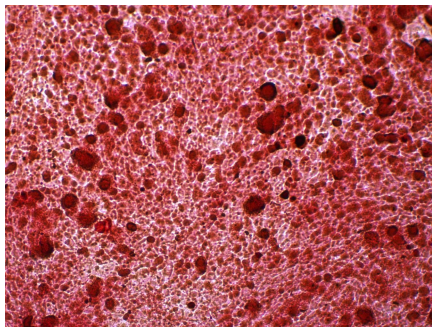

④ Ala+CC

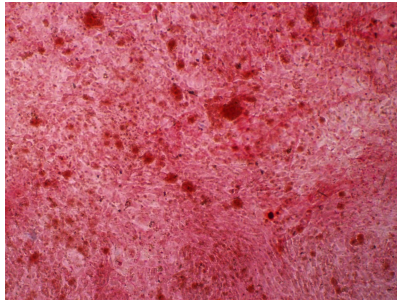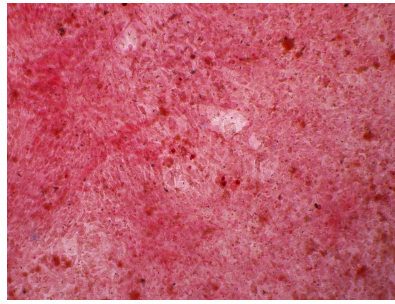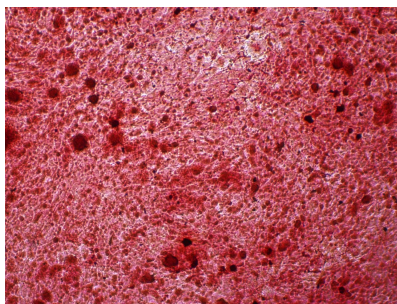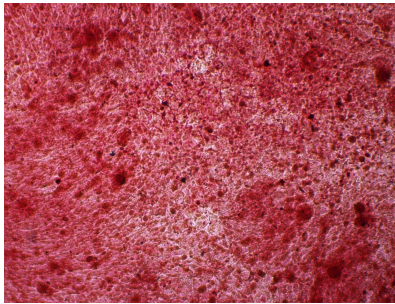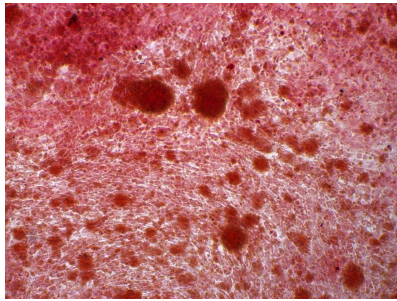

## ALP Staining: ( $\times 10$ )

### ① PBS

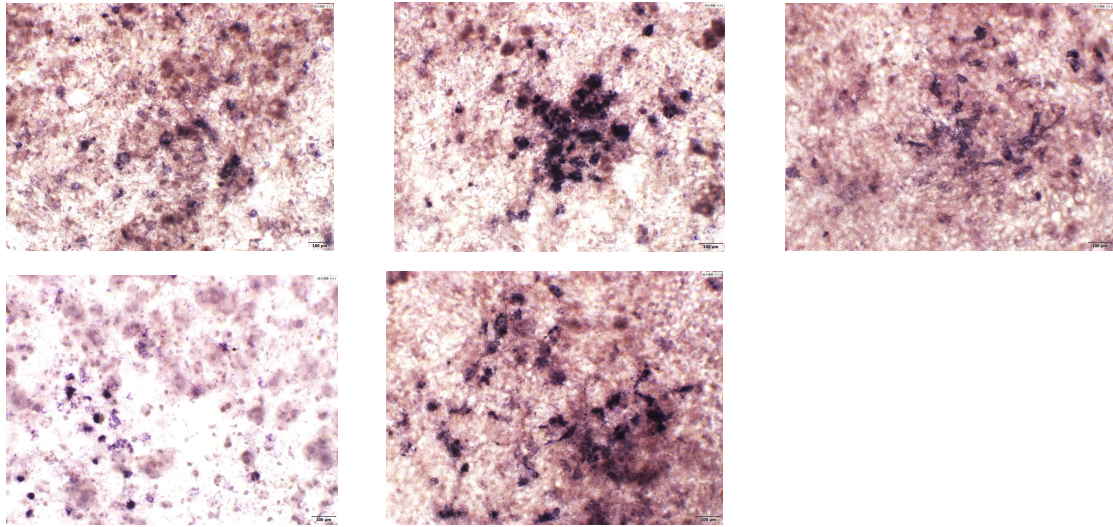

### ② Ala

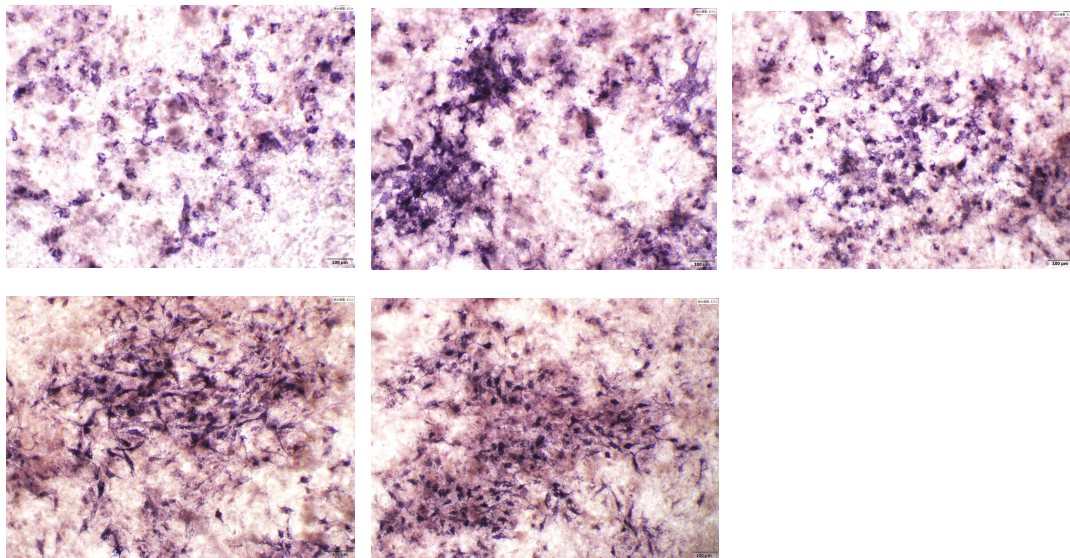

### ③ Ala+PD

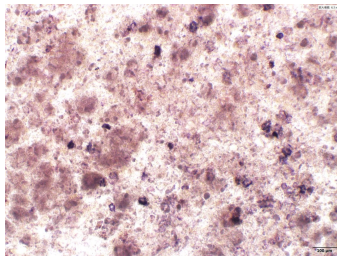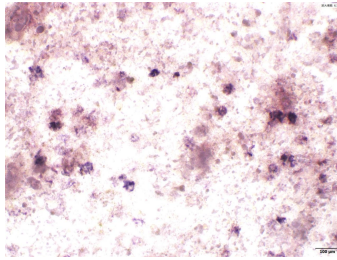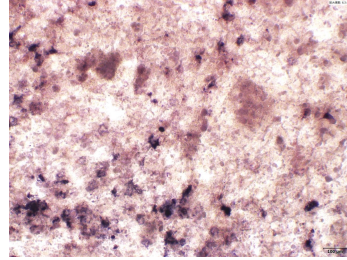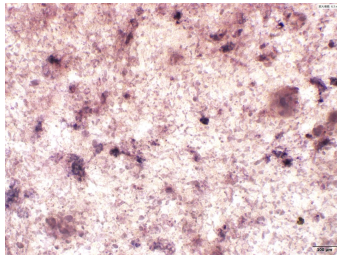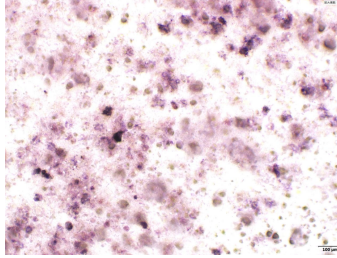

### ④ Ala+CC

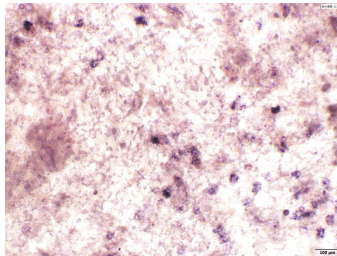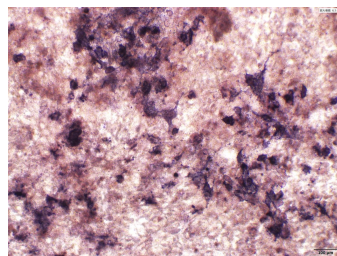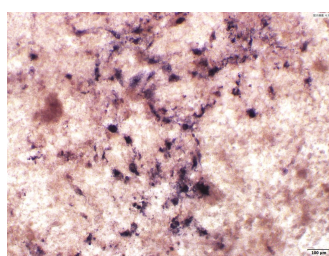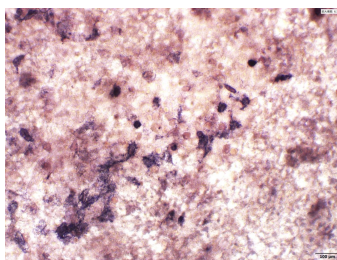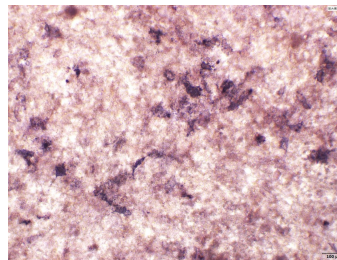

Supplement: Supplementary file 1 — Supplementary Material 1 [file 12891_2023_7159_MOESM1_ESM.pdf]
