# Supplementary material for: Alamandine attenuates ovariectomy-induced osteoporosis by promoting osteogenic differentiation via AMPK/eNOS axis
Source: BMC Musculoskelet Disord. 2024 Jan 10;25:45. doi: 10.1186/s12891-023-07159-2 (PMC10777585; doi:10.1186/s12891-023-07159-2)
Supplement: Supplementary file 2 — Supplementary Material 2 [file 12891_2023_7159_MOESM2_ESM.pdf]

The blots in the red boxes were used in the **Main Figure**.

MrgD-1

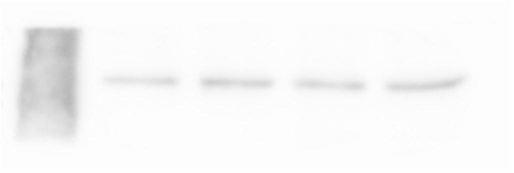

(Exposure for 30 seconds)

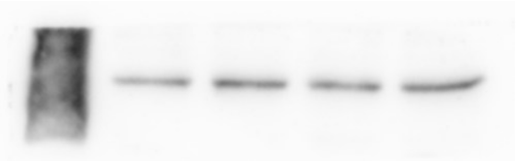

(Exposure for 60 seconds)

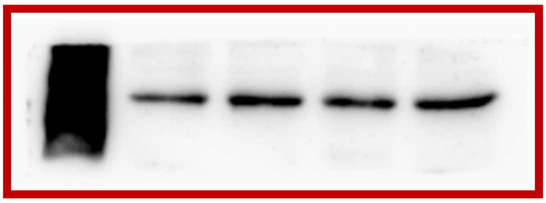

(Exposure for 180 seconds)

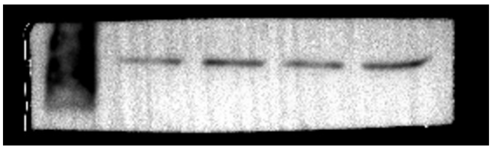

(Merge)

MrgD-2

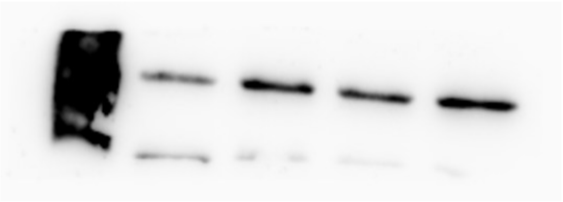

(Exposure for 180 seconds)

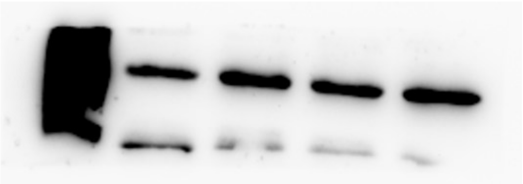

(Exposure for 300 seconds)

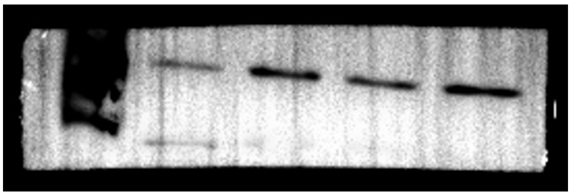

(Merge)

MrgD-3

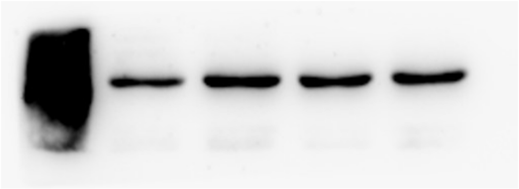

(Exposure for 180 seconds)

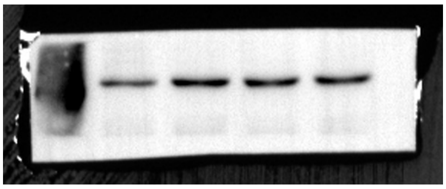

(Merge)

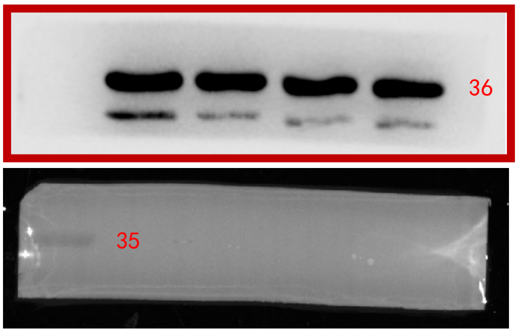

(GD)

RUNX2-1

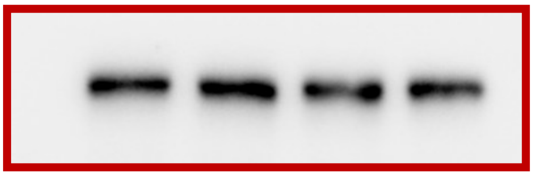

(Exposure for 10 seconds)

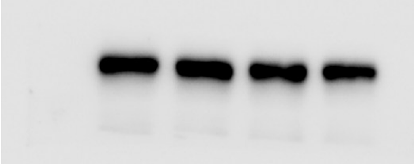

(Exposure for 20 seconds)

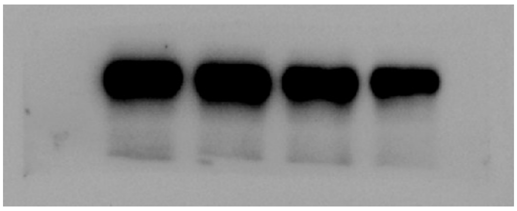

(Exposure for 30 seconds)

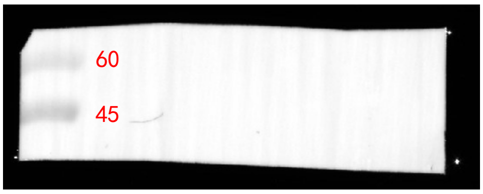

(Marker)

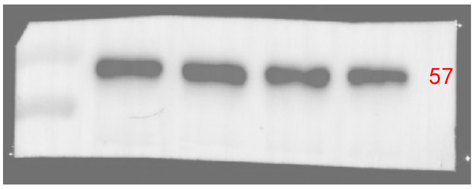

(Merge)

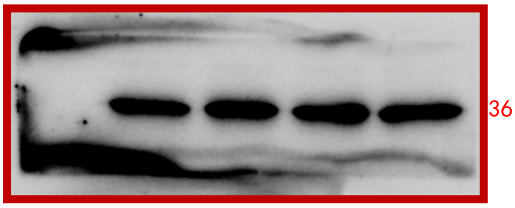

(GD)

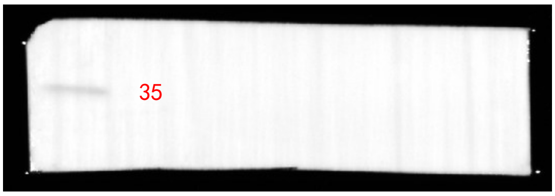

(GD-Marker)

RUNX2-2

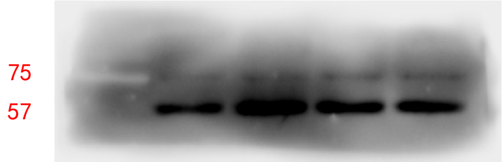

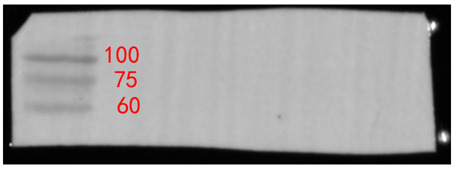

(Marker)

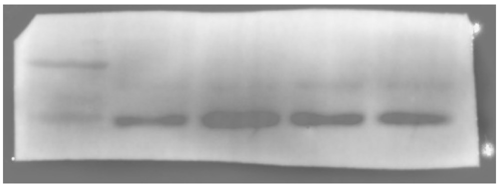

(Merge)

RUNX2-3

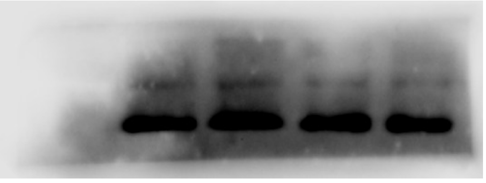

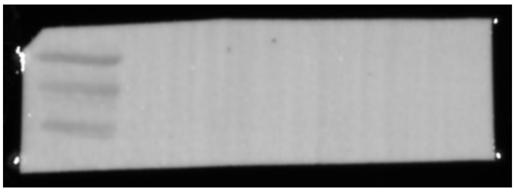

(Marker)

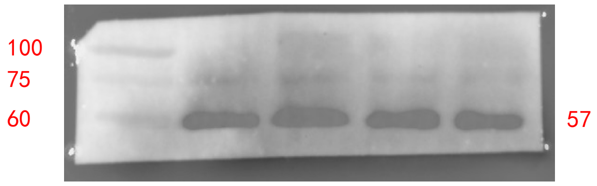

(Merge)

OPN-1

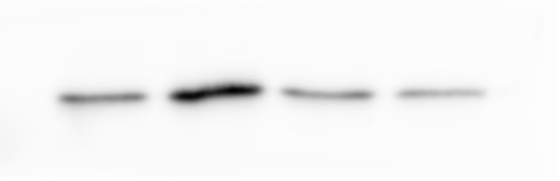

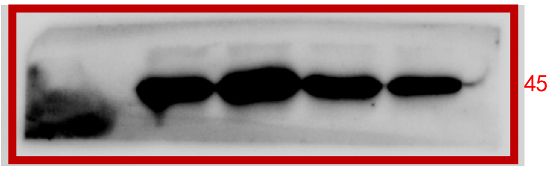

(Exposure for 10 seconds)

(Exposure for 120 seconds)

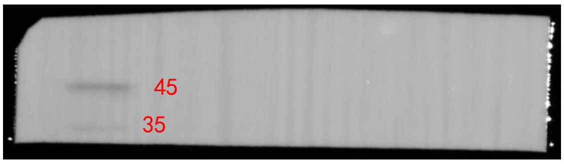

(Marker)

OPN-2

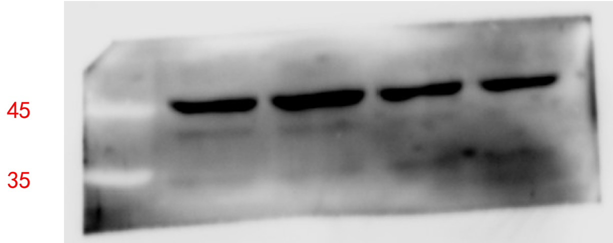

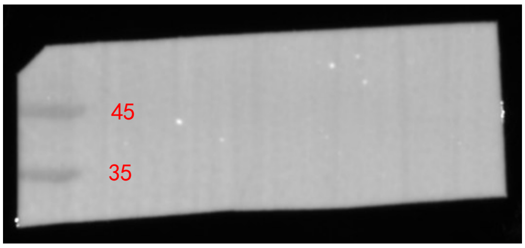

(Marker)

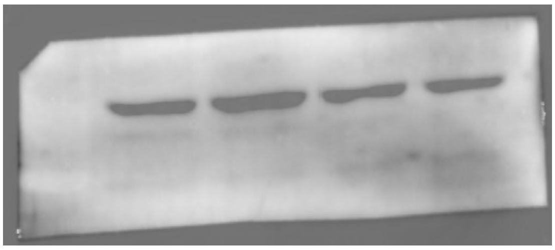

(Merge)

OPN-3

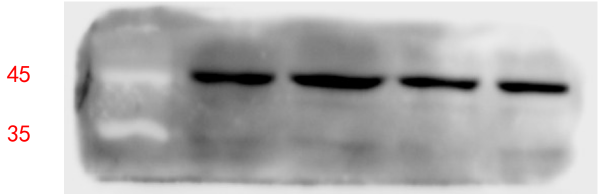

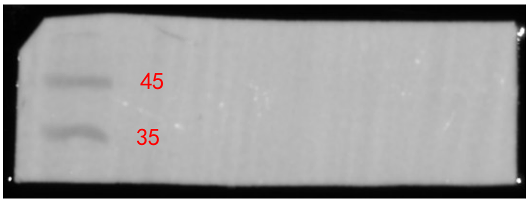

(Marker)

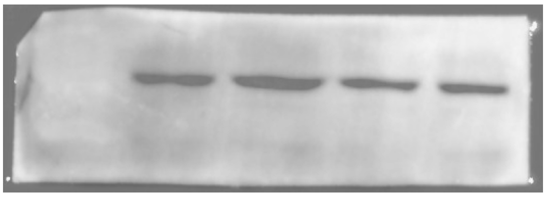

(Merge)

COL1A1-1

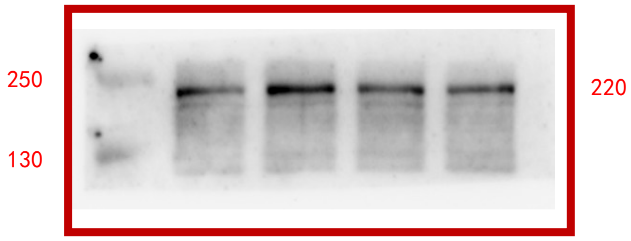

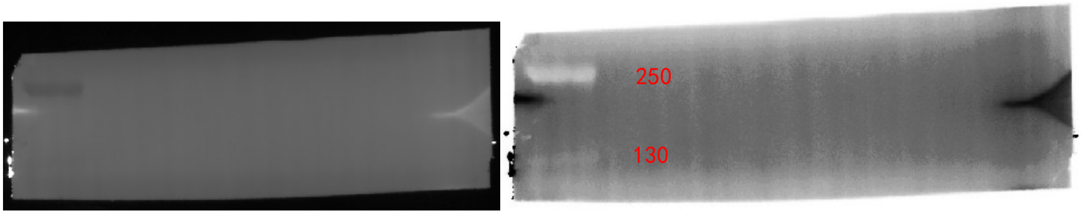

(Marker)

COL1A1-2

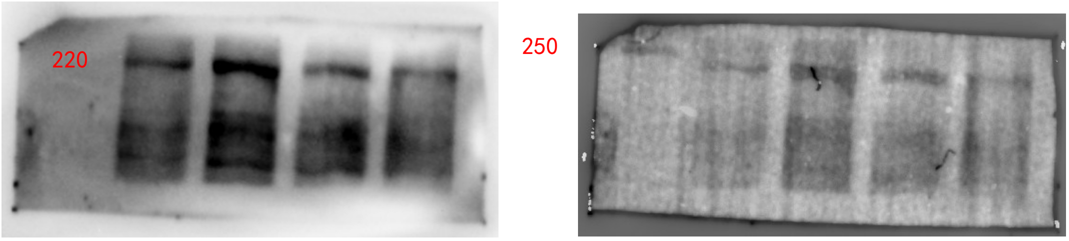

(Merge)

COL1A1-3

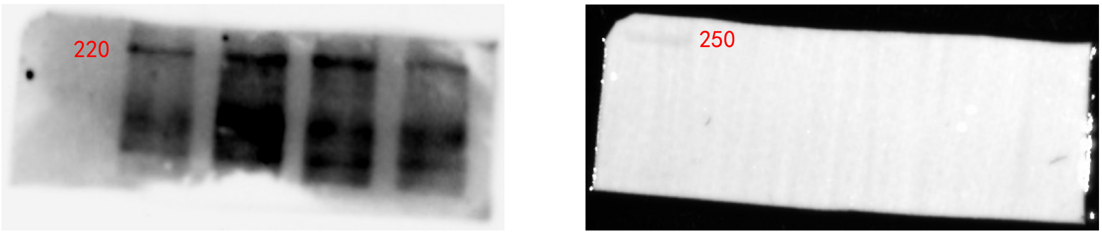

(Marker)

*Due to prolonged electrotransfer, the 130kda marker on the membrane may become difficult to see clearly.
