# Supplementary material for: Alamandine attenuates ovariectomy-induced osteoporosis by promoting osteogenic differentiation via AMPK/eNOS axis
Source: BMC Musculoskelet Disord. 2024 Jan 10;25:45. doi: 10.1186/s12891-023-07159-2 (PMC10777585; doi:10.1186/s12891-023-07159-2)
Supplement: Supplementary file 3 — Supplementary Material 3 [file 12891_2023_7159_MOESM3_ESM.pdf]

The blots in the red boxes were used in the **Main Figure**.

pho-AMPKα ①

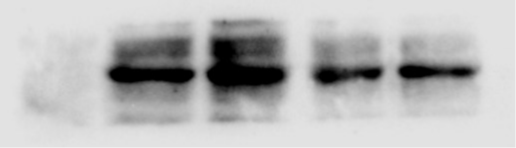

(pho-AMPKα-①)

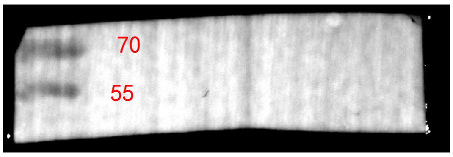

(pho-AMPKα-① Marker)

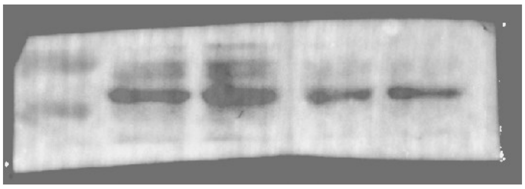

62

(Merge)

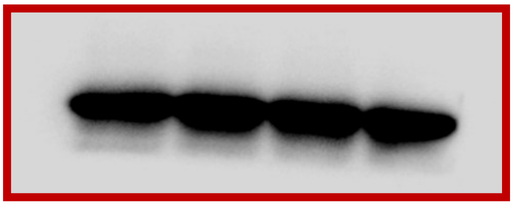

36

(GD)

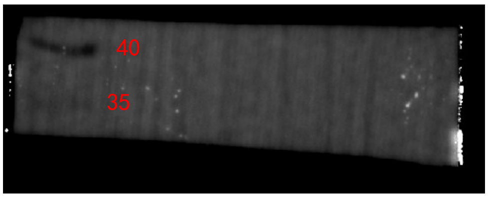

(GD-Marker)

Re-probed:

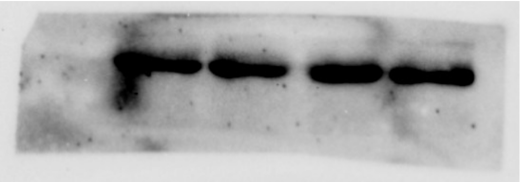

(AMPKα-①)

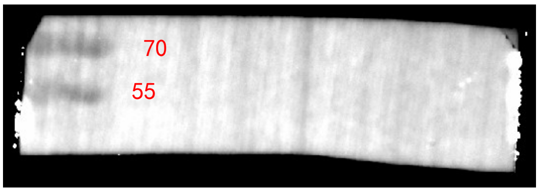

(Marker)

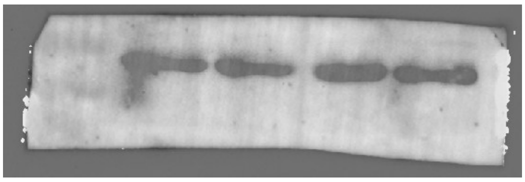

62

(Merge)

pho-AMPKα ②

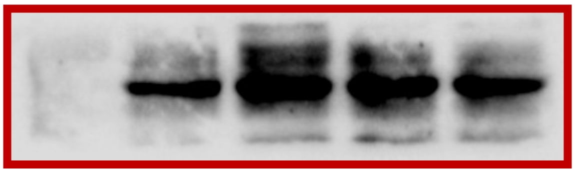

(pho-AMPKα-②)

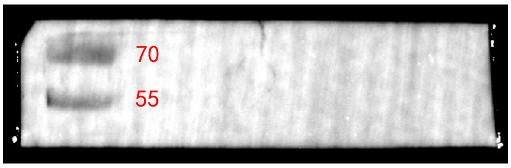

(pho-AMPKα-② Marker)

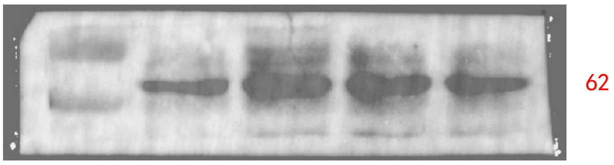

(Merge)

Re-probed:

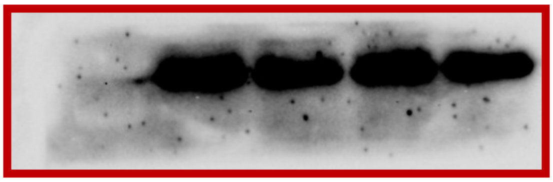

(AMPKα-②)

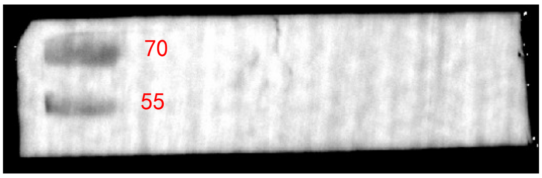

(Marker)

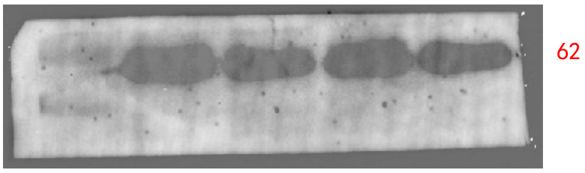

(Merge)

pho-AMPKα ③

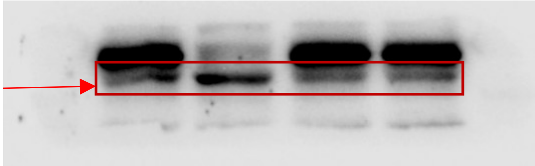

(pho-AMPKα-③)

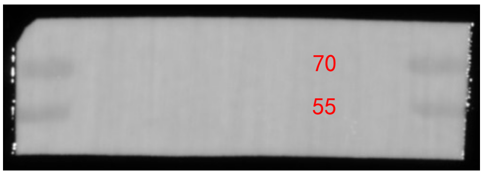

(pho-AMPKα-③ Marker)

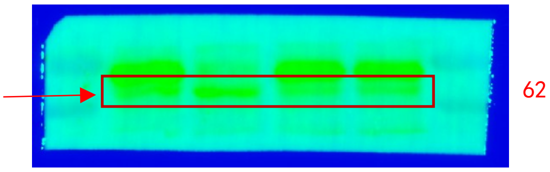

(Merge)

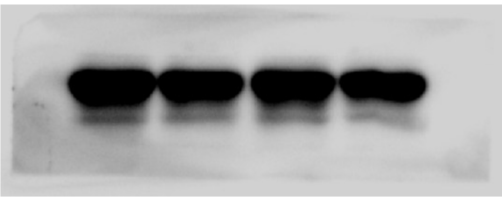

(pho-AMPKα-③ **GD**)

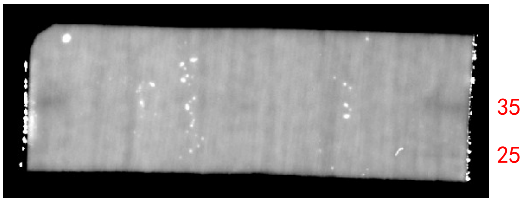

(**GD**-Marker)

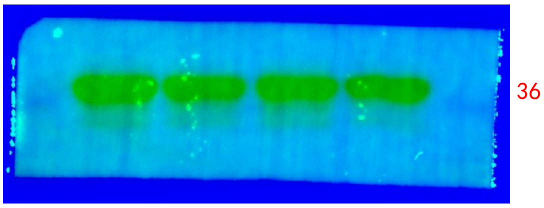

(Merge)

Re-probed:

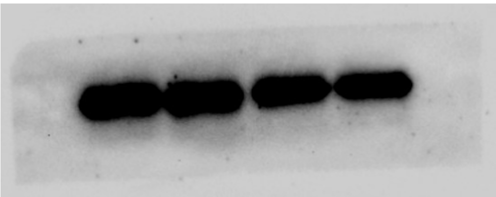

(AMPKα-③)

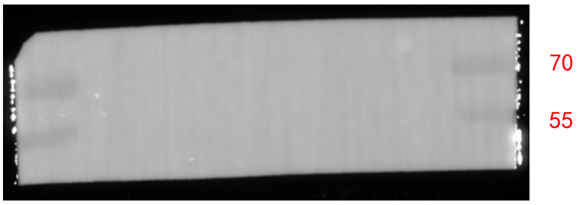

(Marker)

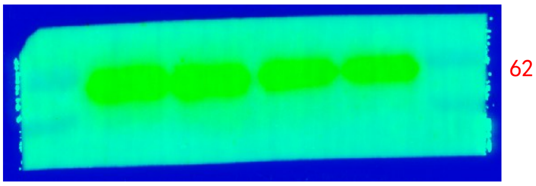

(Merge)

pho-eNOS ①

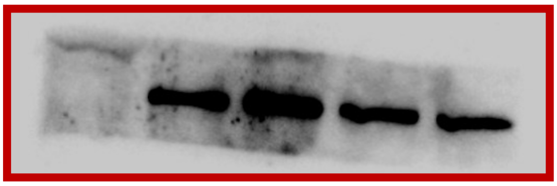

(pho-eNOS-①)

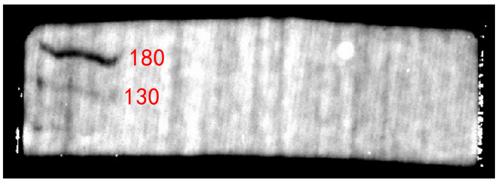

(pho-eNOS-① Marker)

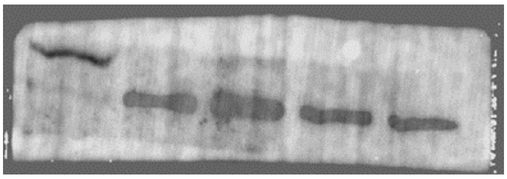

(Merge)

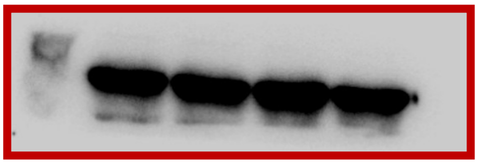

(GD)

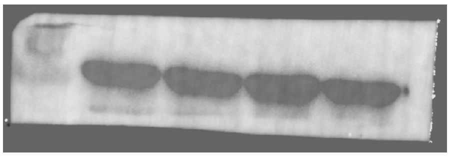

(Merge)

Re-probed:

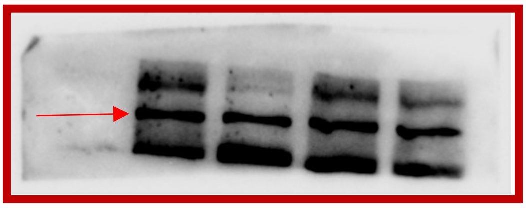

(eNOS-①)

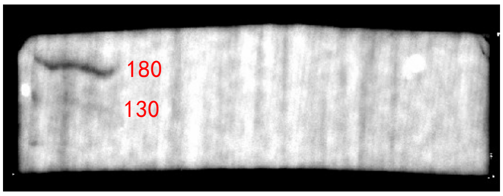

(Marker)

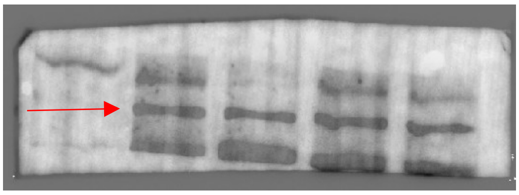

(Merge)

pho-eNOS ②

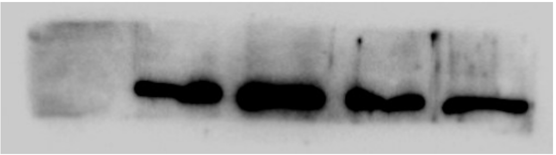

(pho-eNOS-②)

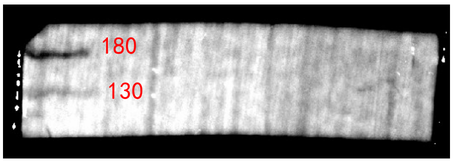

(pho-eNOS-② Marker)

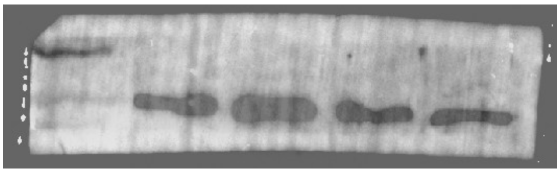

(Merge)

Re-probed:

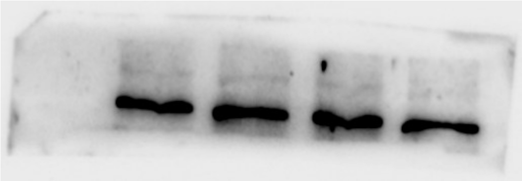

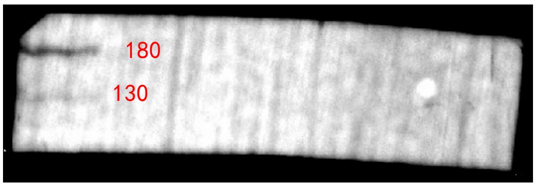

(eNOS-②) (Marker)

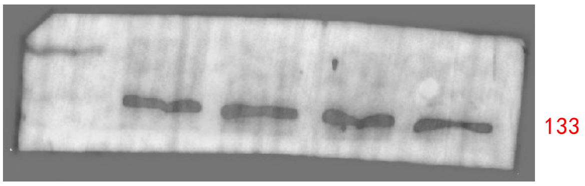

(Merge)

pho-eNOS ③

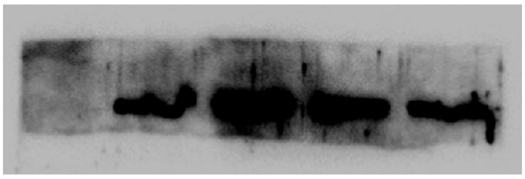

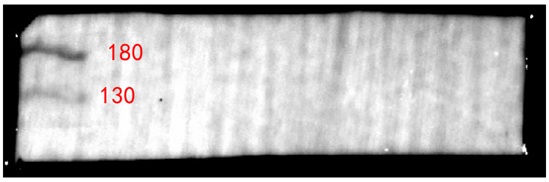

(pho-eNOS-③) (pho-eNOS-③ Marker)

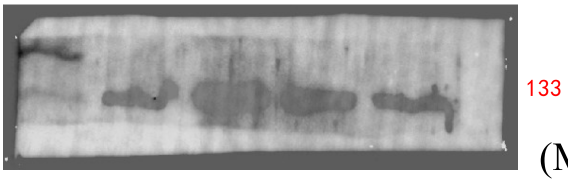

(Merge)

Re-probed:

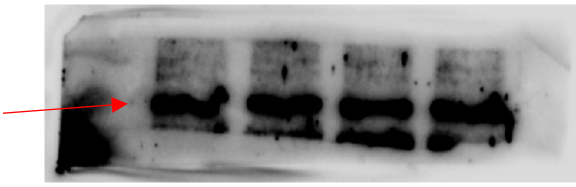

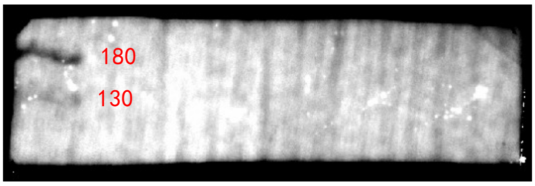

(eNOS-③) (Marker)

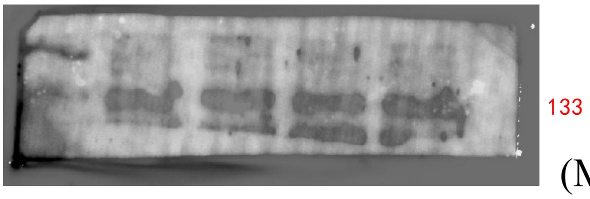

(Merge)
