# Supplementary material for: Alamandine attenuates ovariectomy-induced osteoporosis by promoting osteogenic differentiation via AMPK/eNOS axis
Source: BMC Musculoskelet Disord. 2024 Jan 10;25:45. doi: 10.1186/s12891-023-07159-2 (PMC10777585; doi:10.1186/s12891-023-07159-2)
Supplement: Supplementary file 4 — Supplementary Material 4 [file 12891_2023_7159_MOESM4_ESM.pdf]

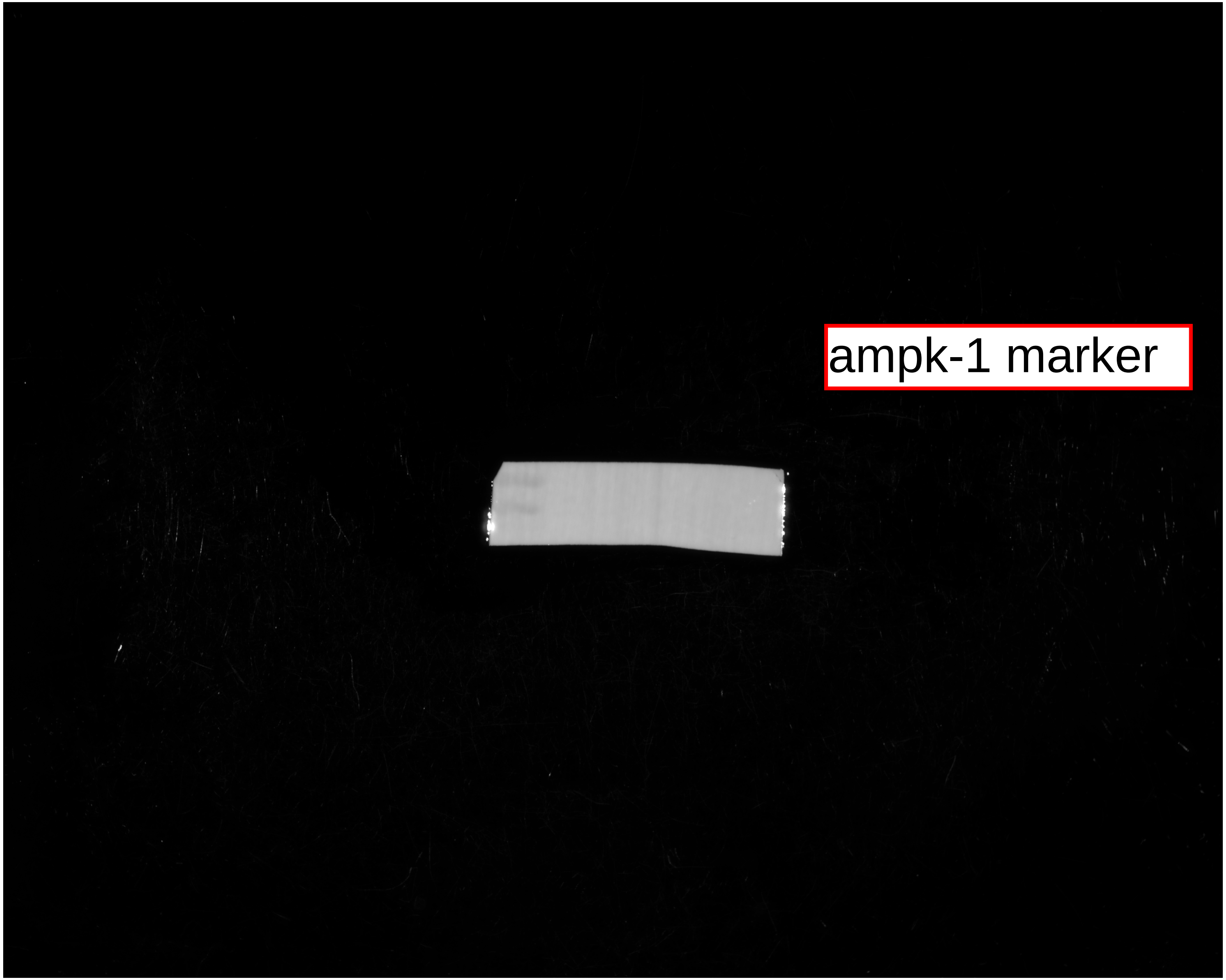
ampk-1 marker

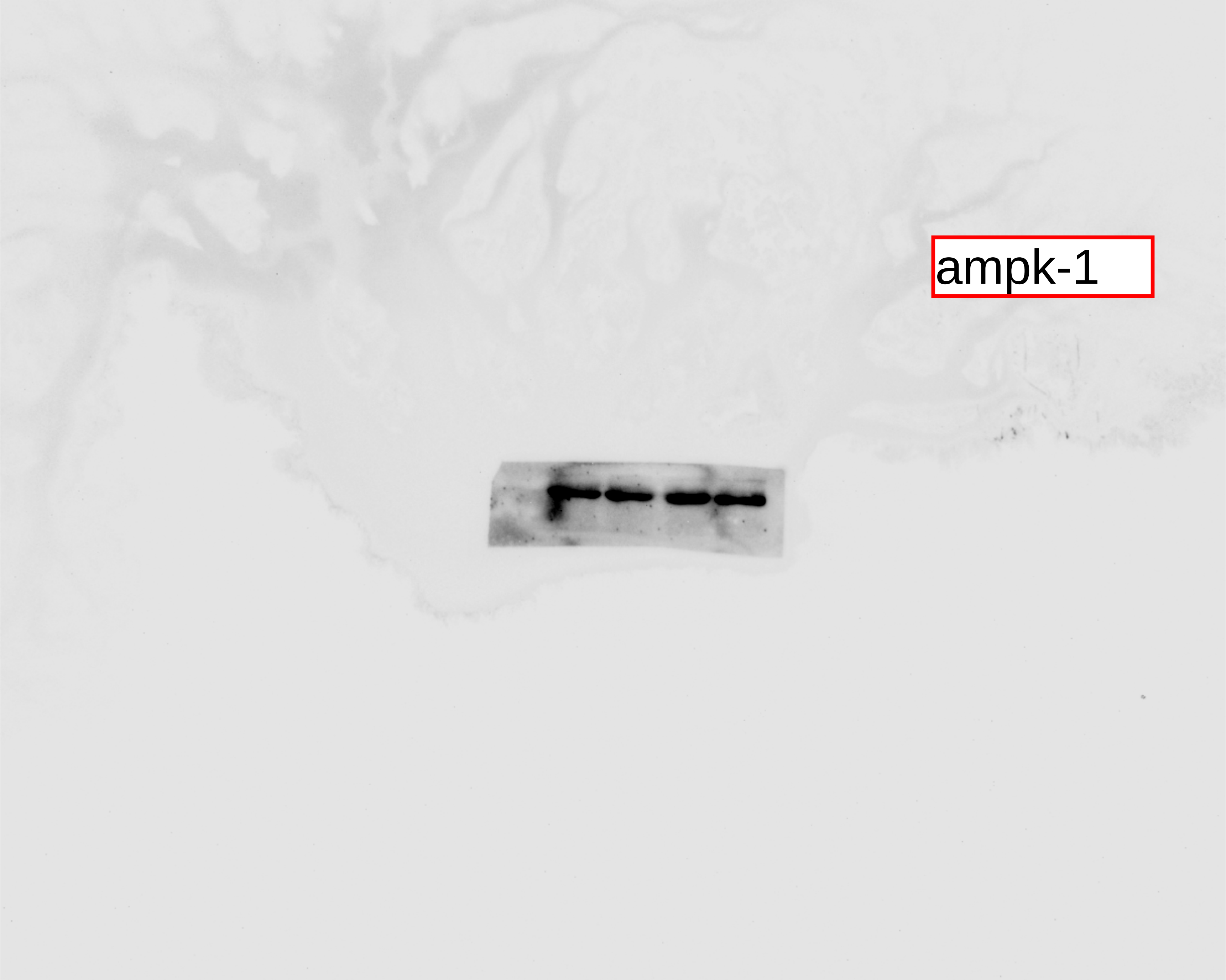
ampk-1

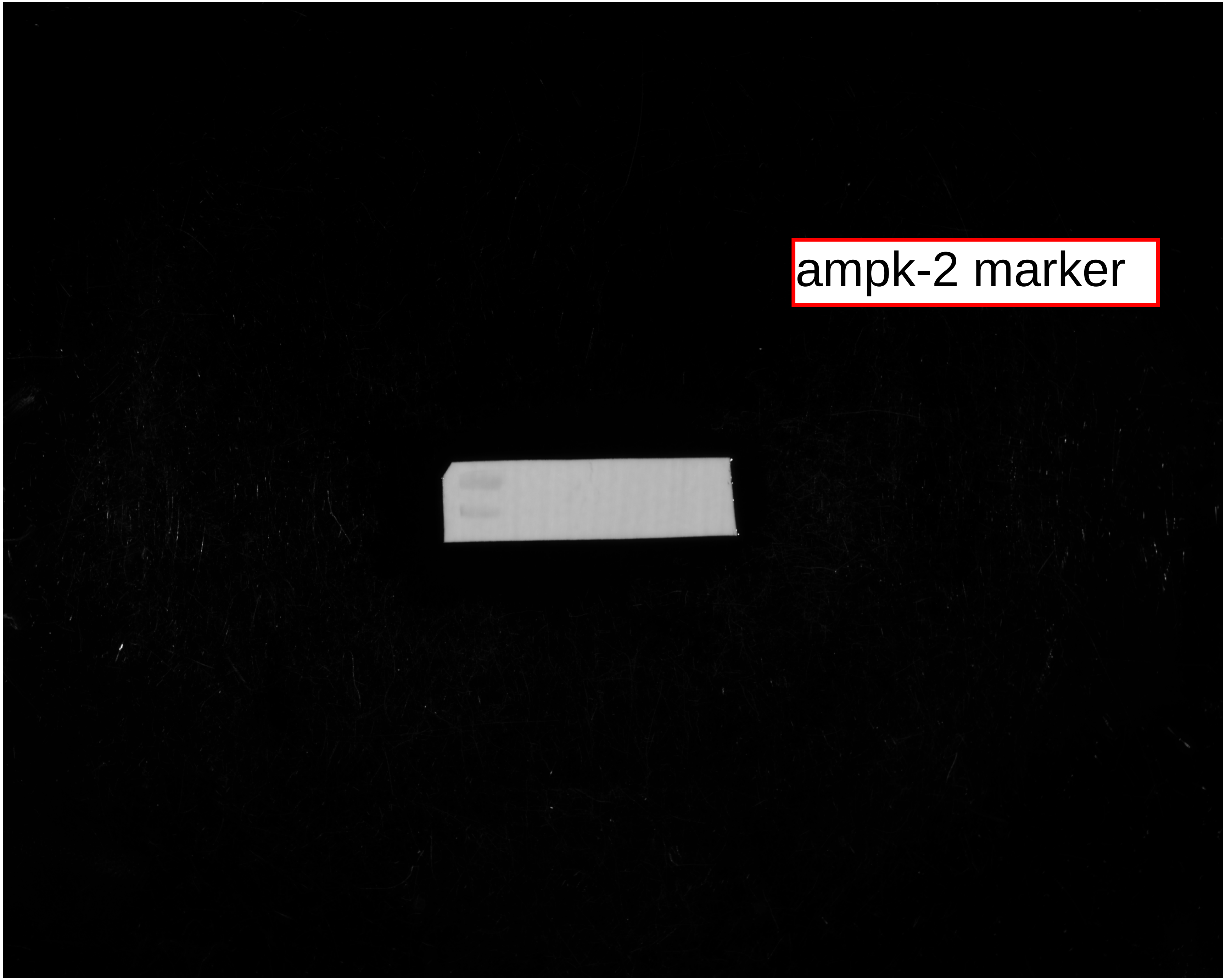
ampk-2 marker

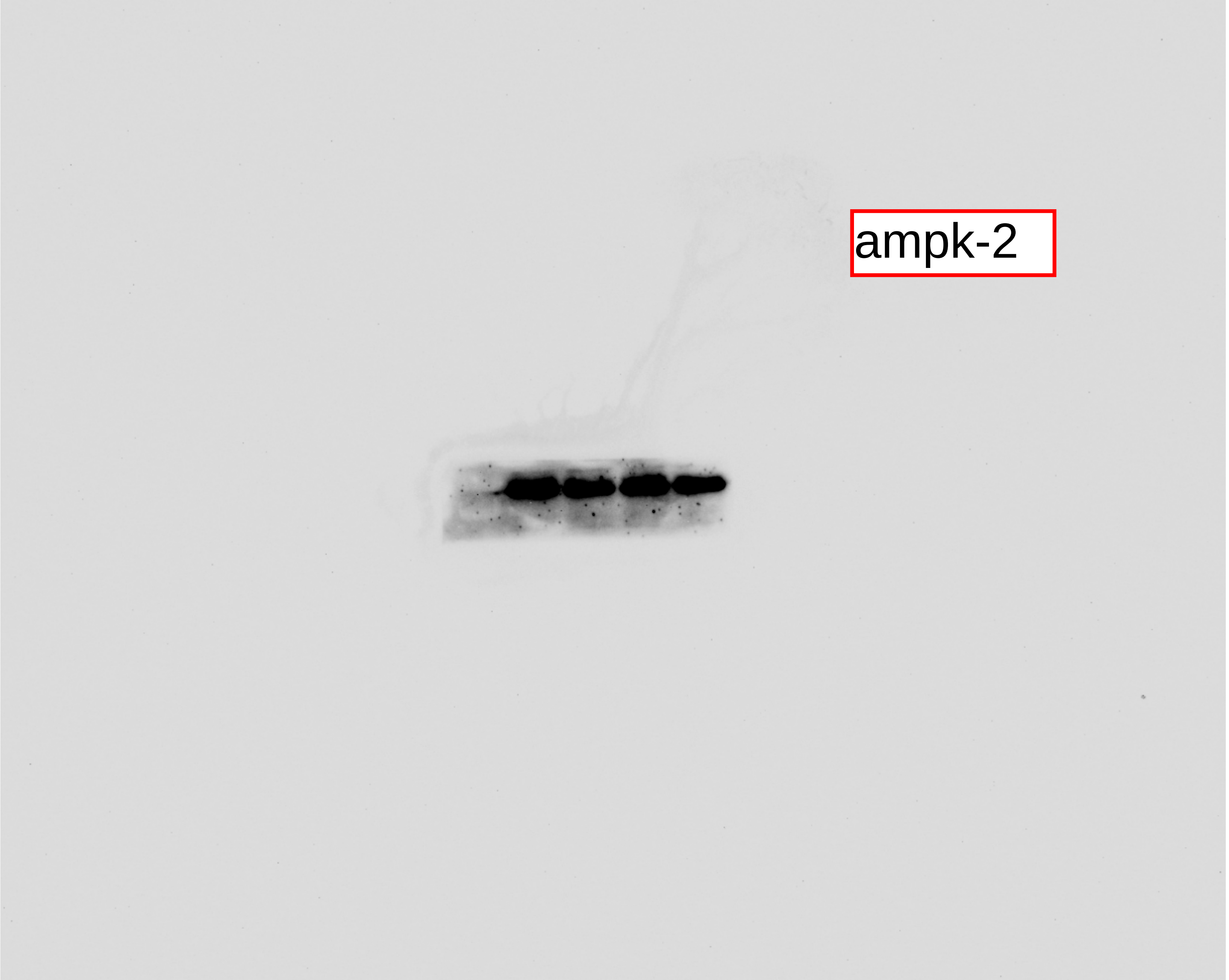
ampk-2

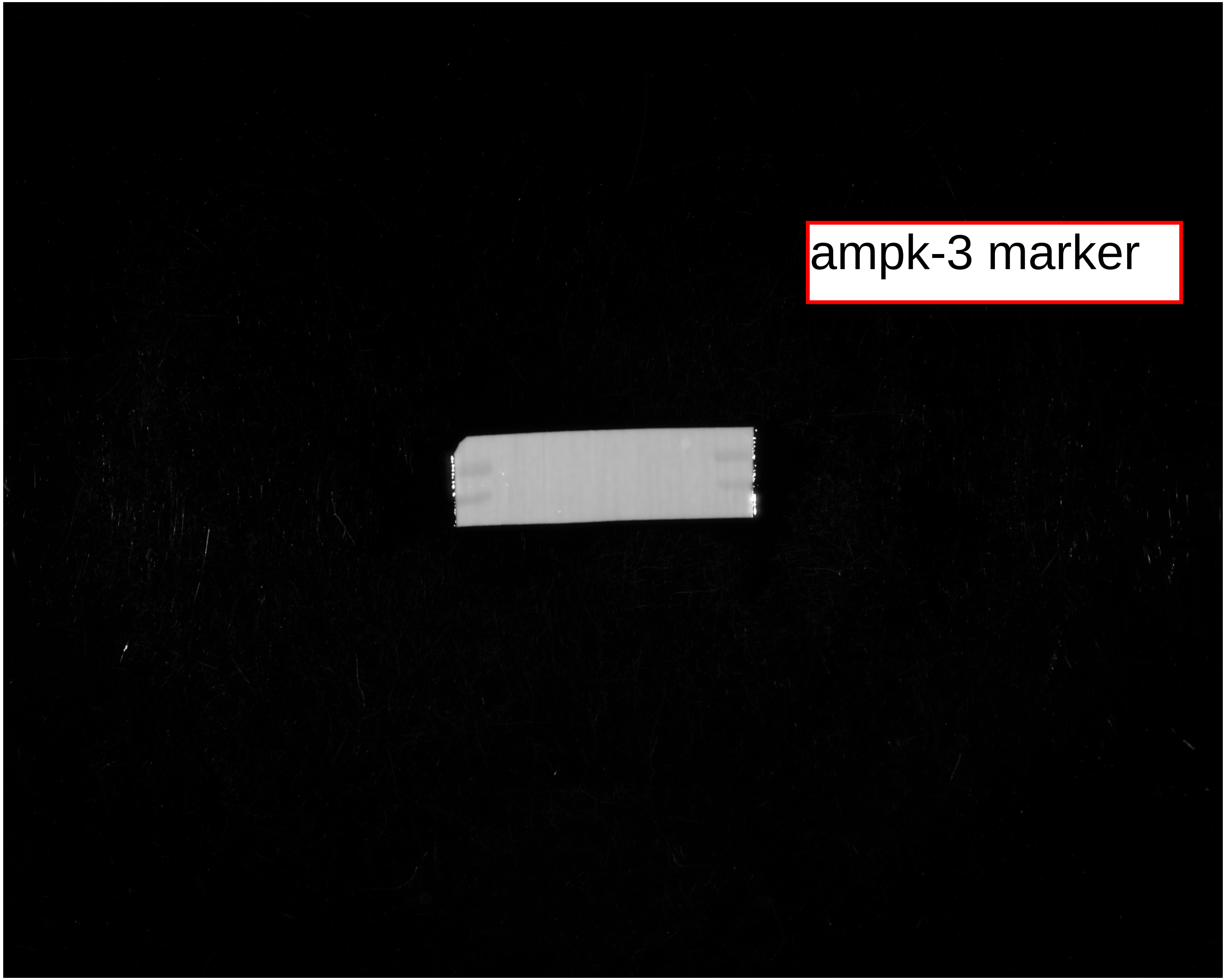
ampk-3 marker

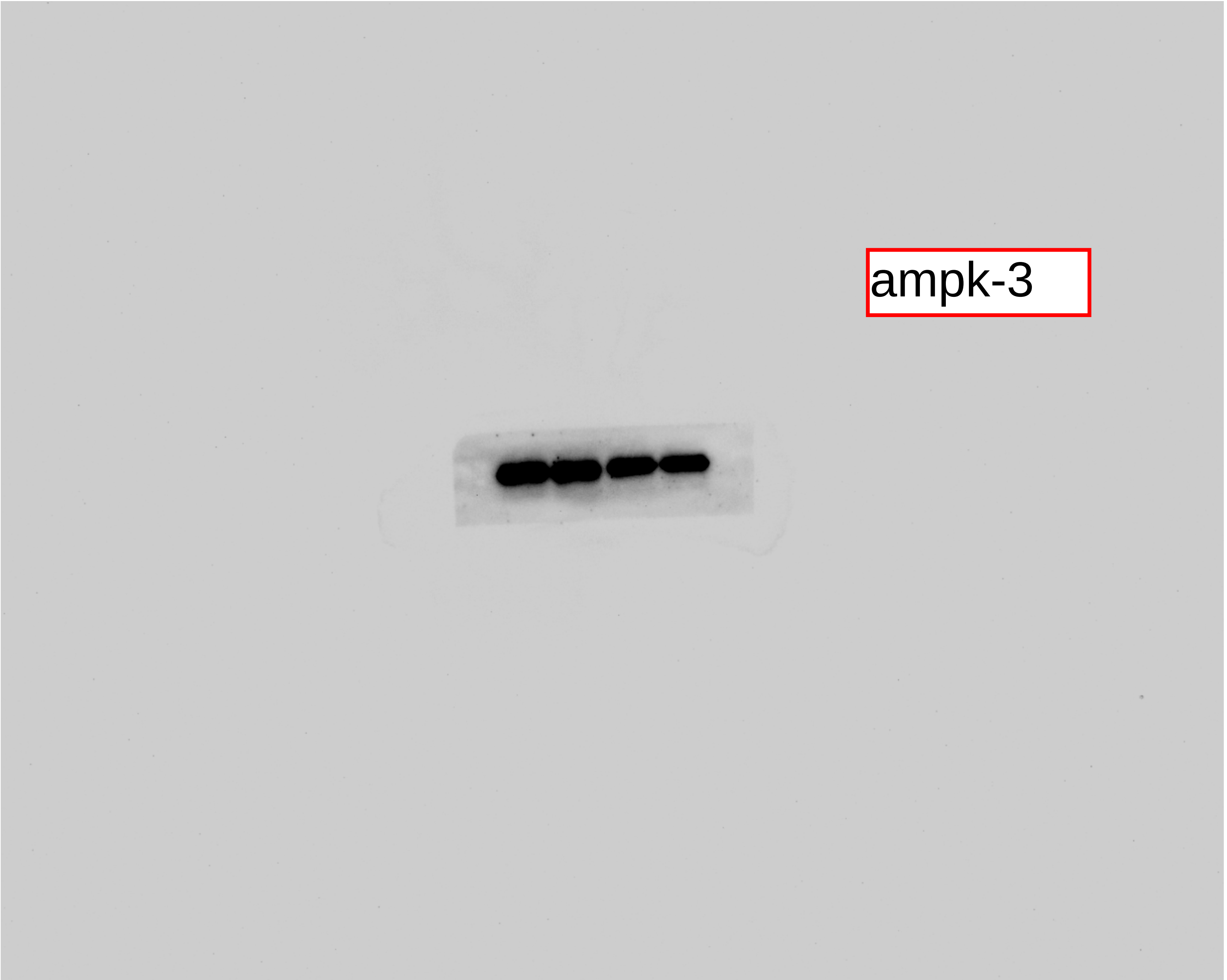
ampk-3

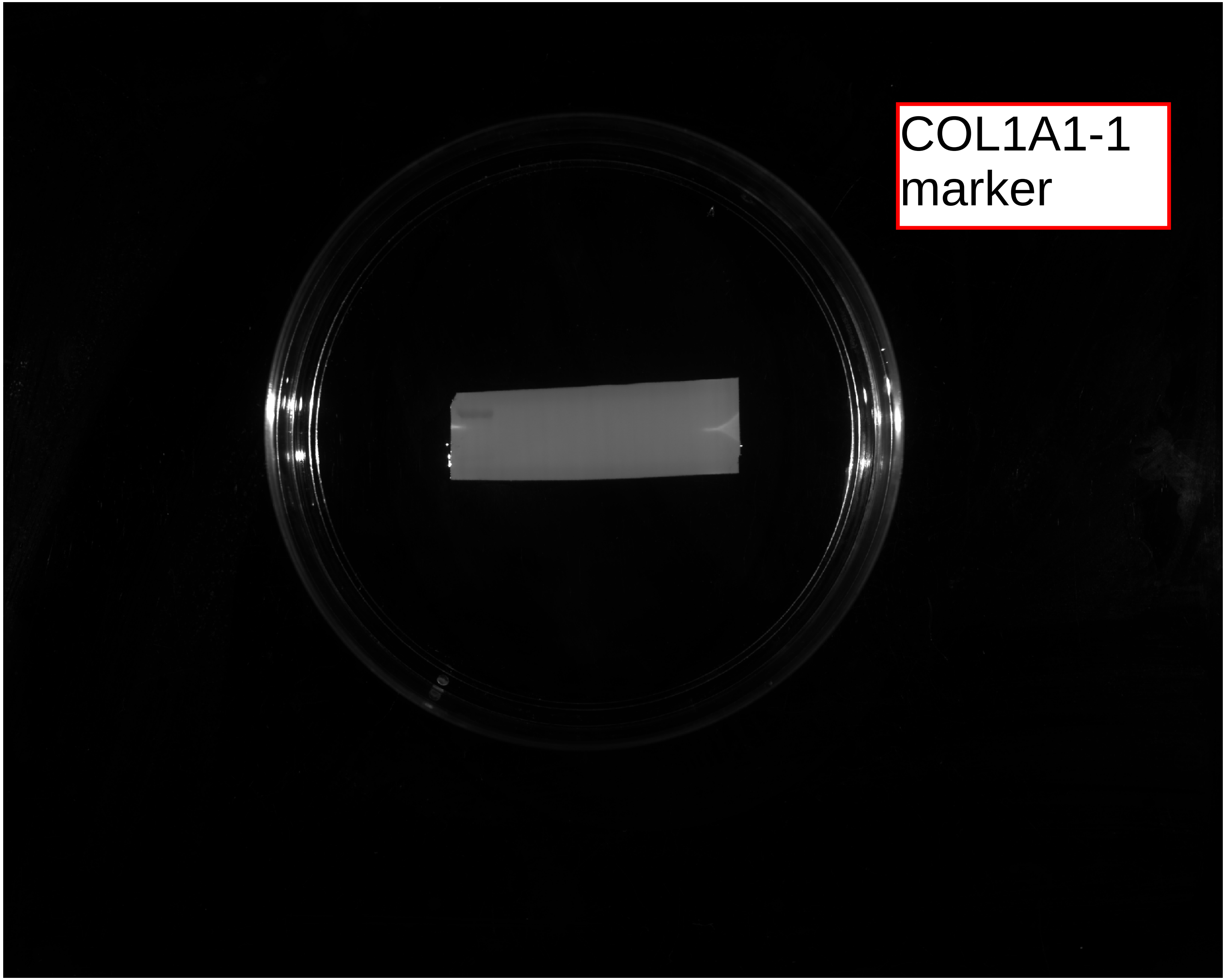
COL1A1-1
marker

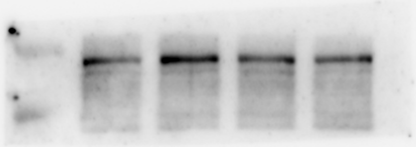

COL1A1-1

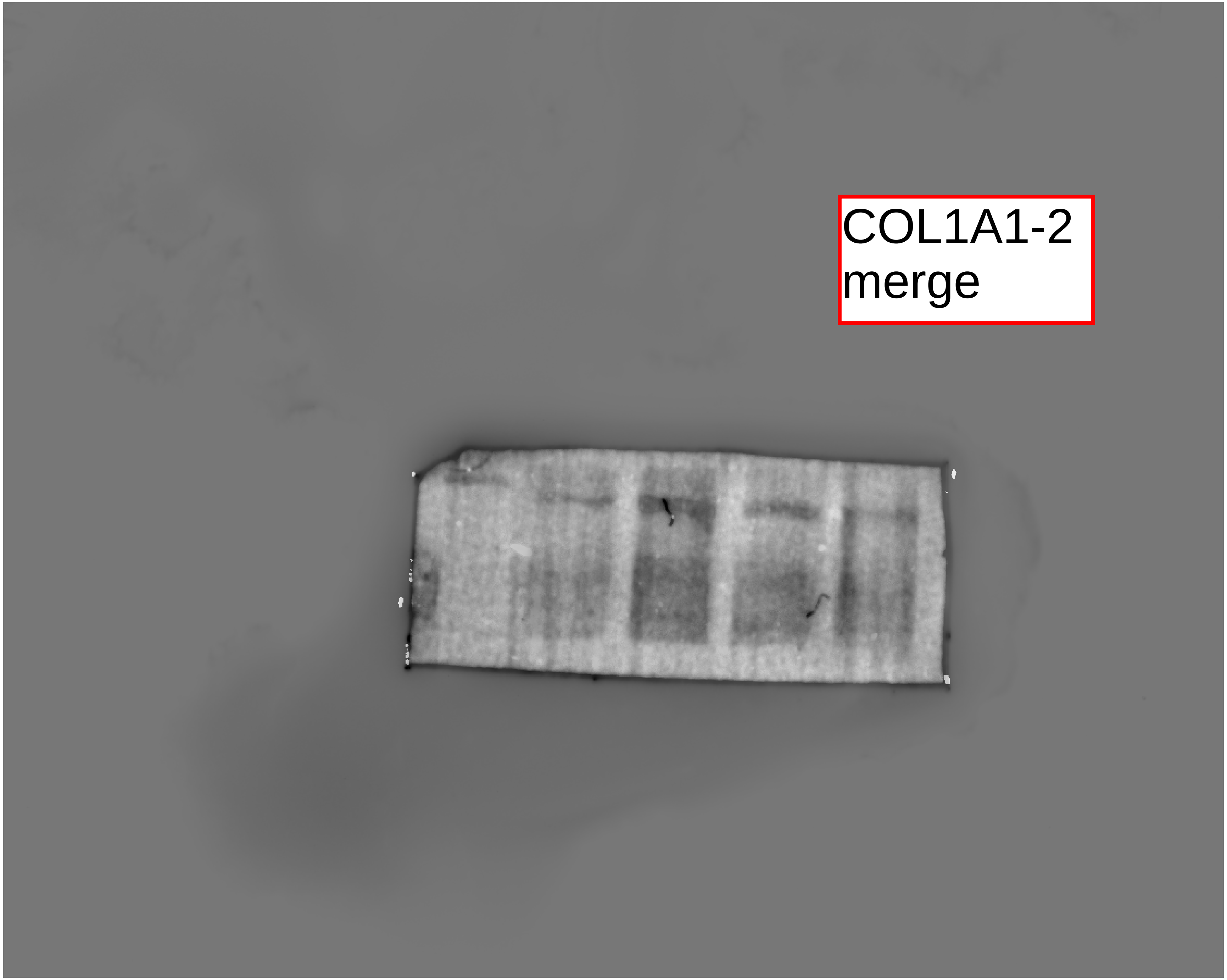
COL1A1-2
merge

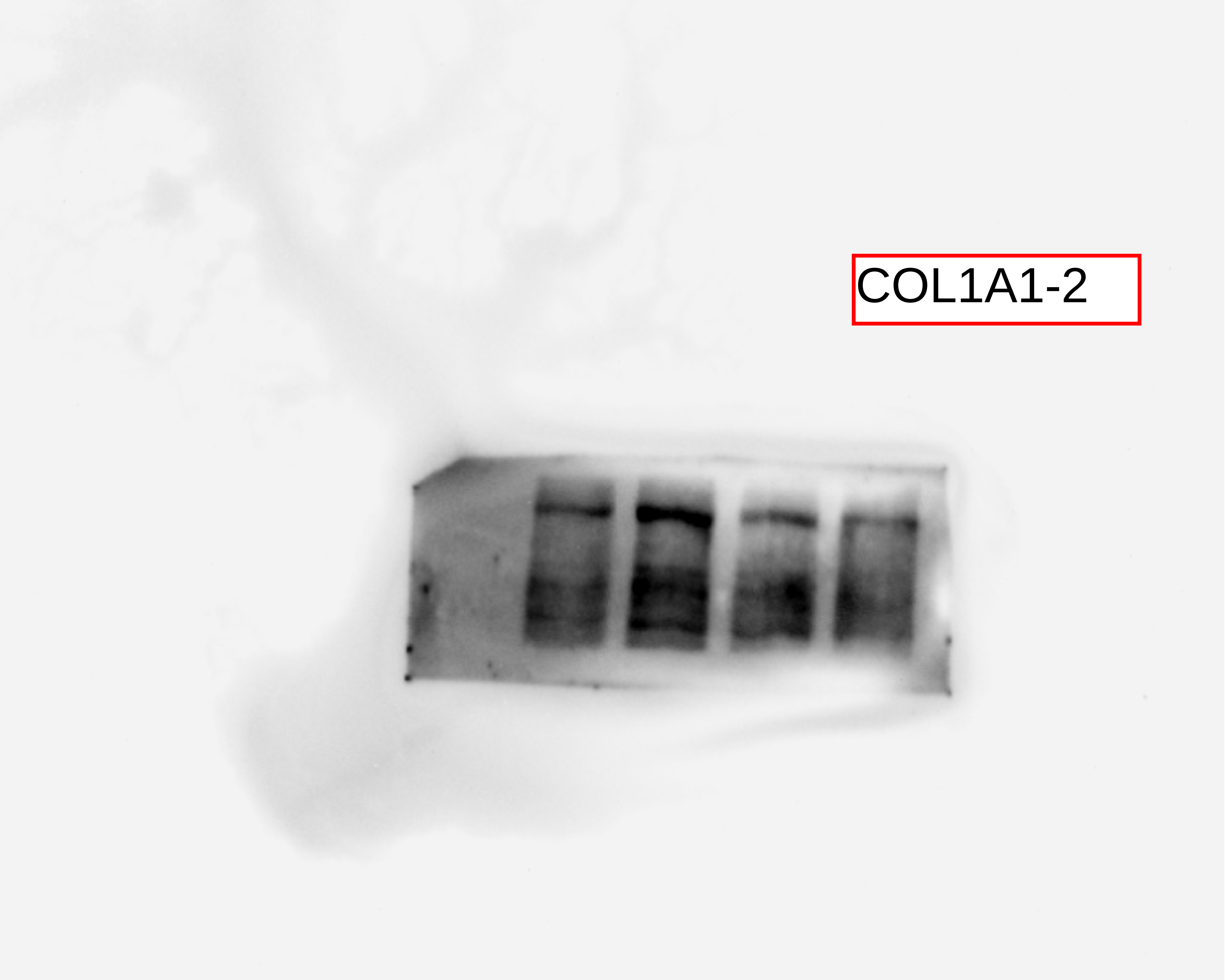
COL1A1-2

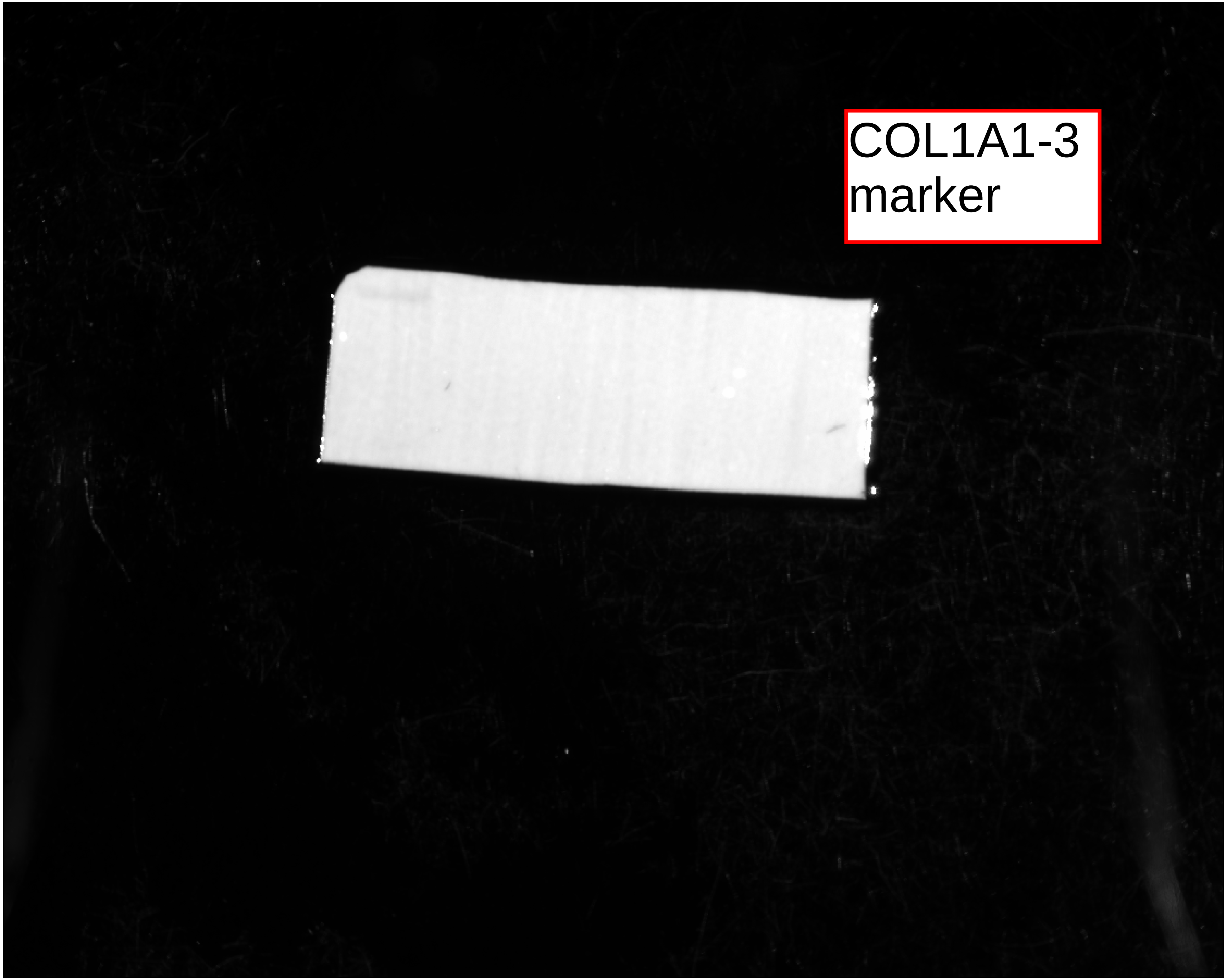
COL1A1-3
marker

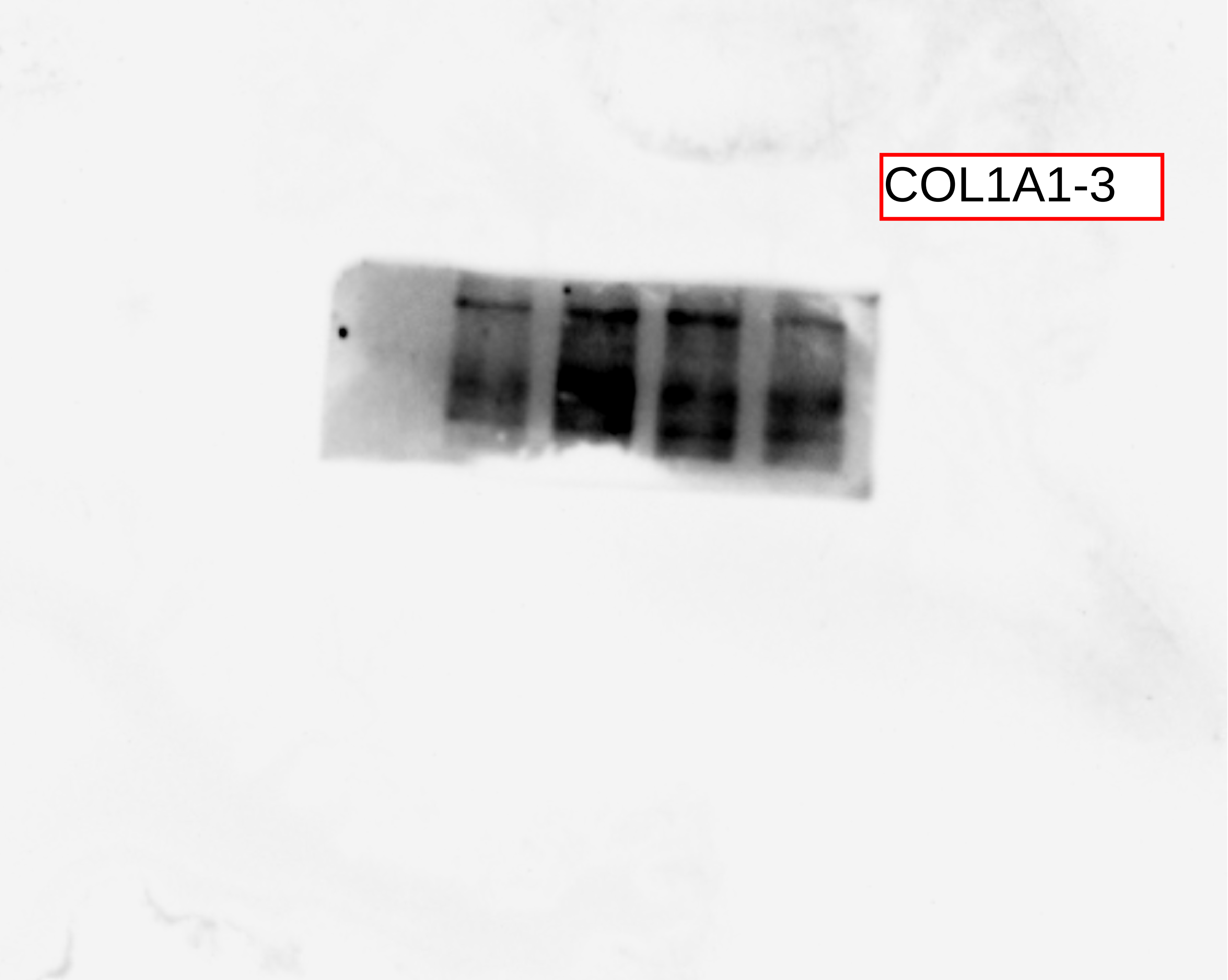
COL1A1-3

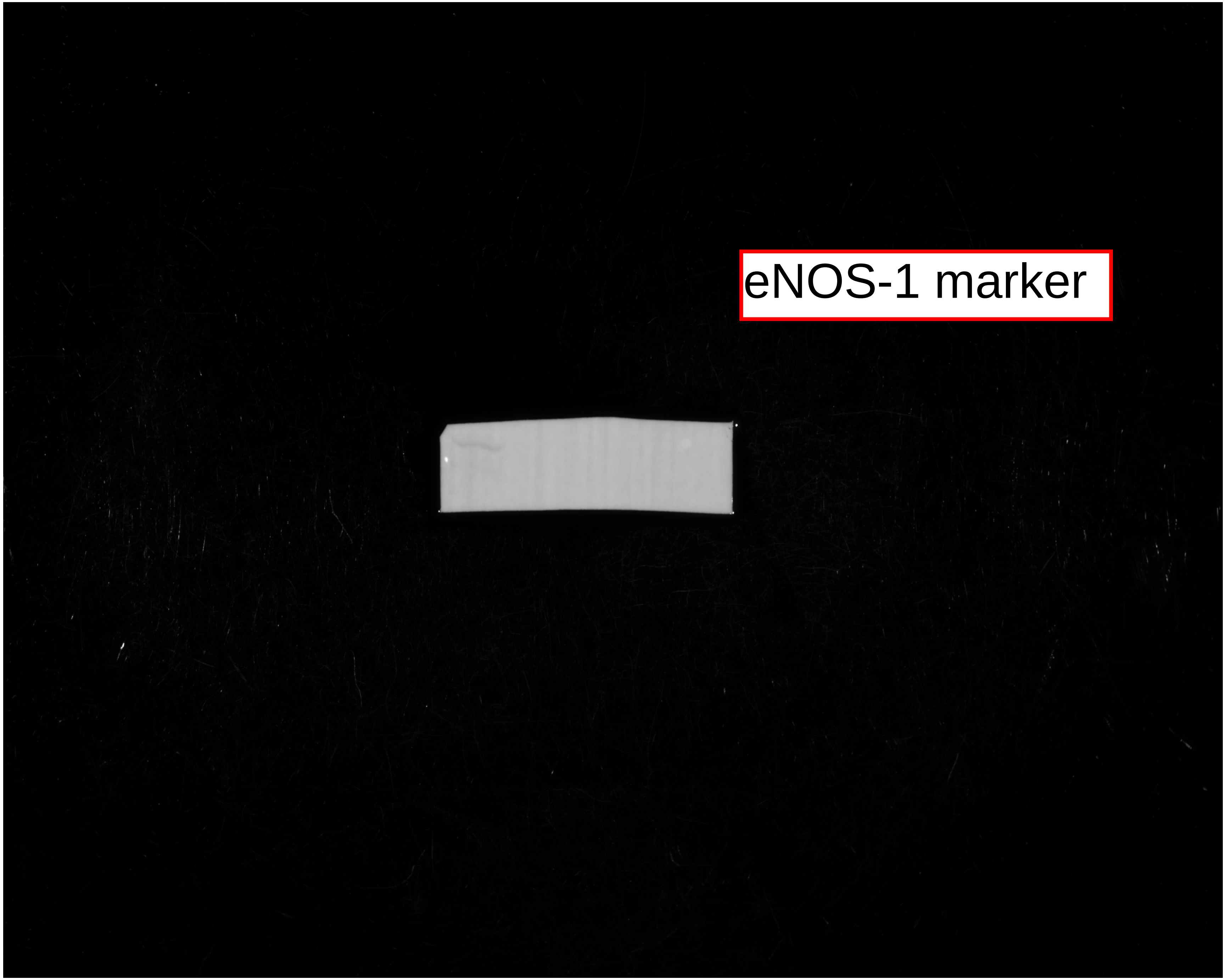
eNOS-1 marker

eNOS-1

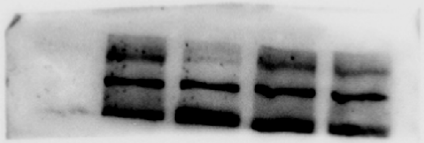

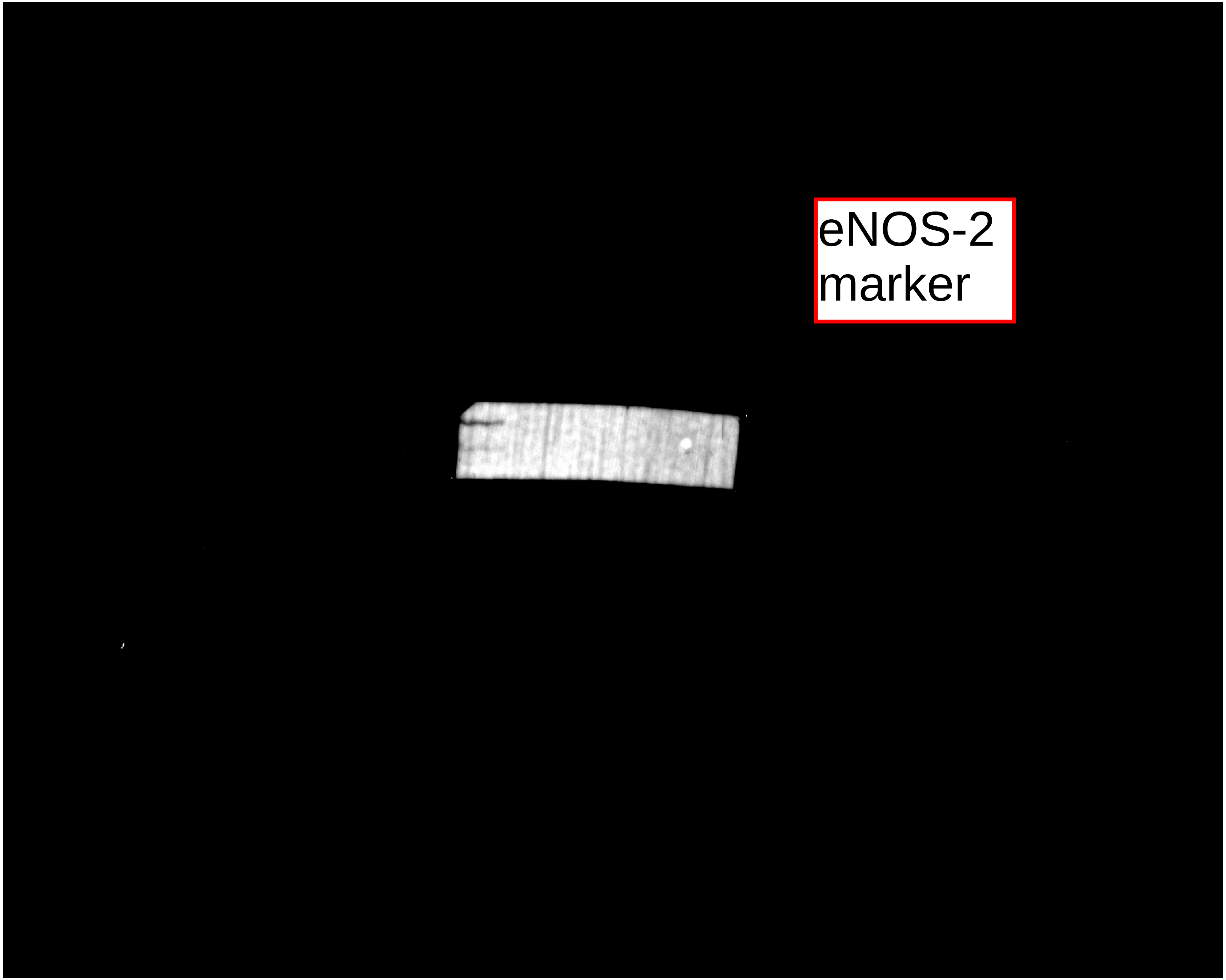
eNOS-2
marker

eNOS-2

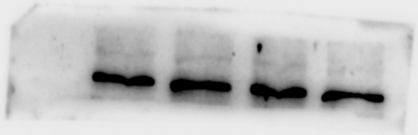

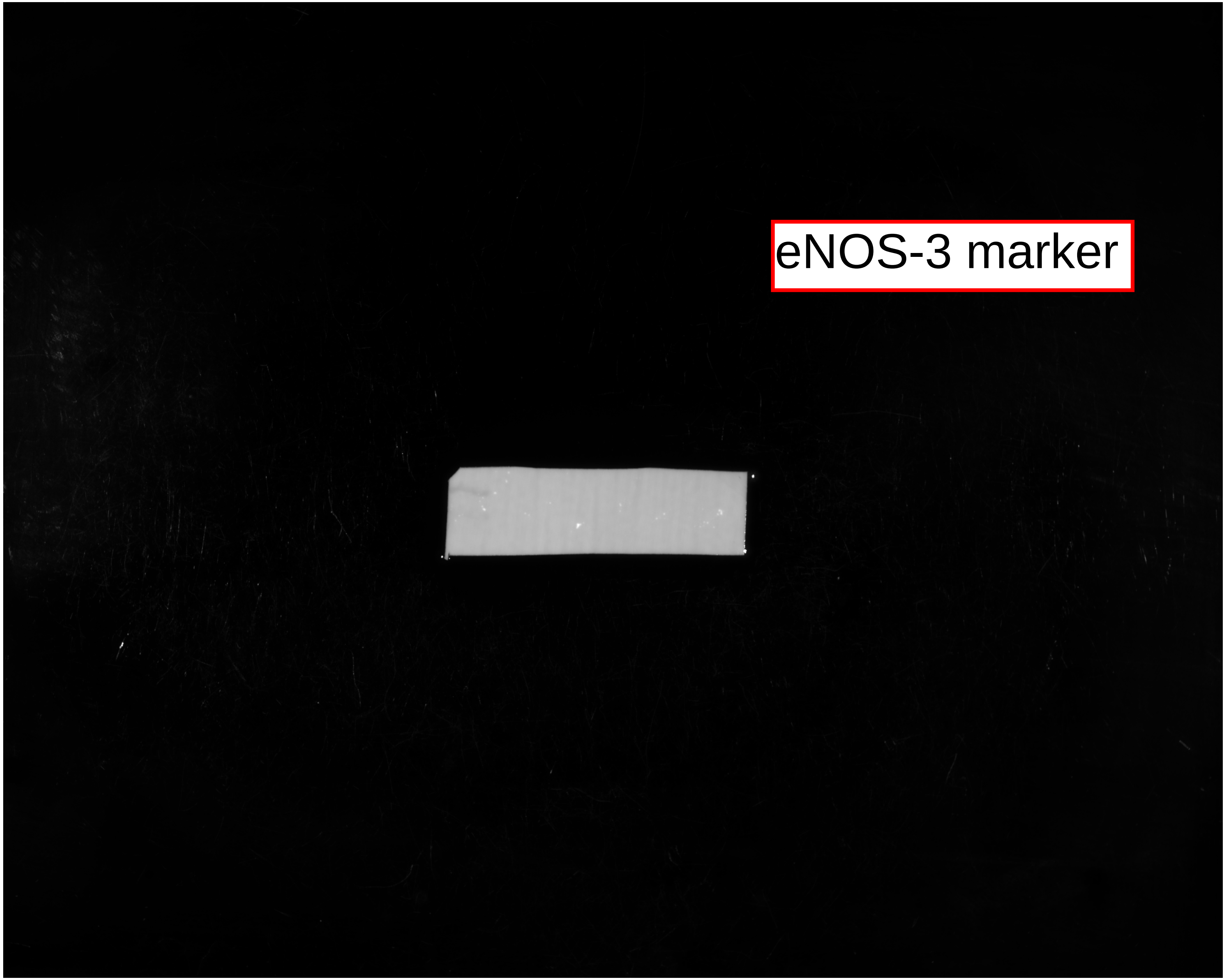
eNOS-3 marker

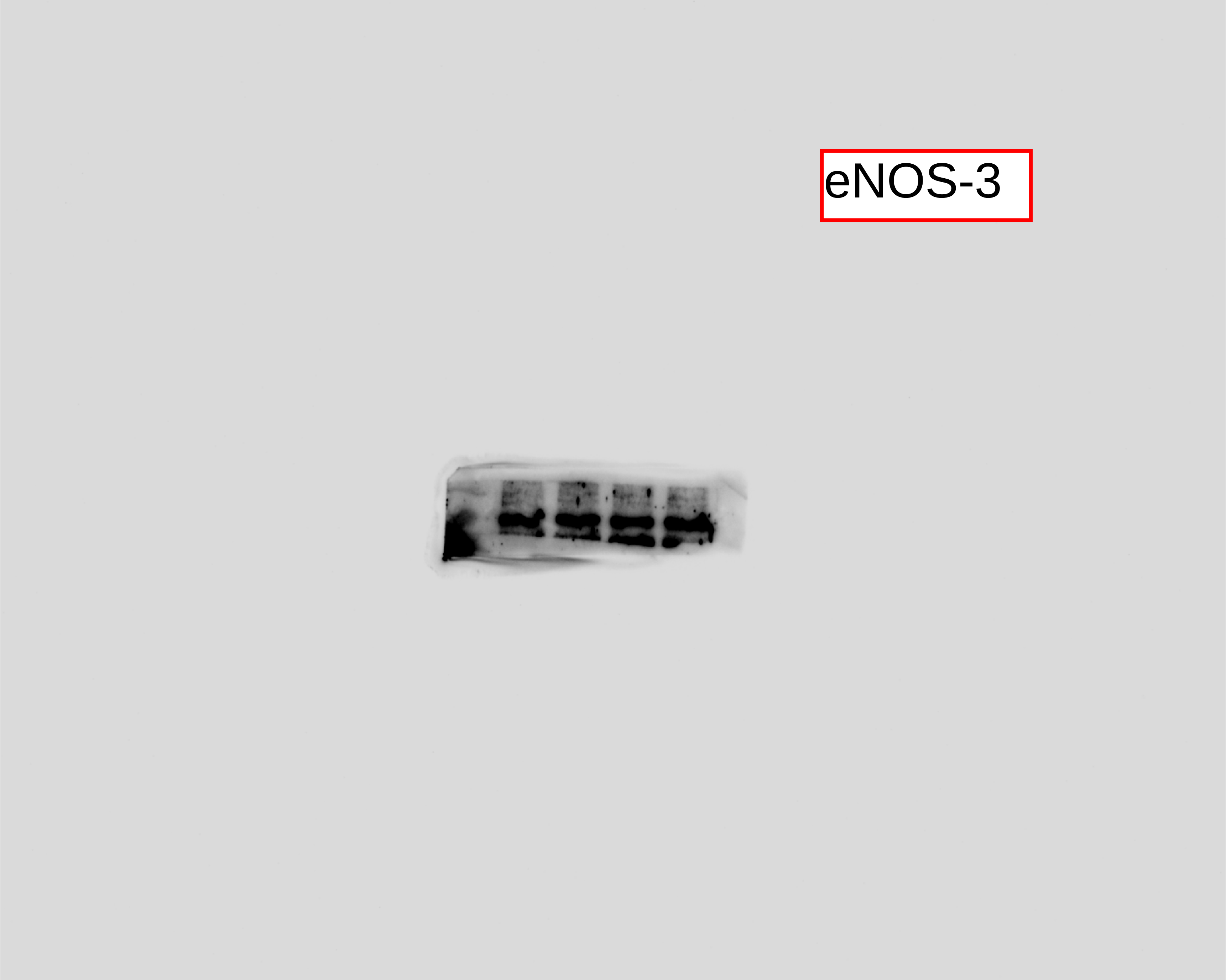
eNOS-3

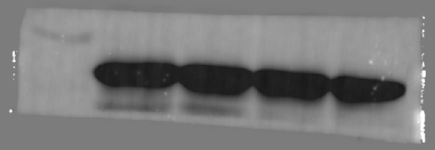

gd (ampk-1-2)
merge

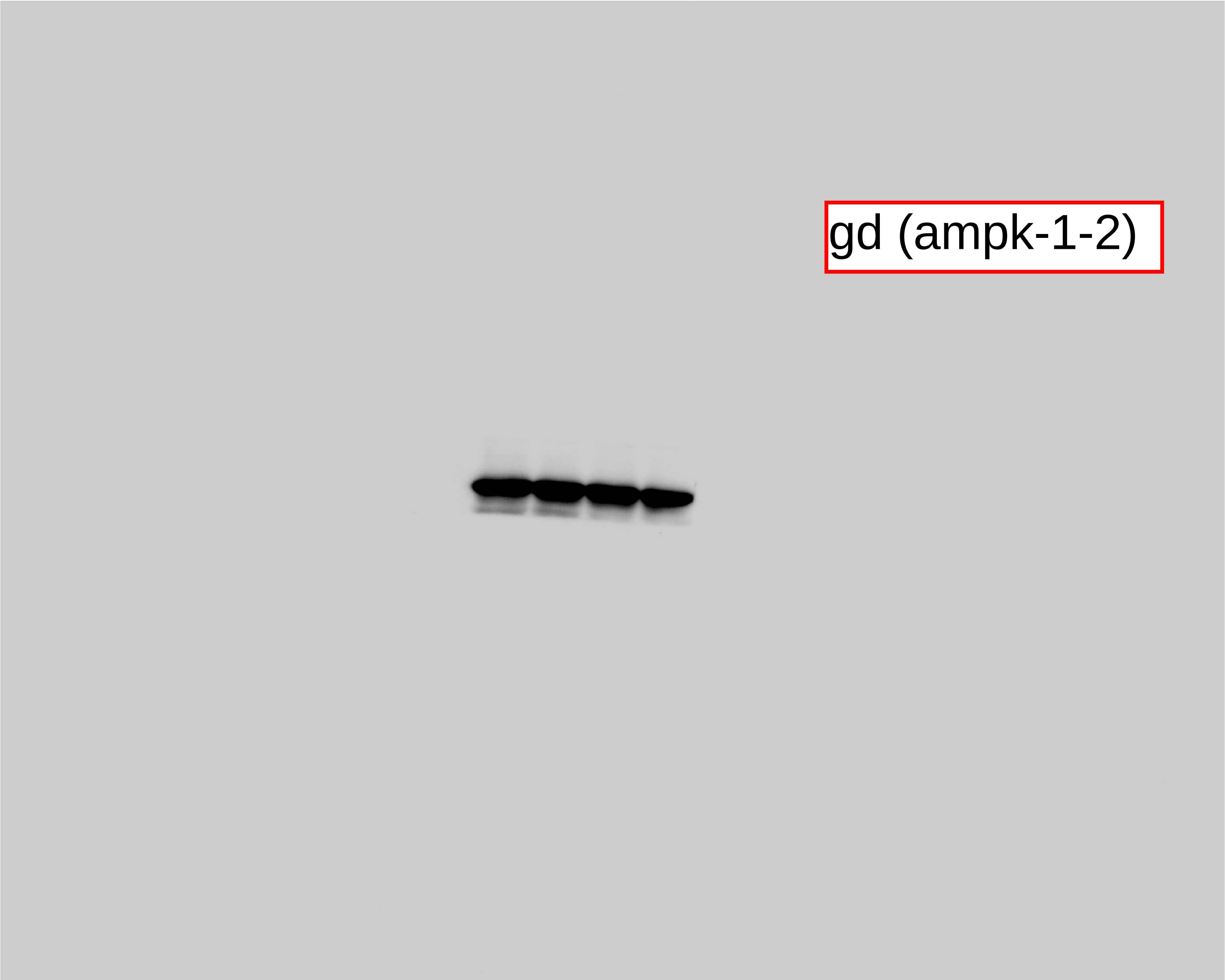
gd (ampk-1-2)

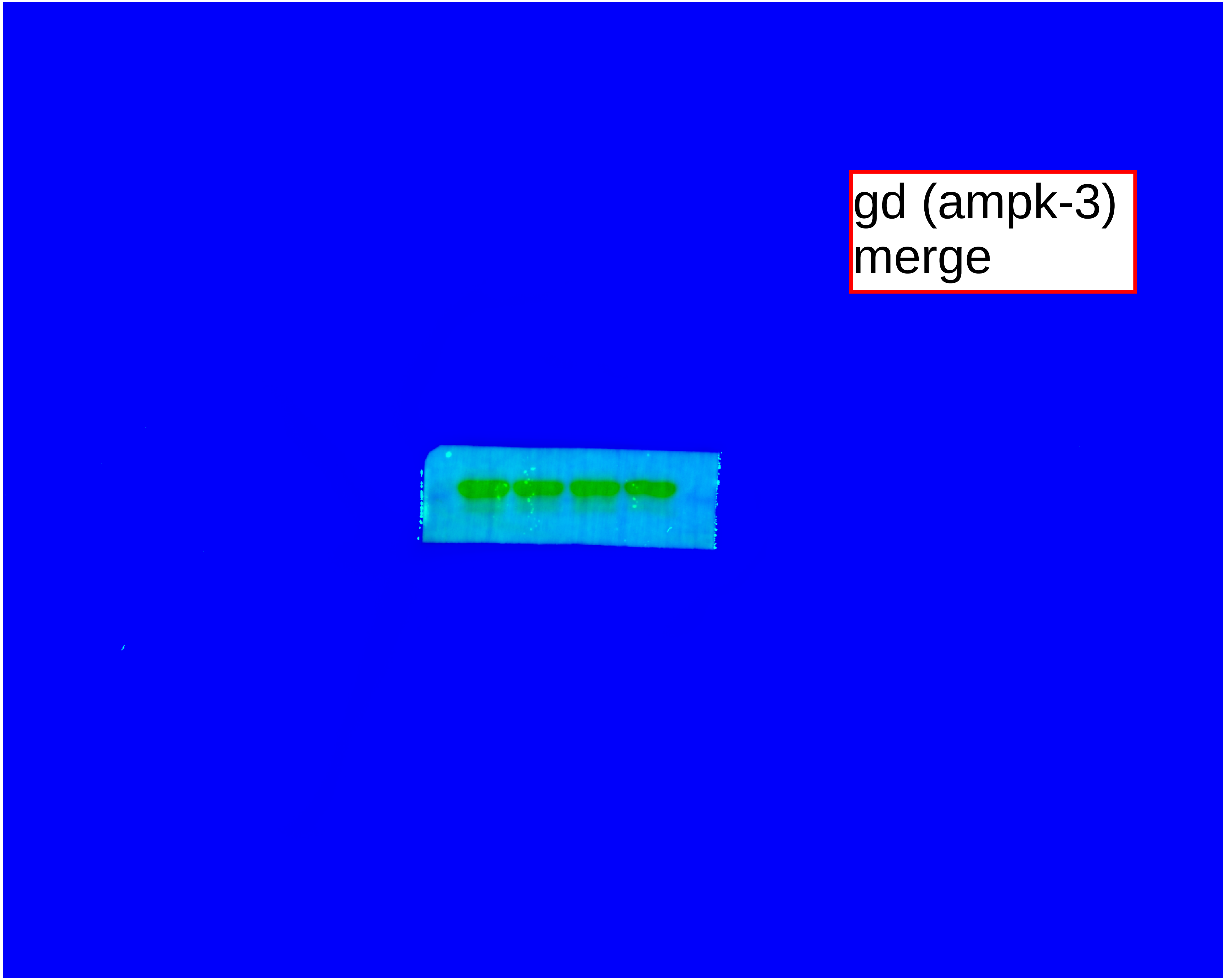
gd (ampk-3)
merge

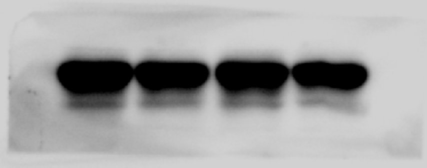

gd (ampk-3)

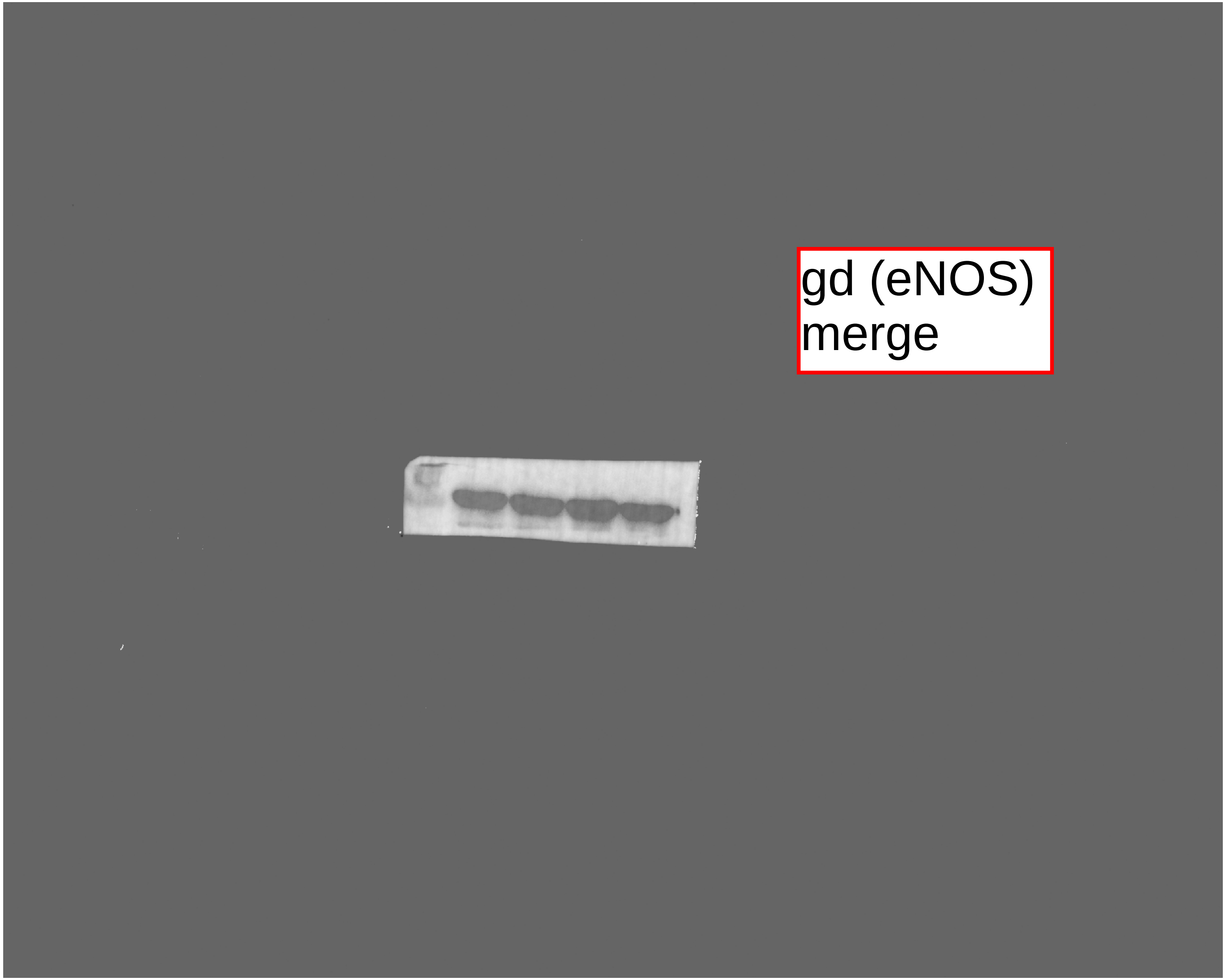

gd (eNOS)
merge

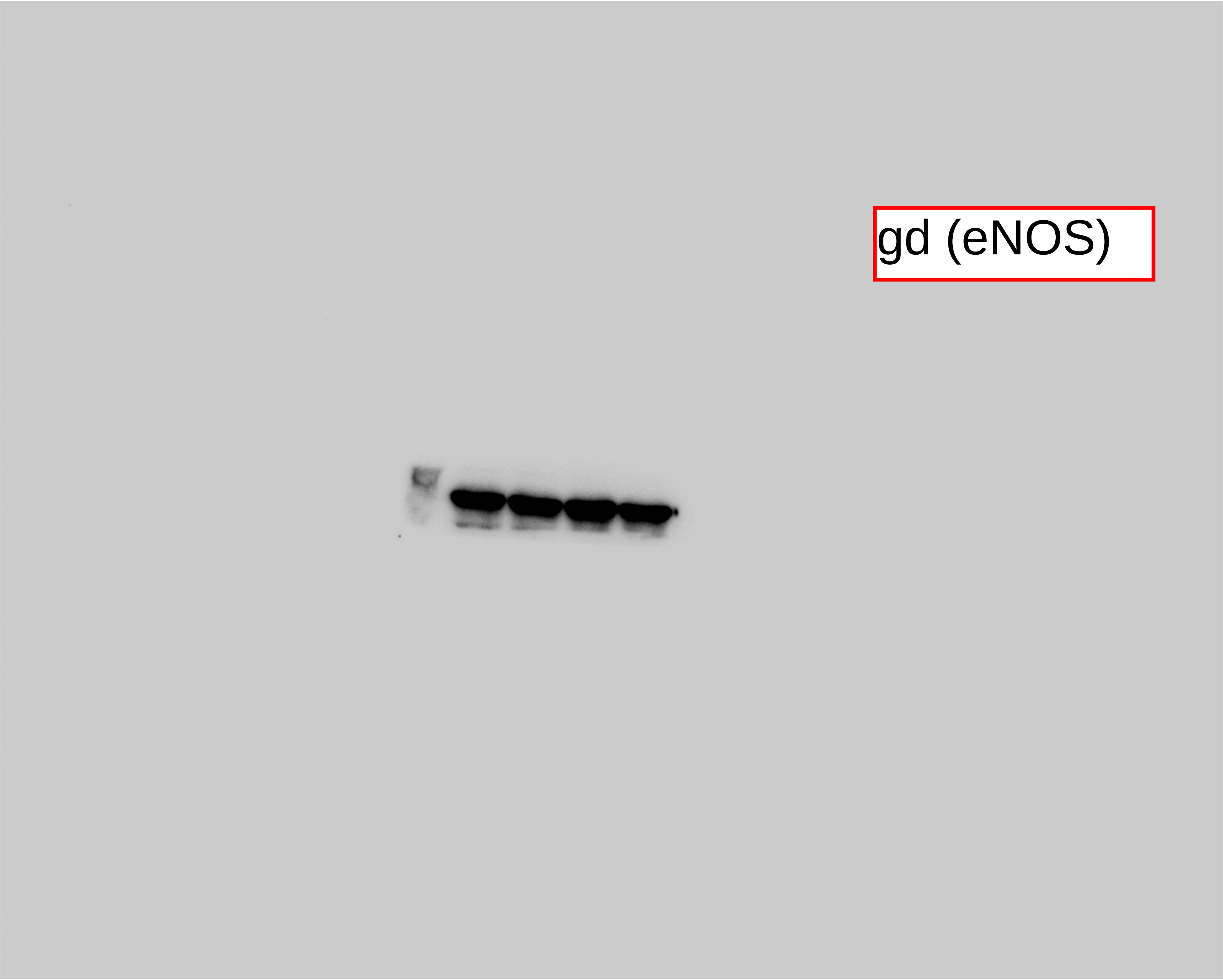
gd (eNOS)

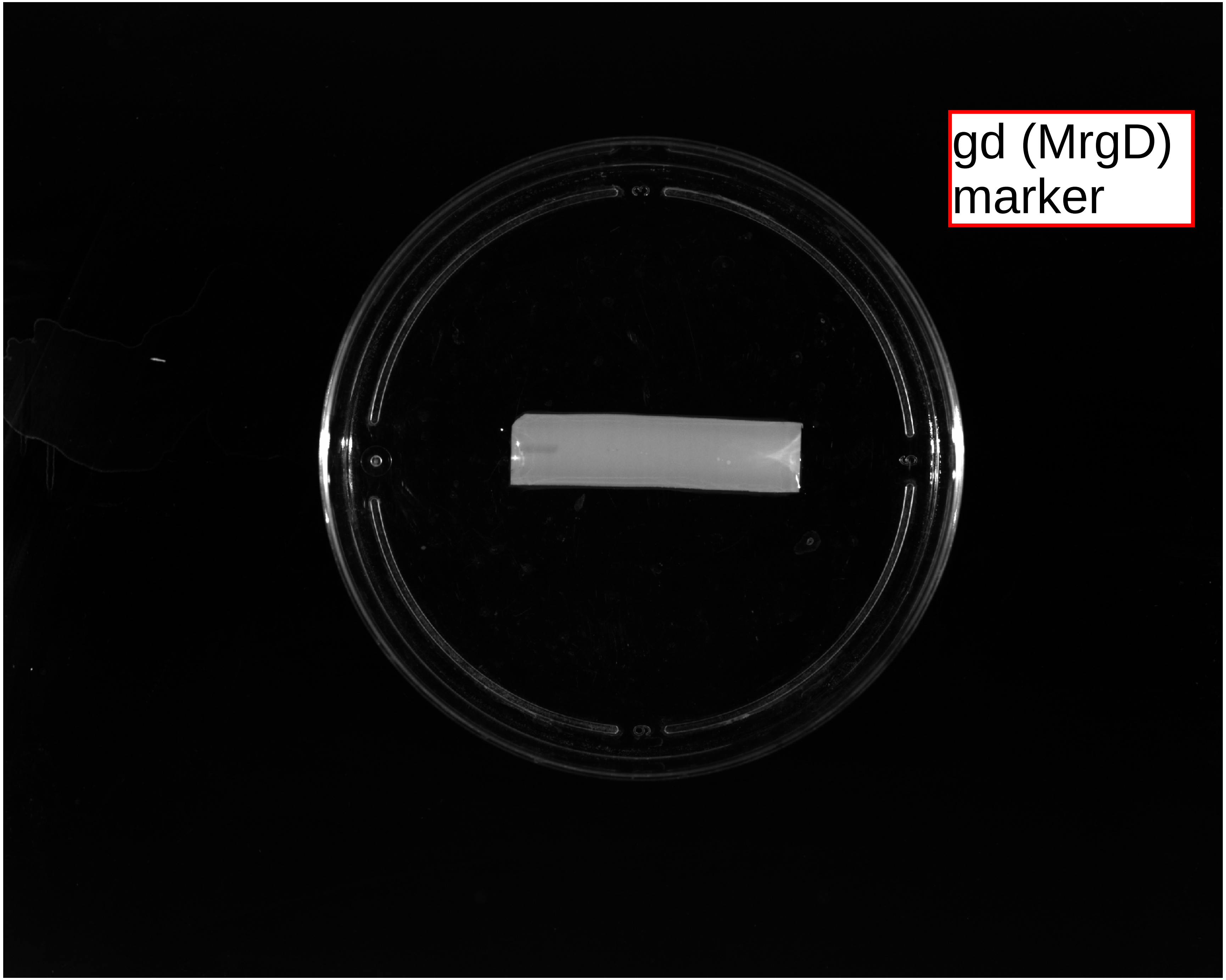
gd (MrgD)
marker

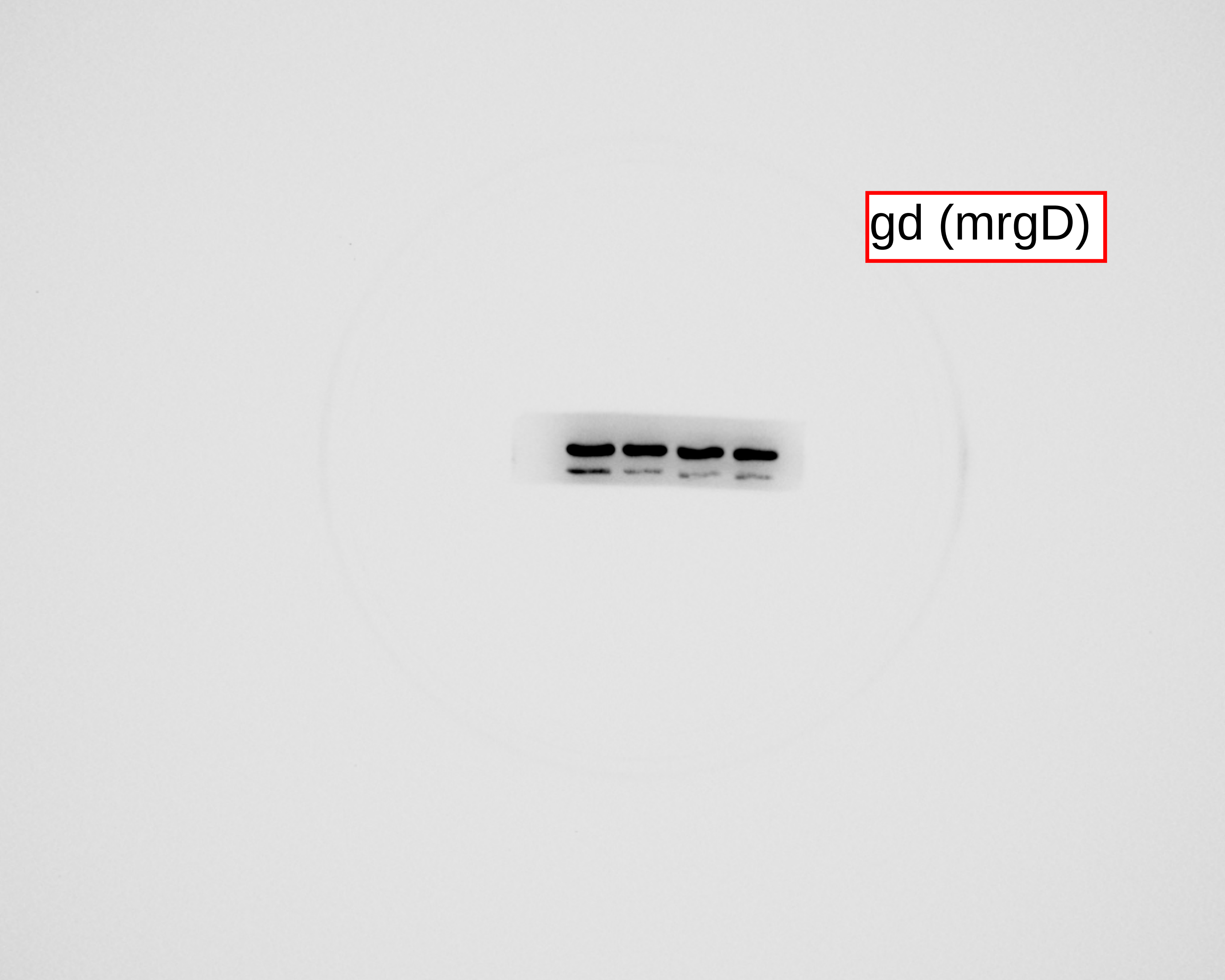
gd (mrgD)

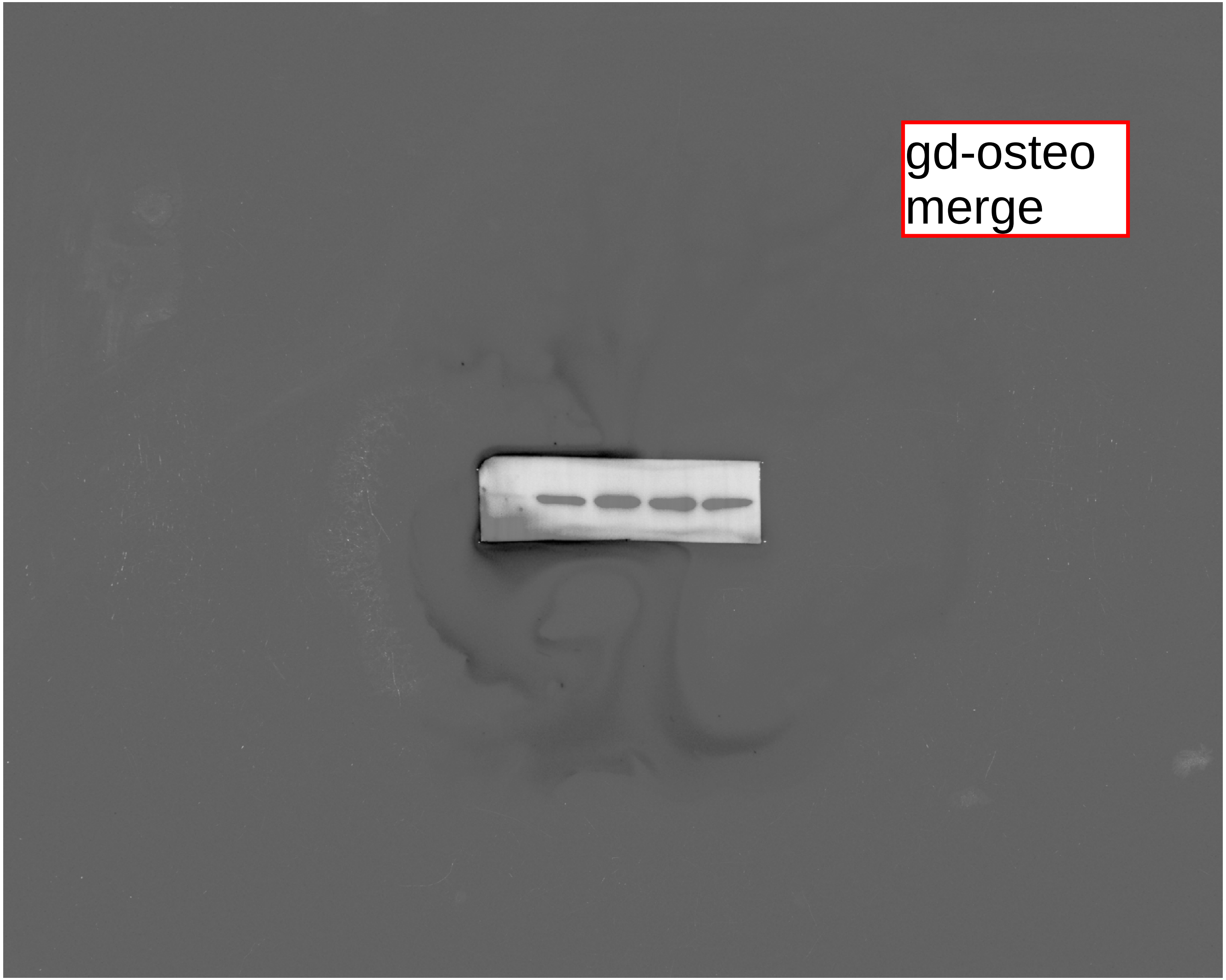
gd-osteo
merge

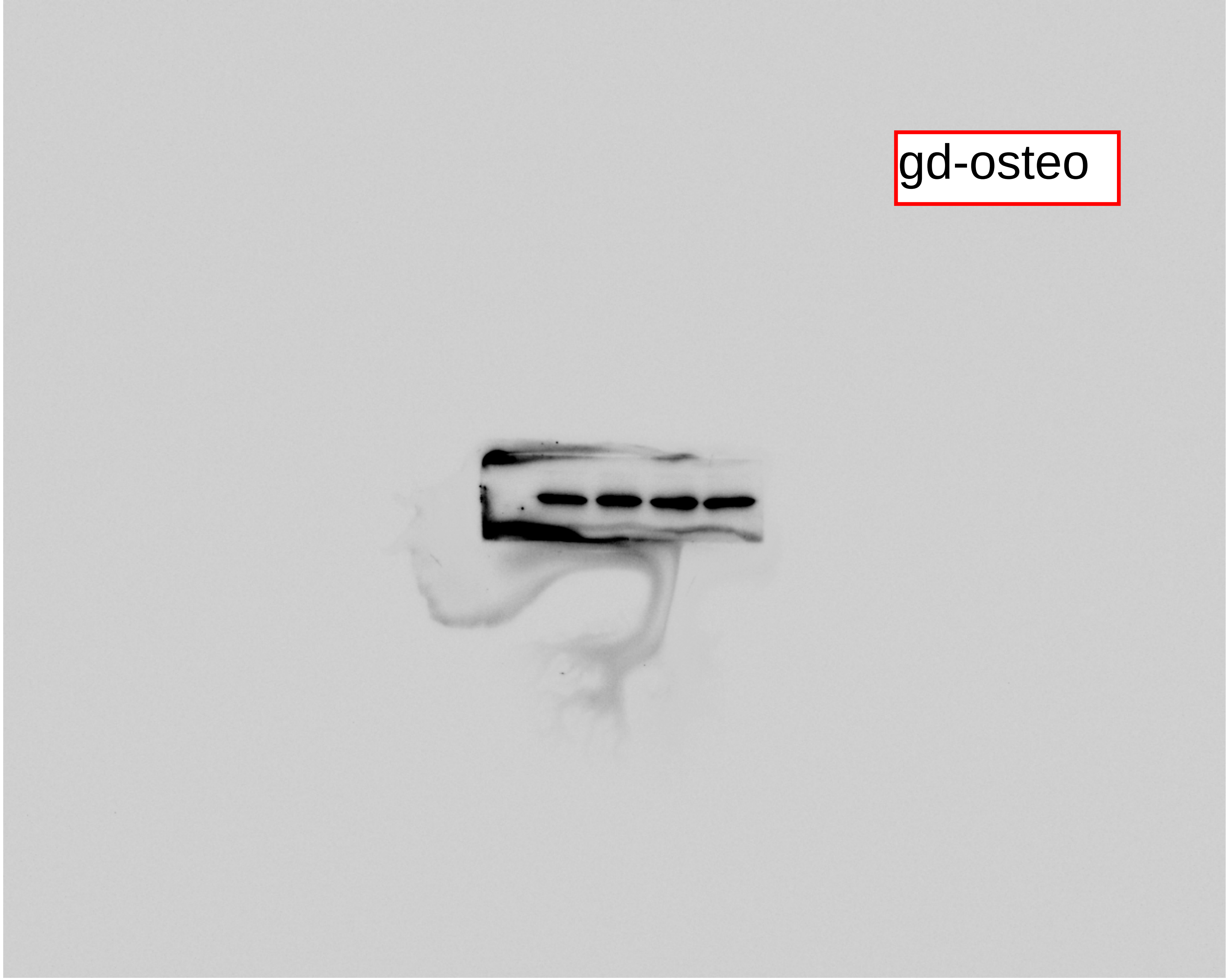

gd-osteo

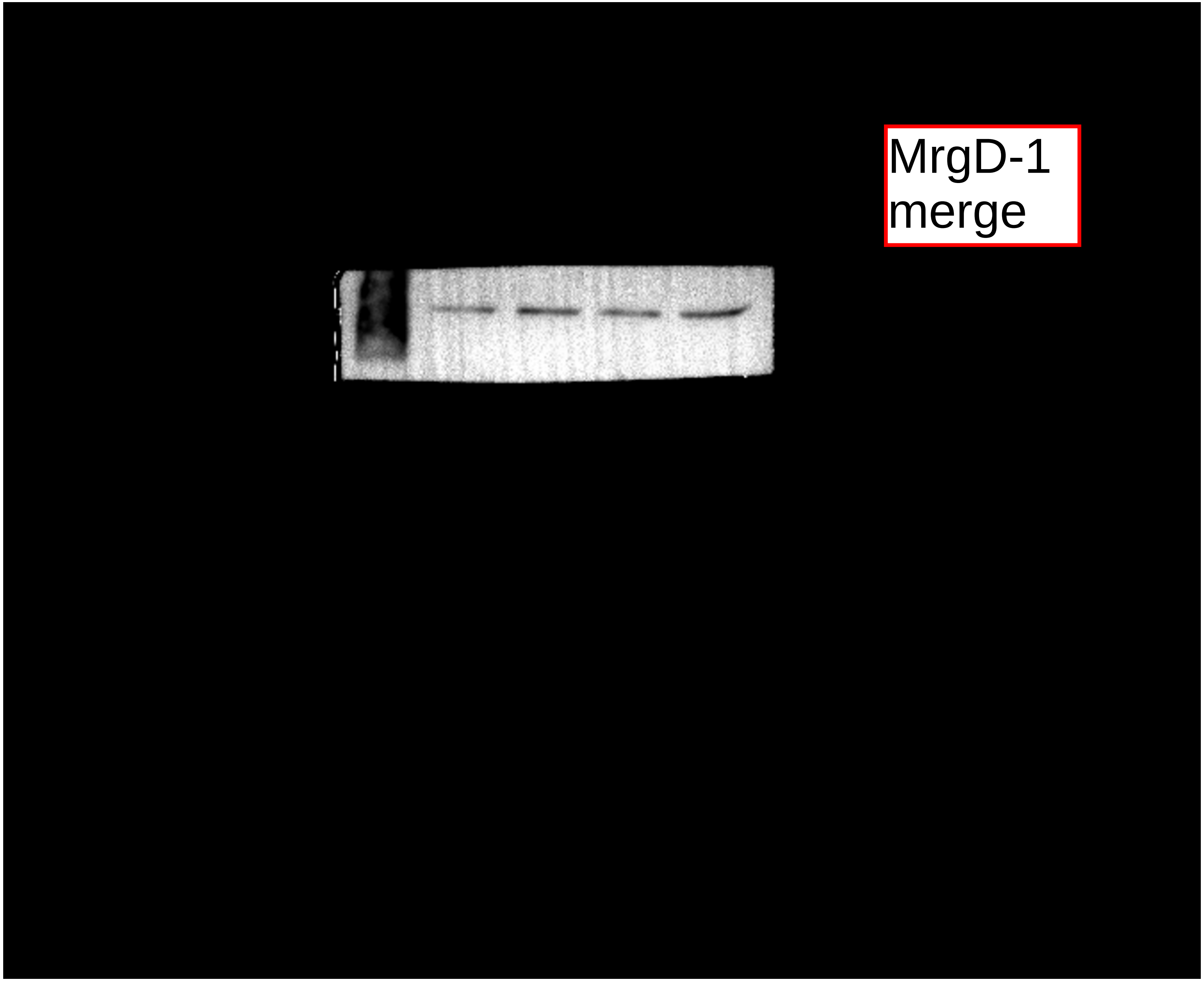
MrgD-1
merge

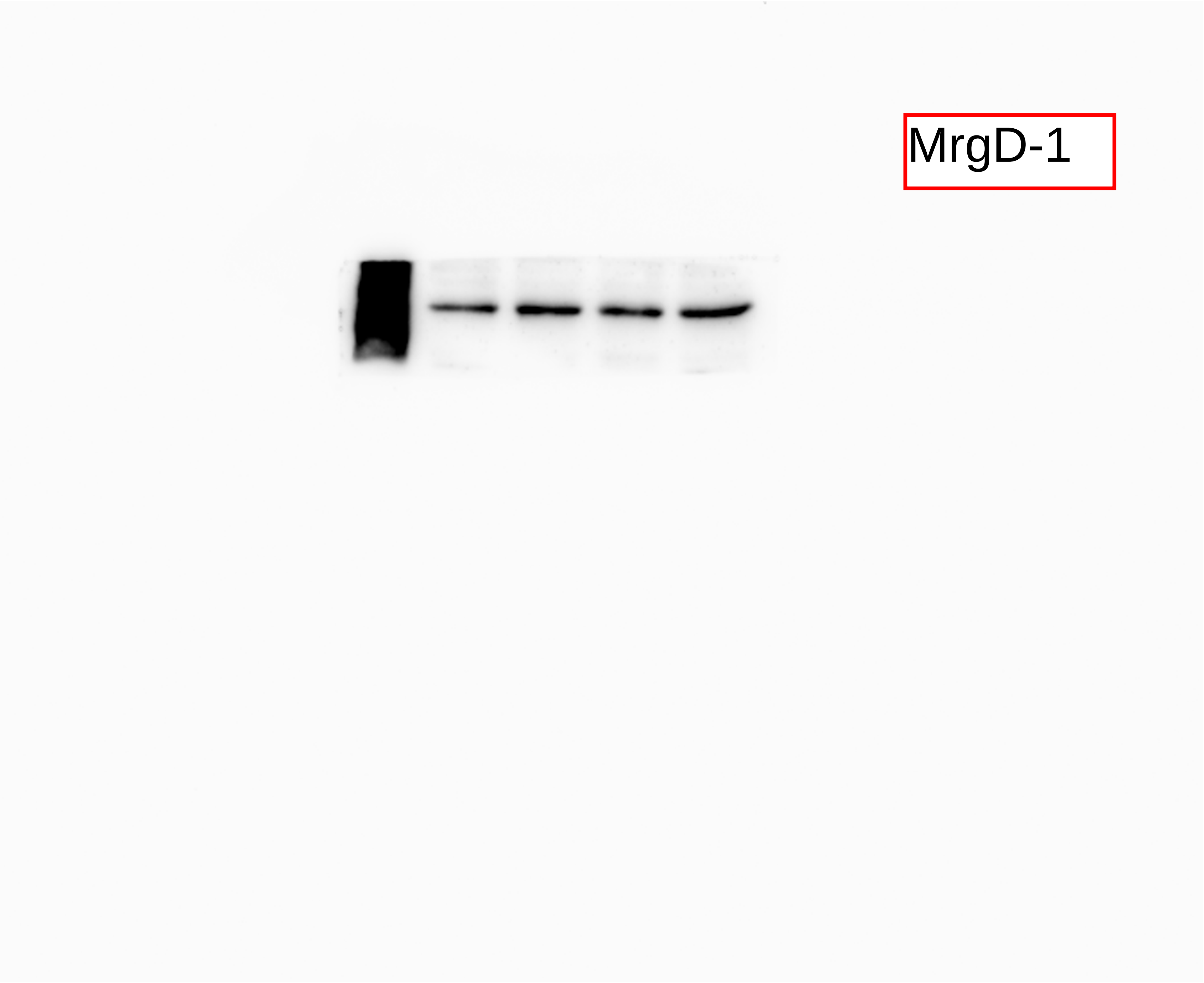
MrgD-1

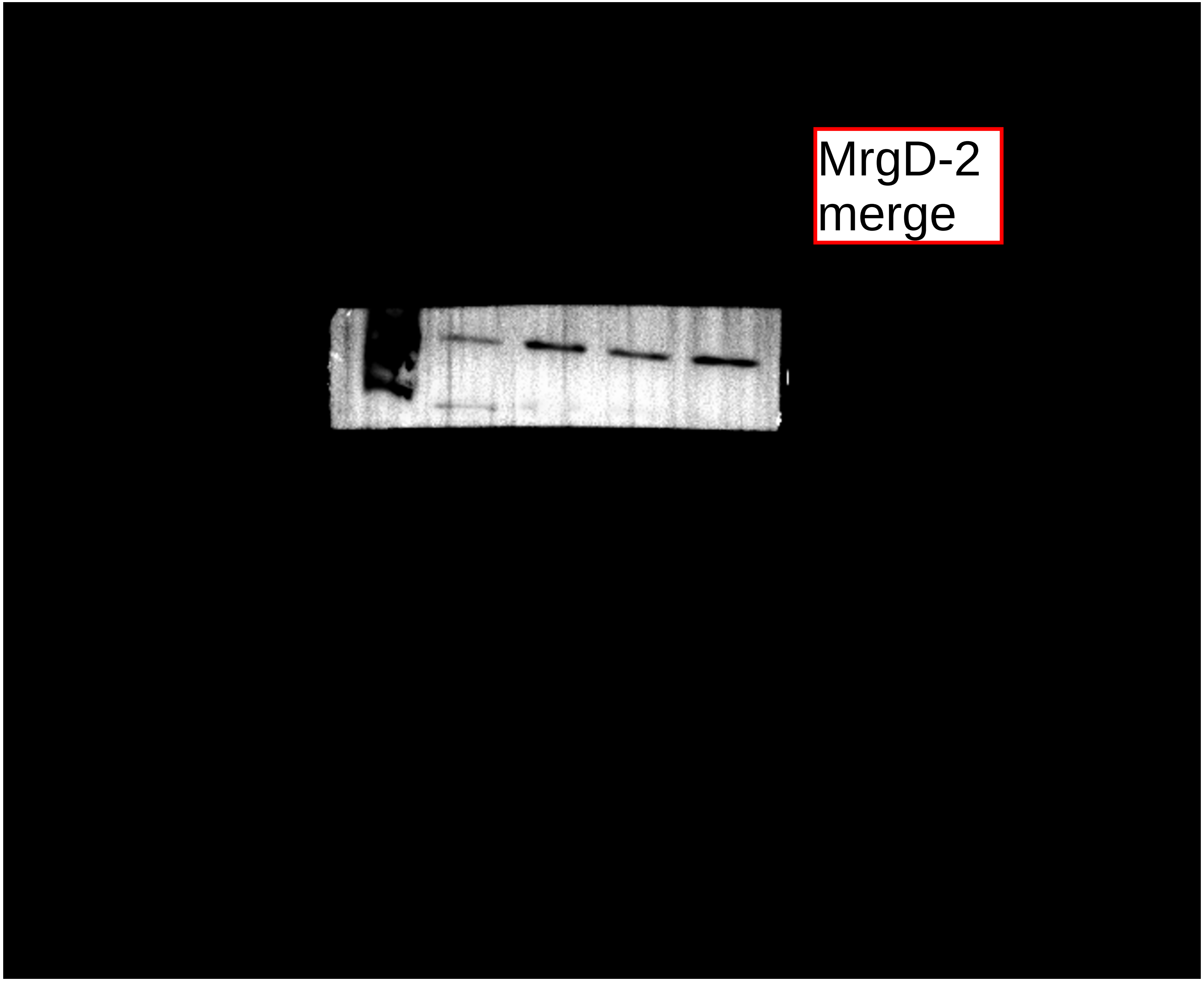
MrgD-2
merge

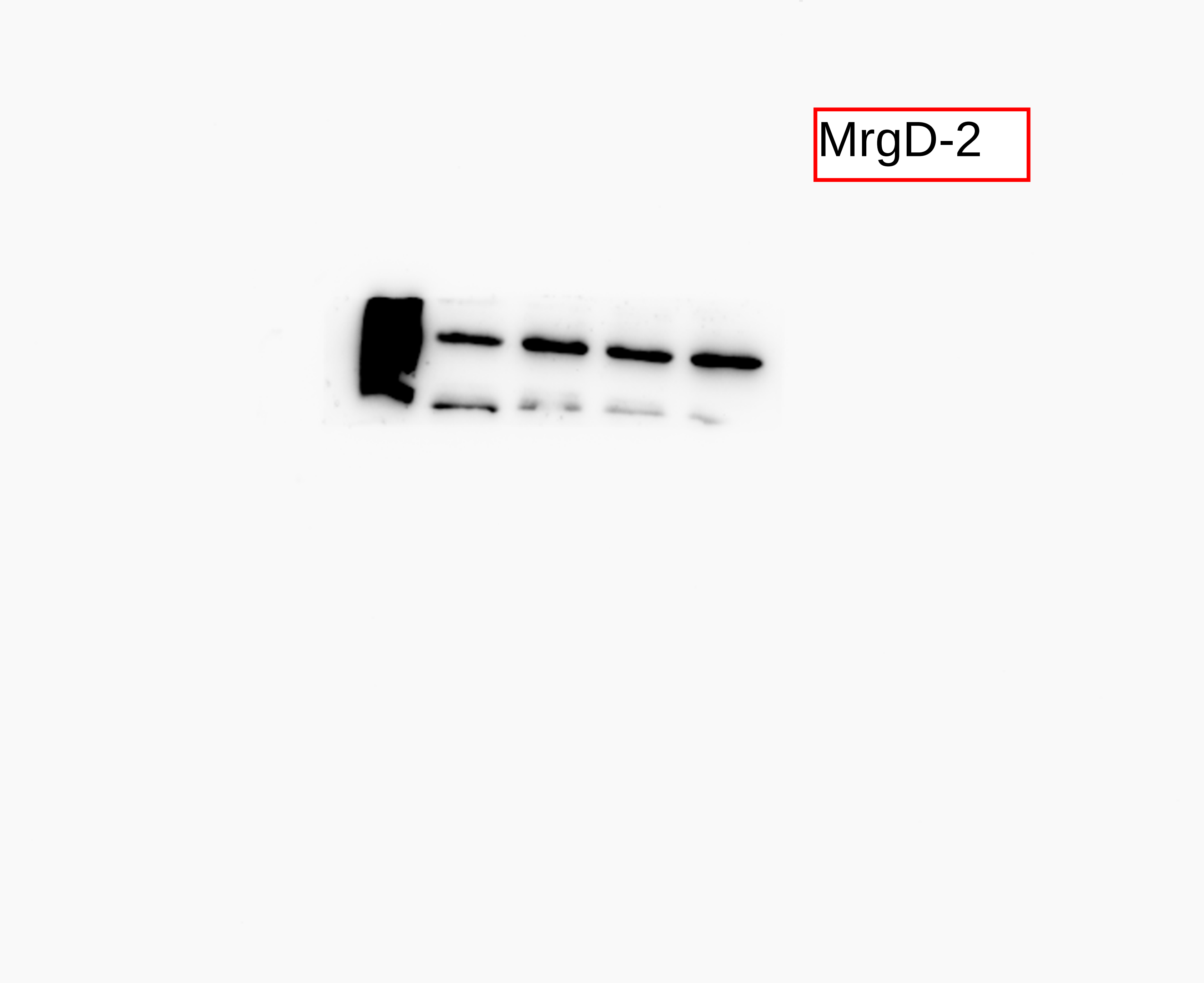
MrgD-2

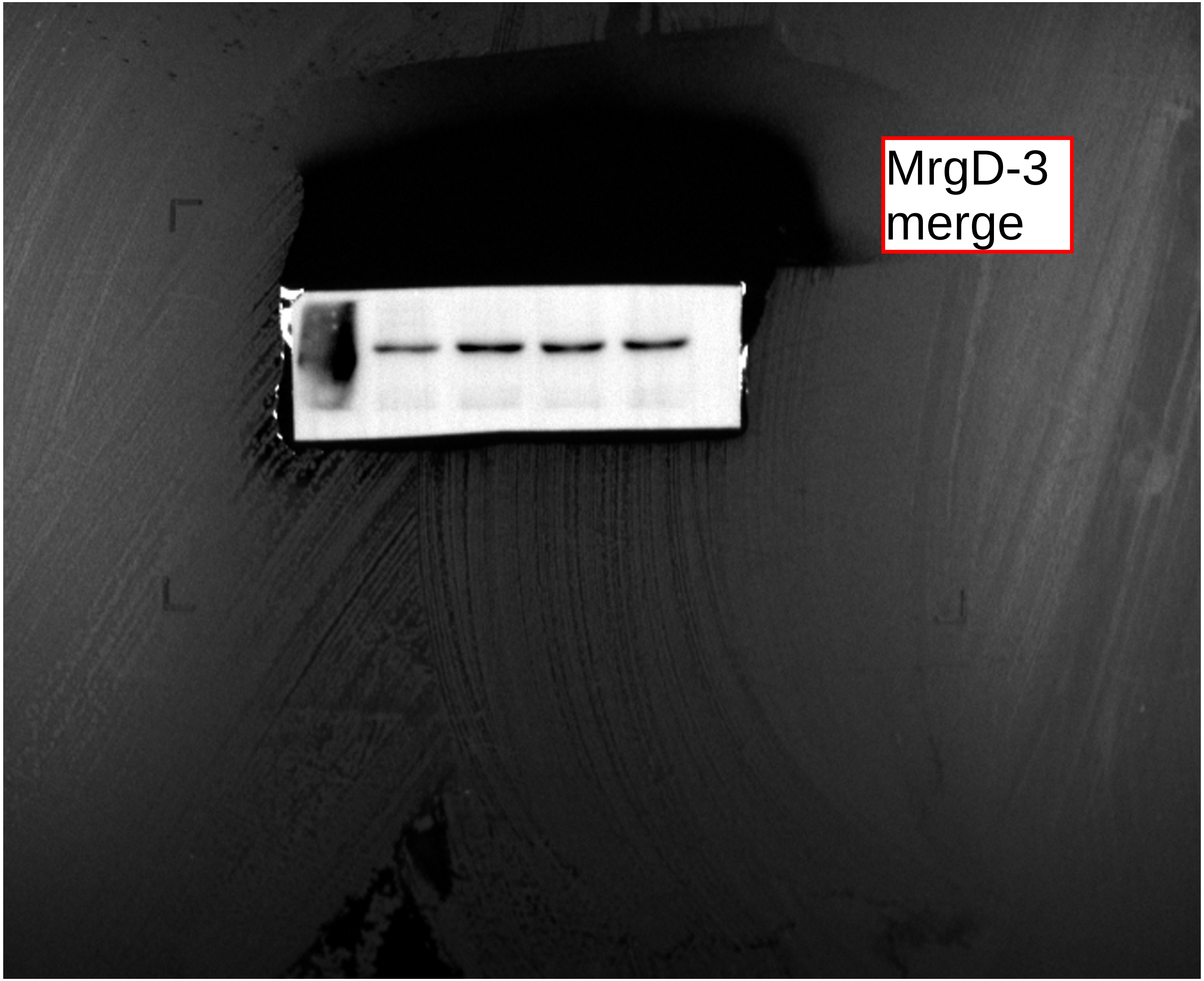
MrgD-3
merge

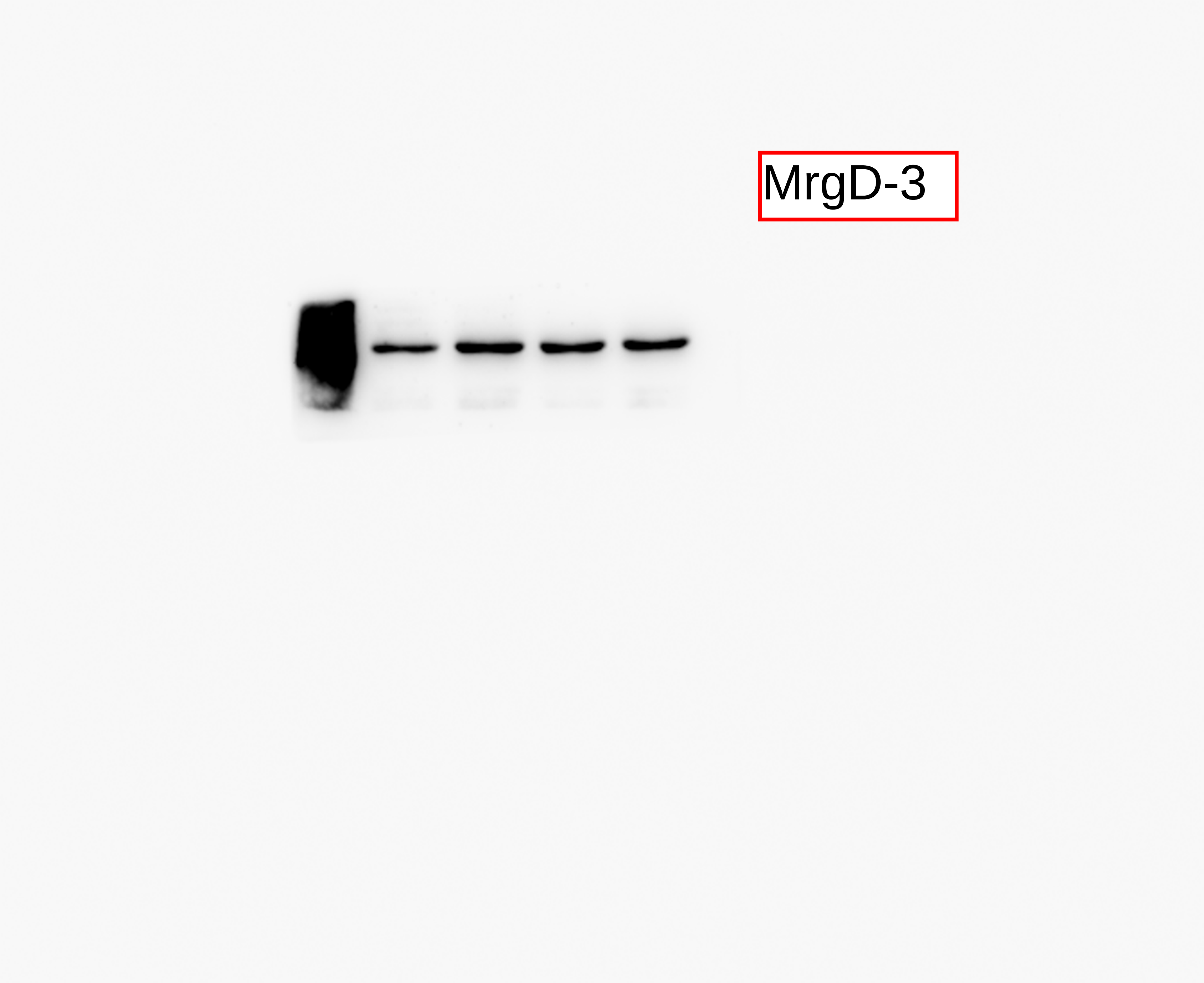
MrgD-3

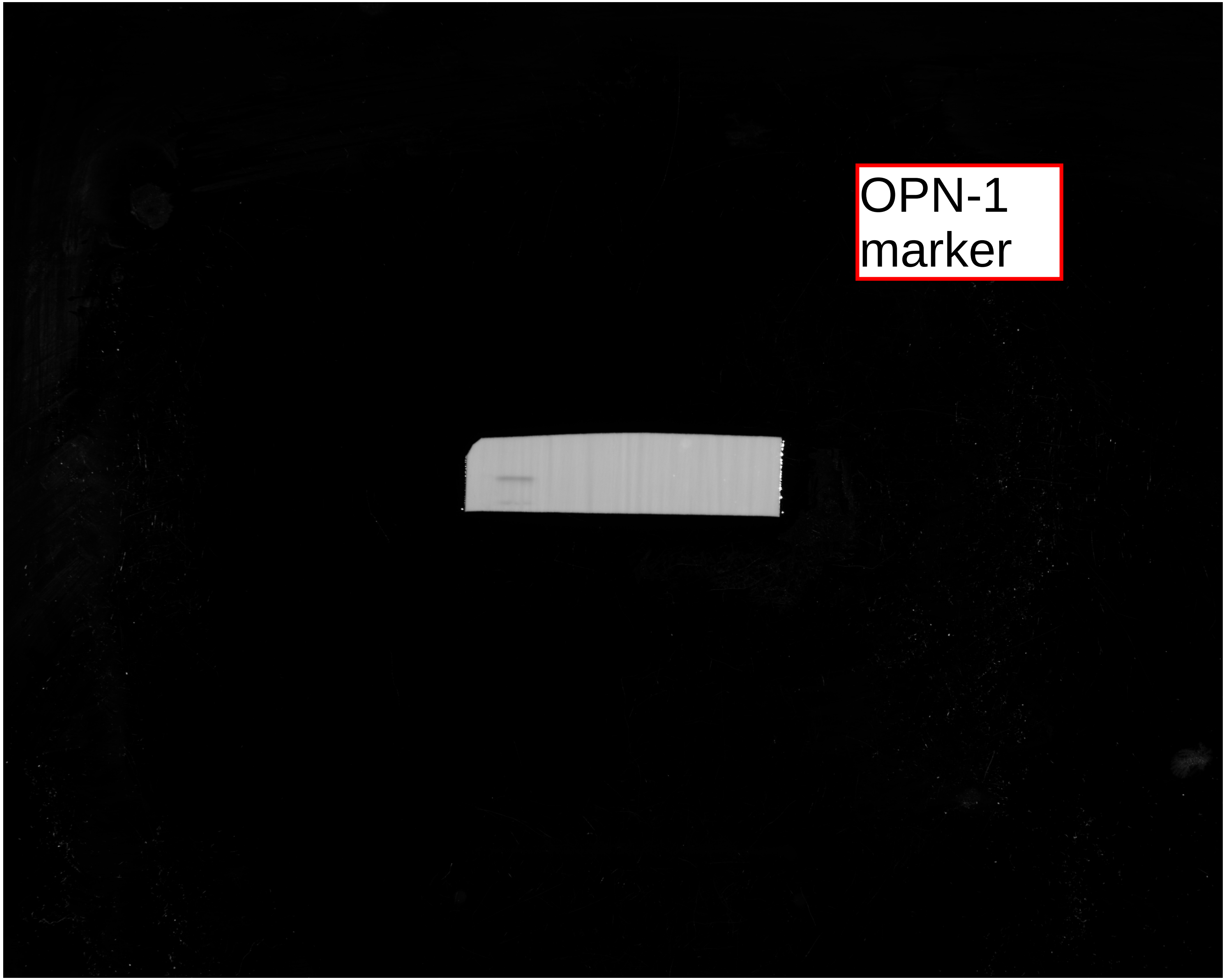
OPN-1
marker

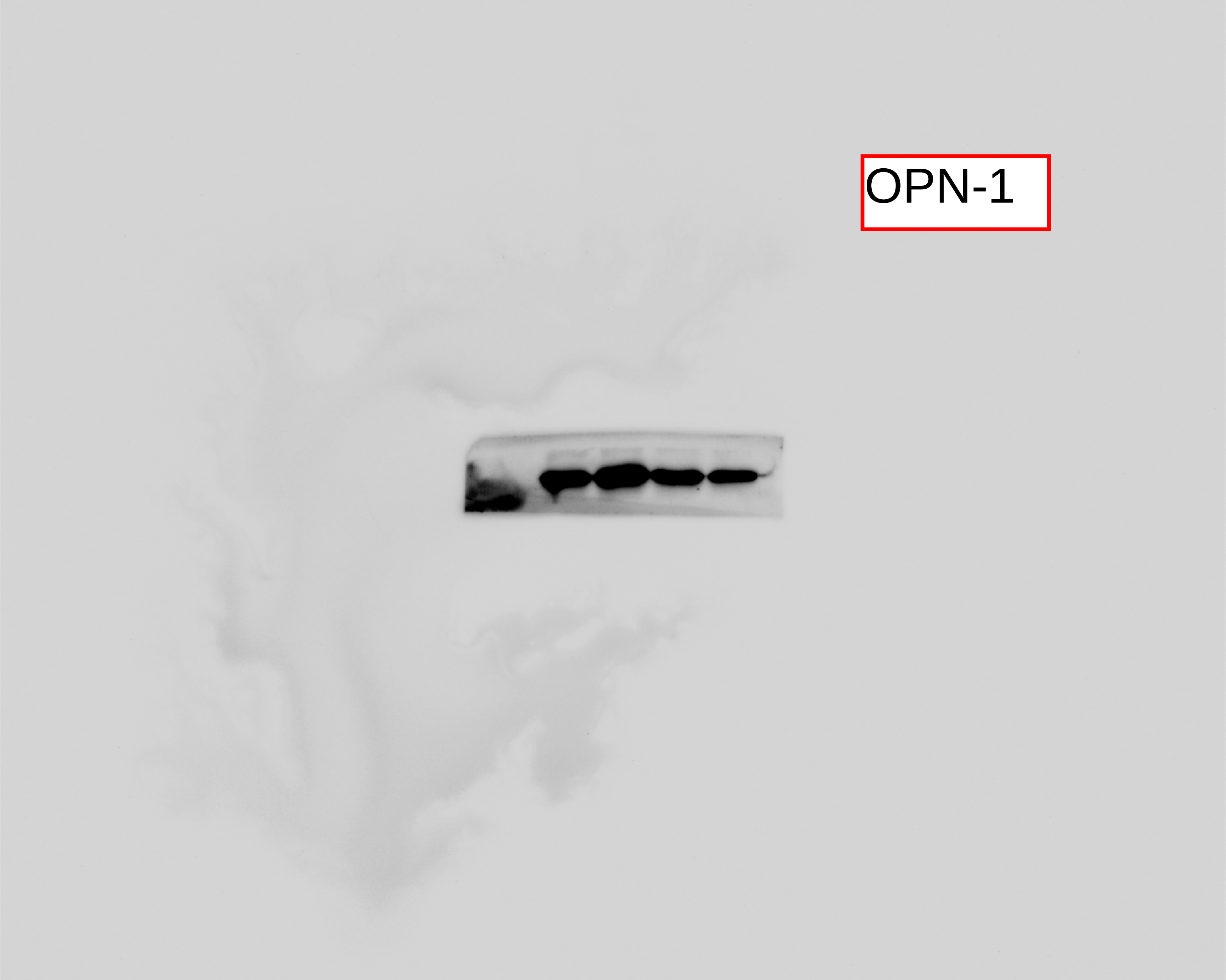
OPN-1

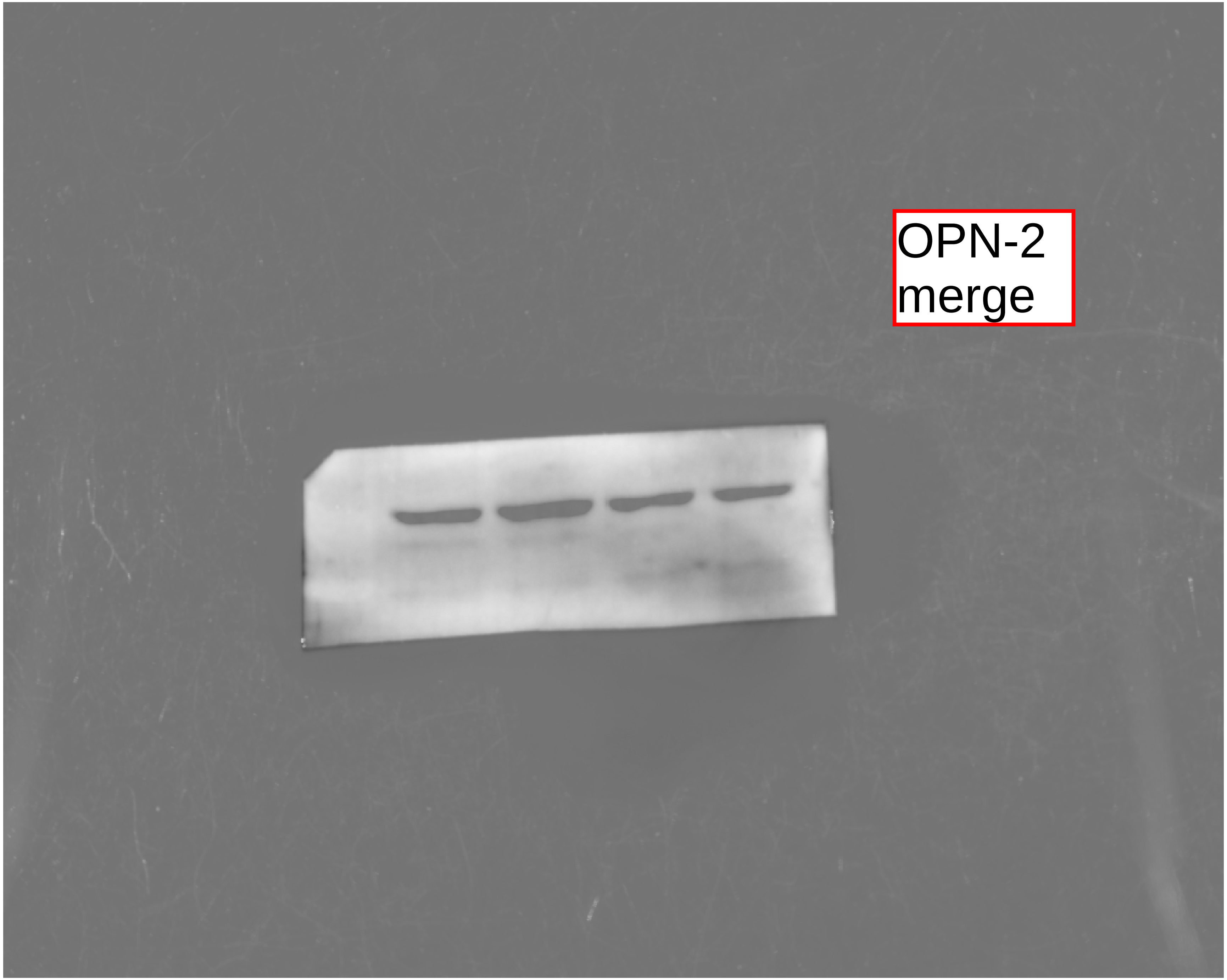
OPN-2
merge

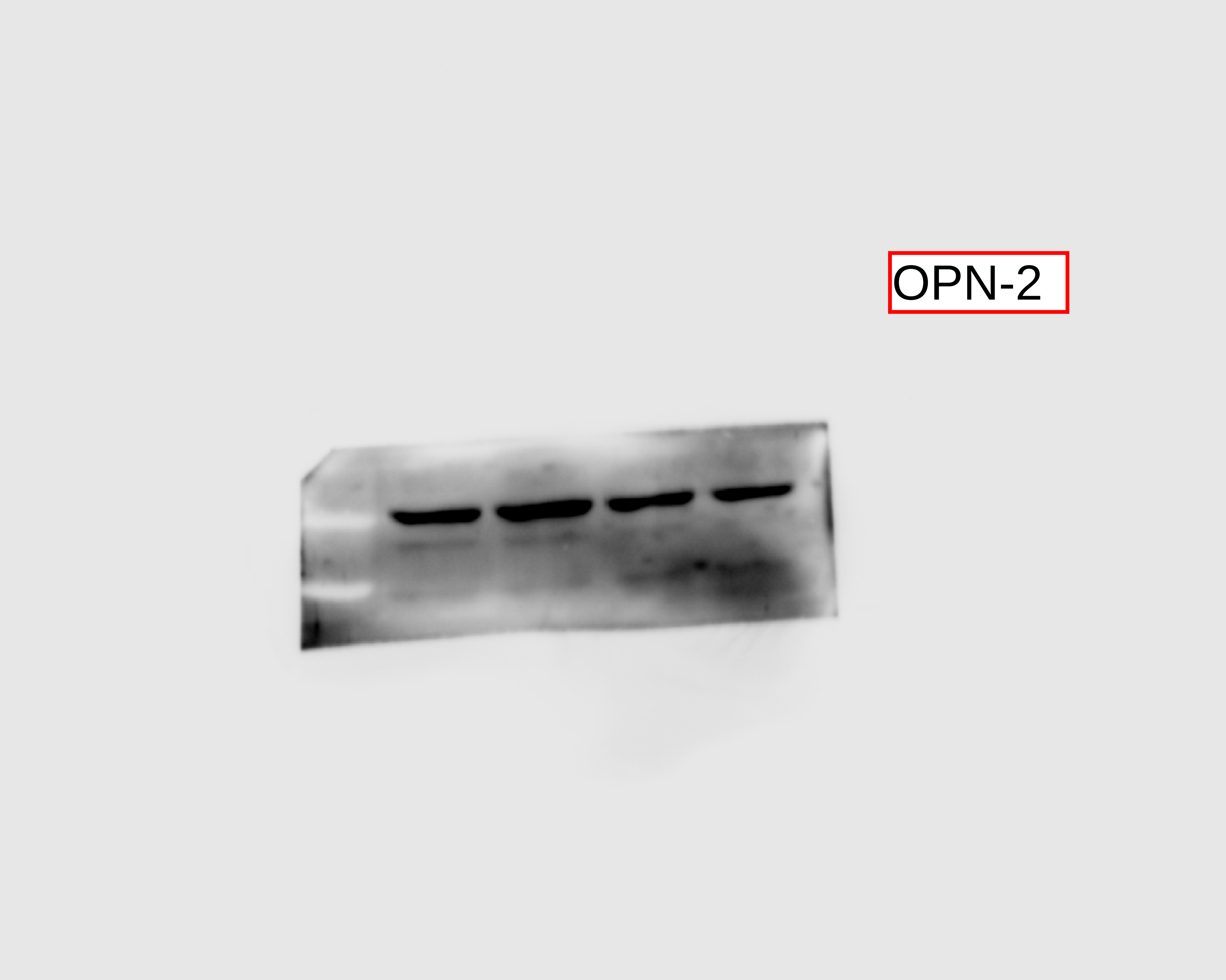
OPN-2

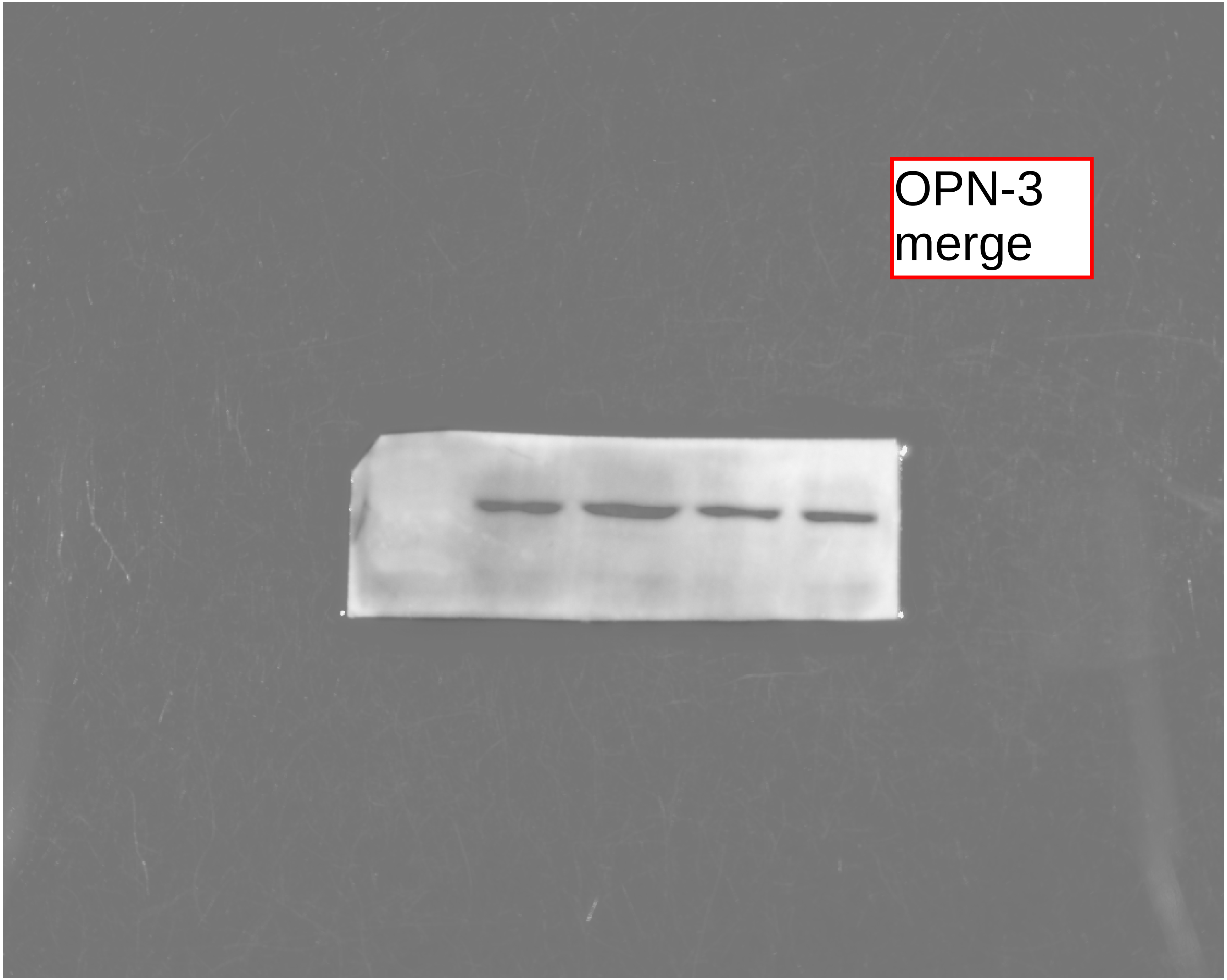
OPN-3
merge

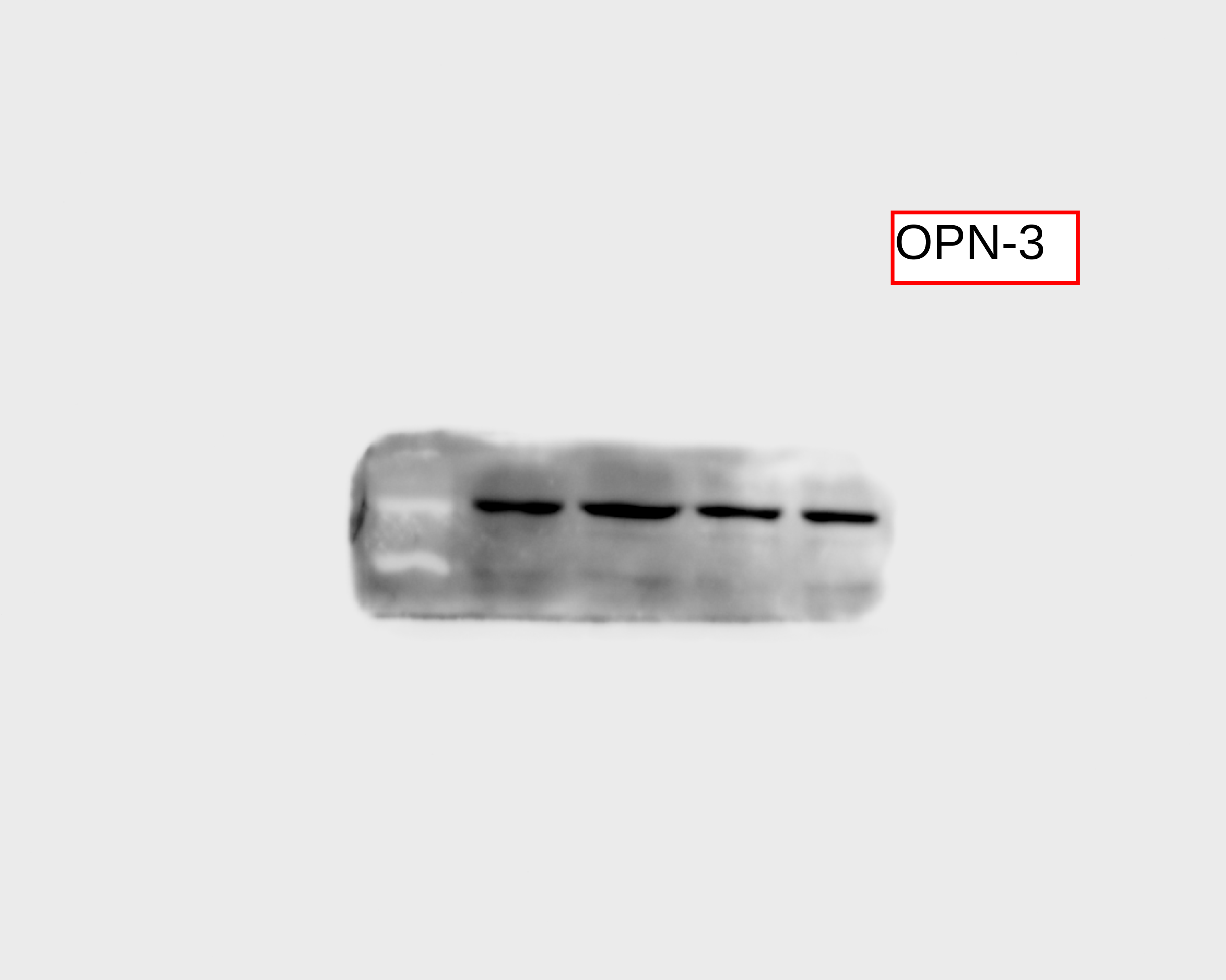
OPN-3

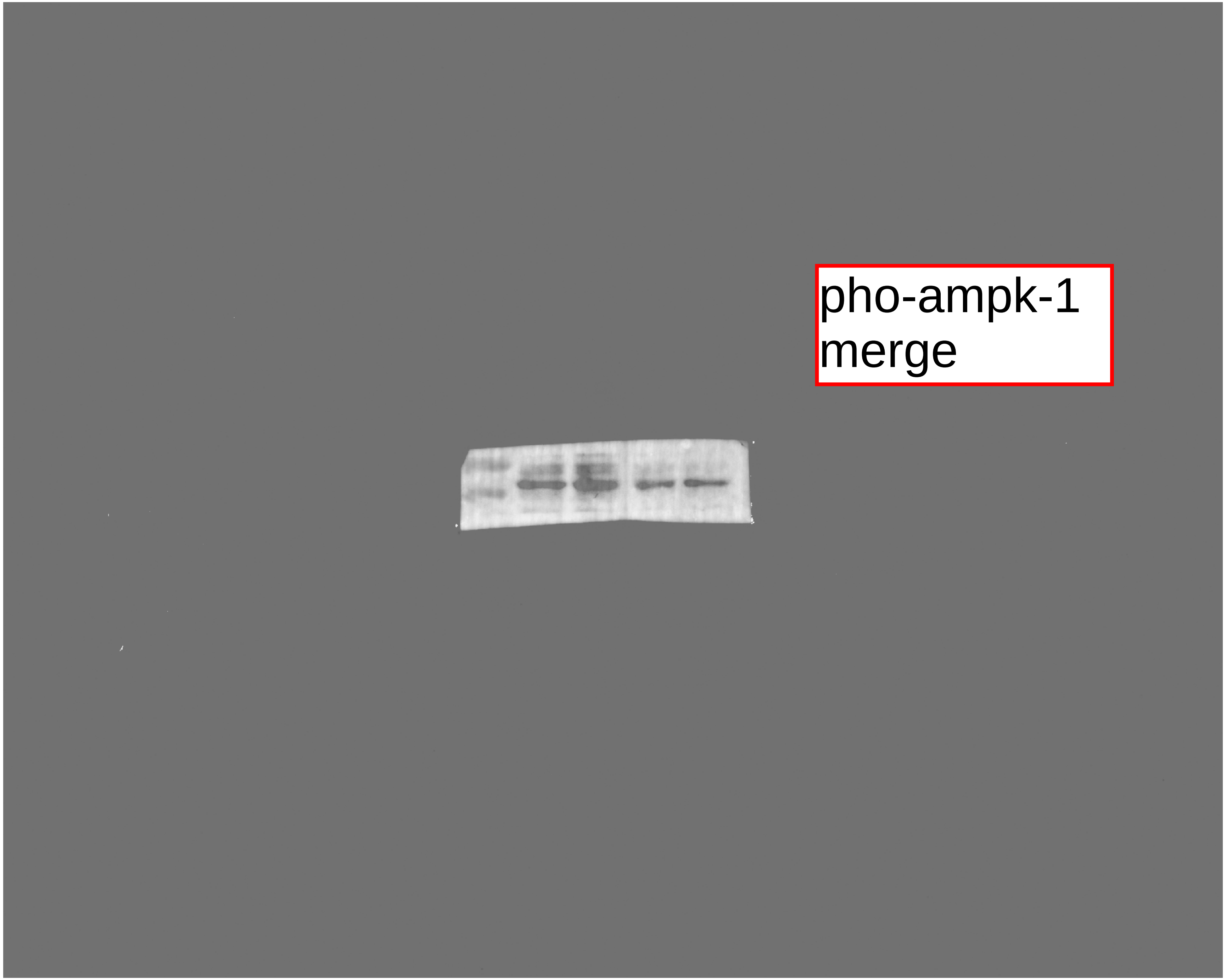
pho-ampk-1
merge

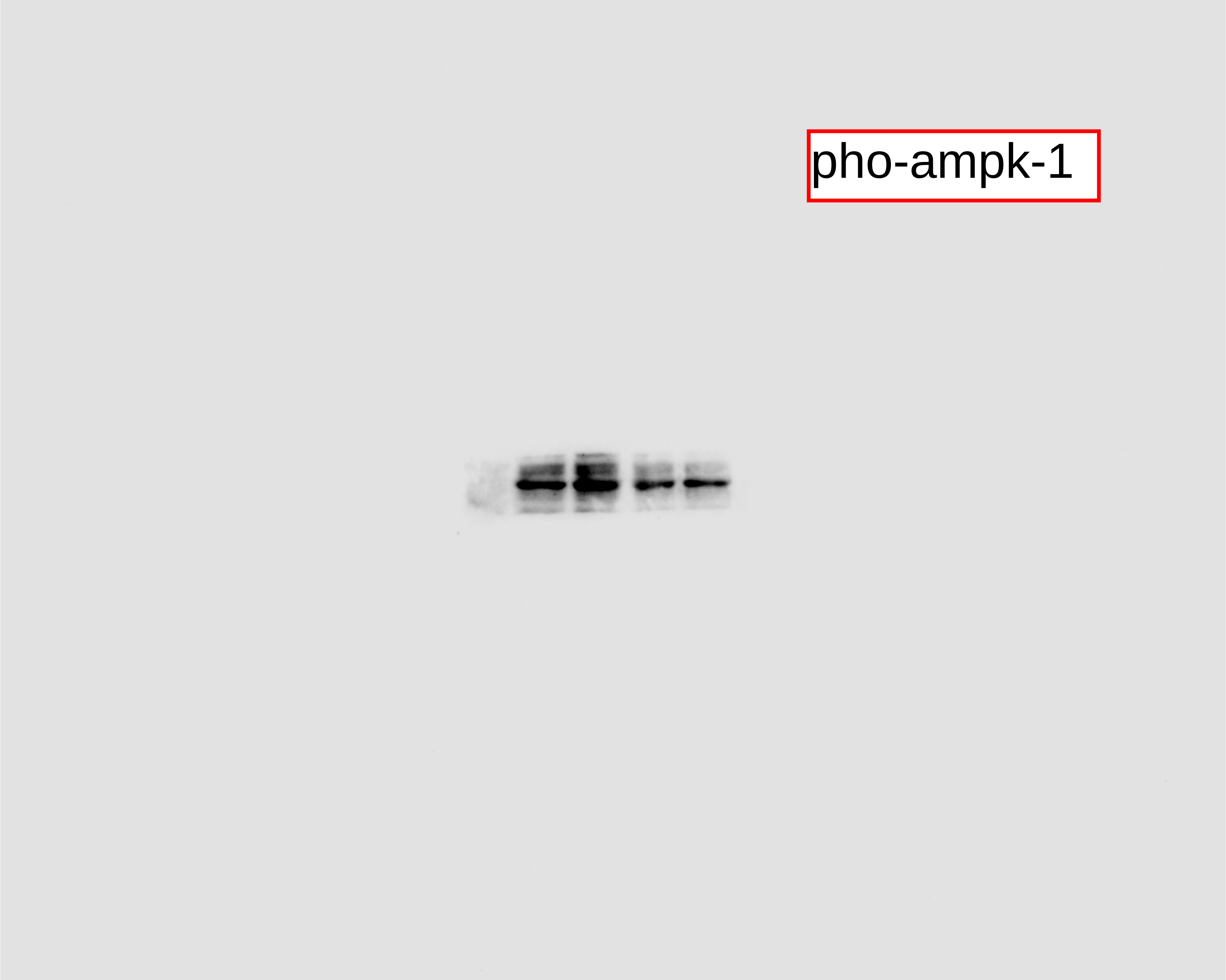
pho-ampk-1

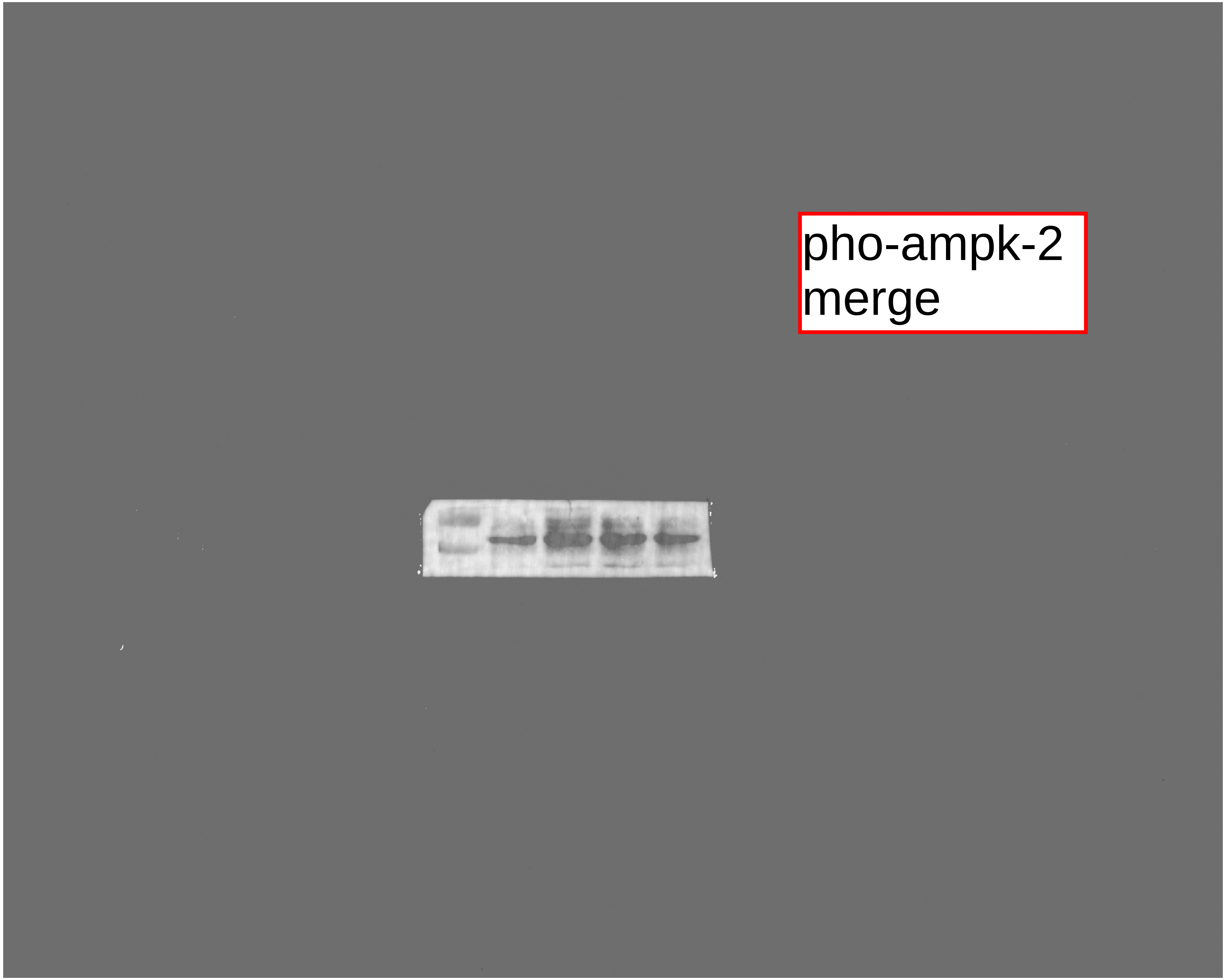
pho-ampk-2
merge

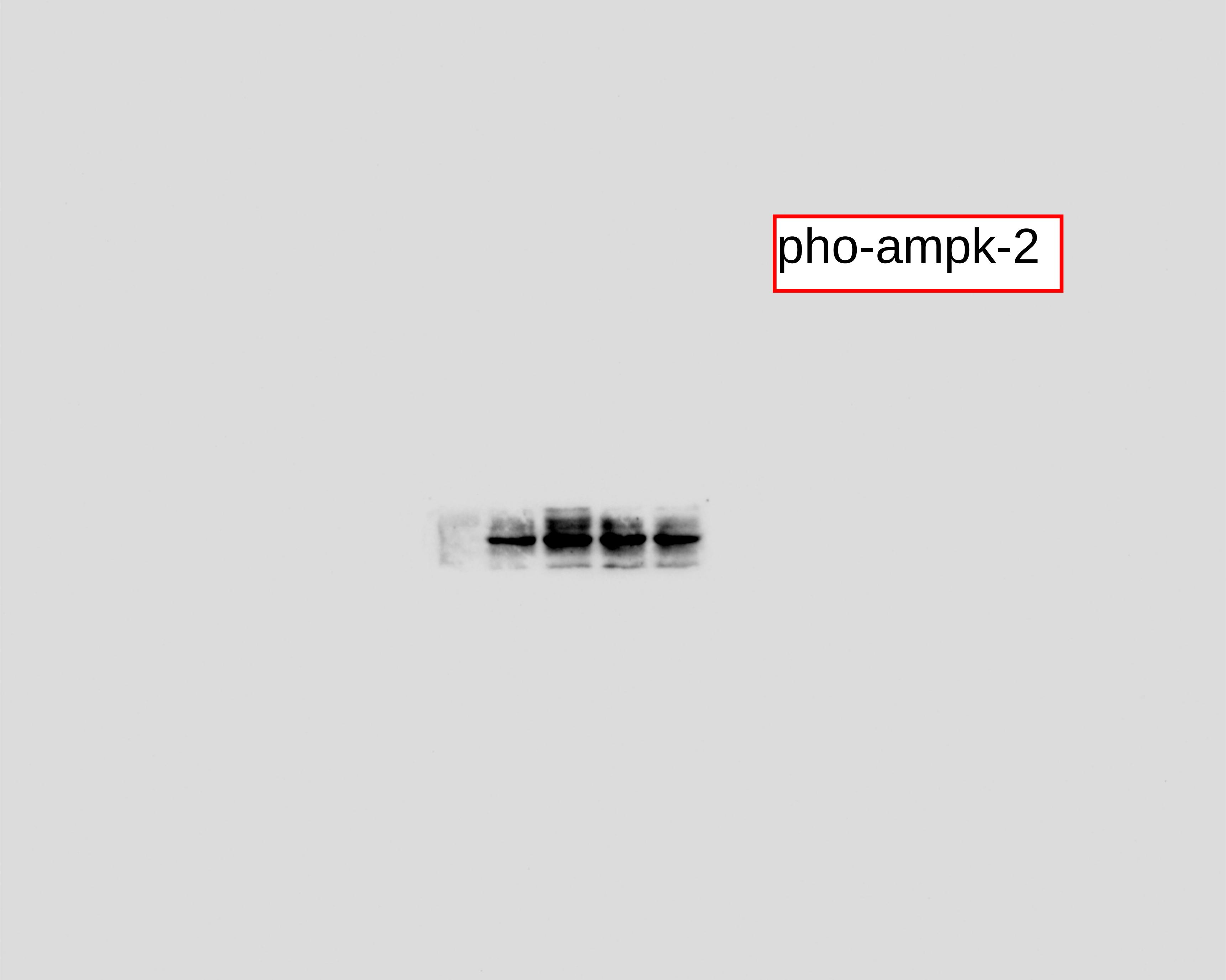
pho-ampk-2

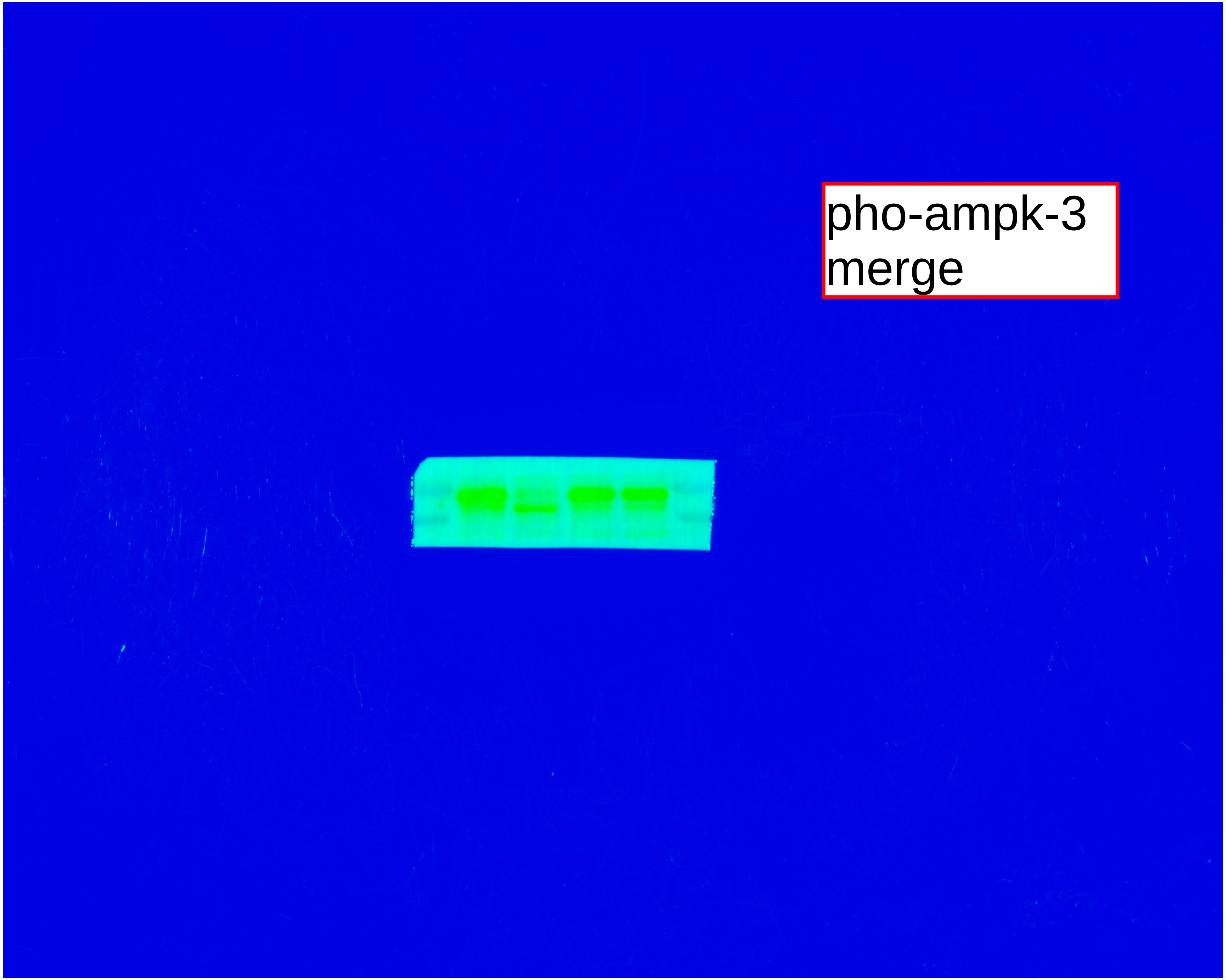
pho-ampk-3
merge

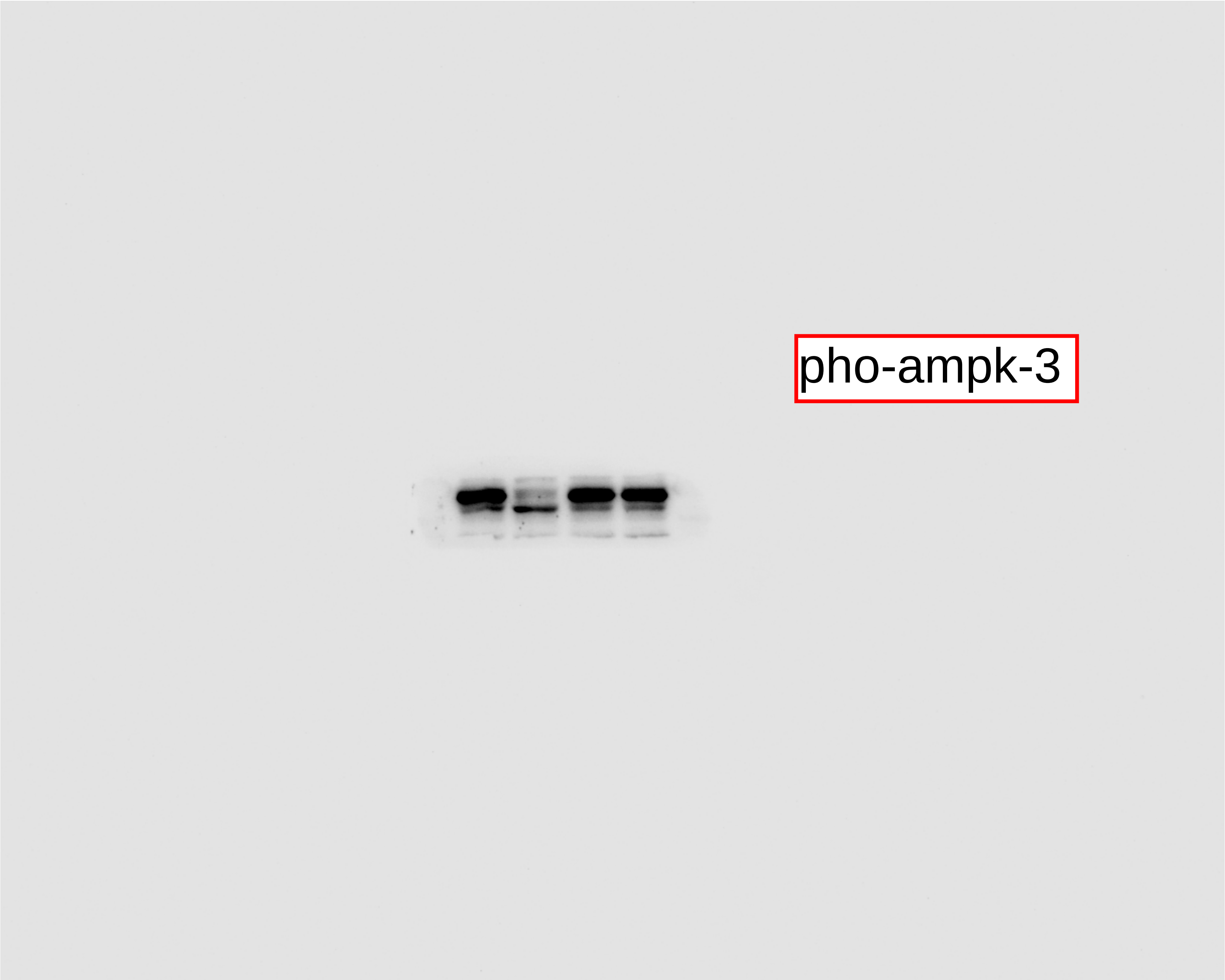
pho-ampk-3

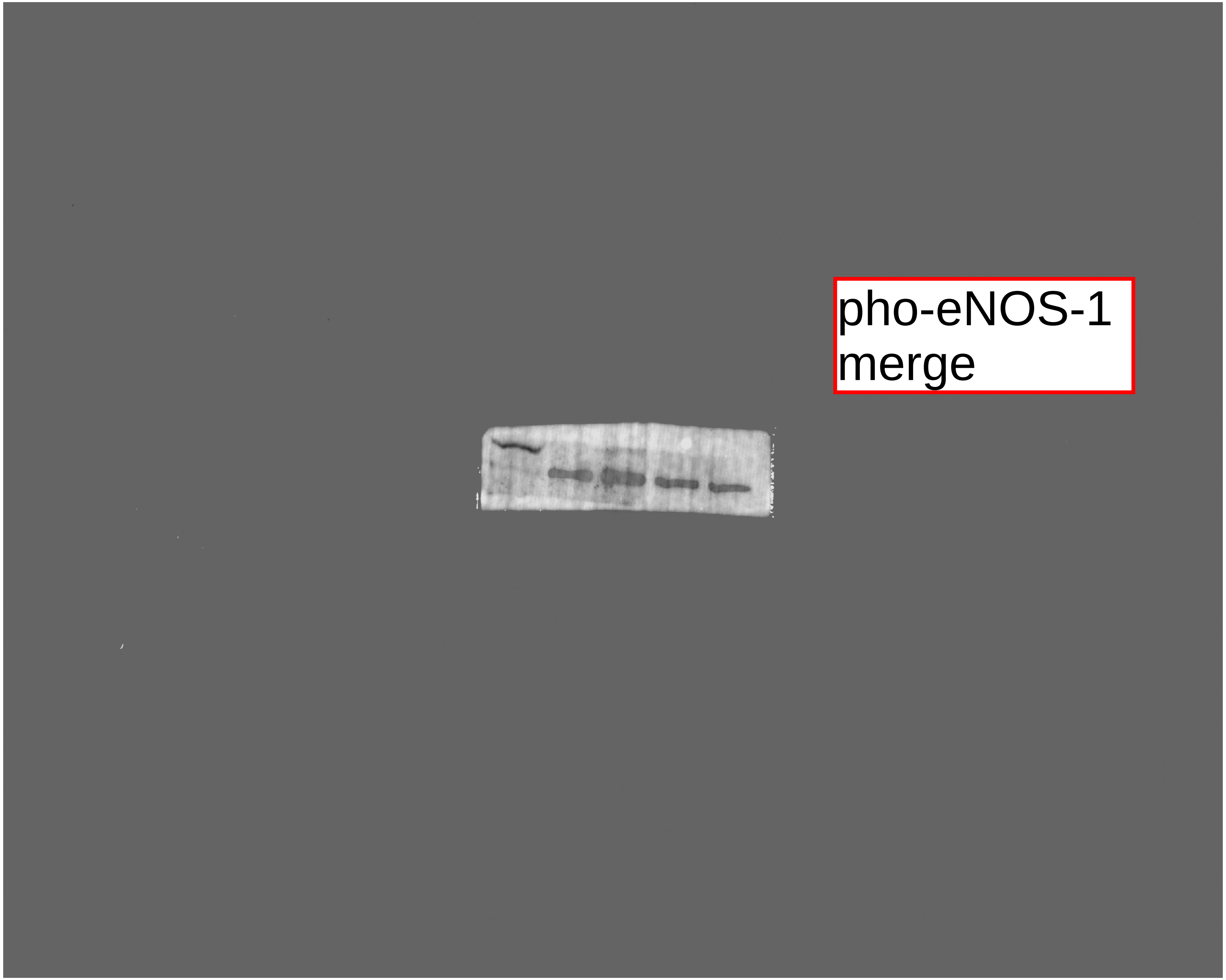
pho-eNOS-1
merge

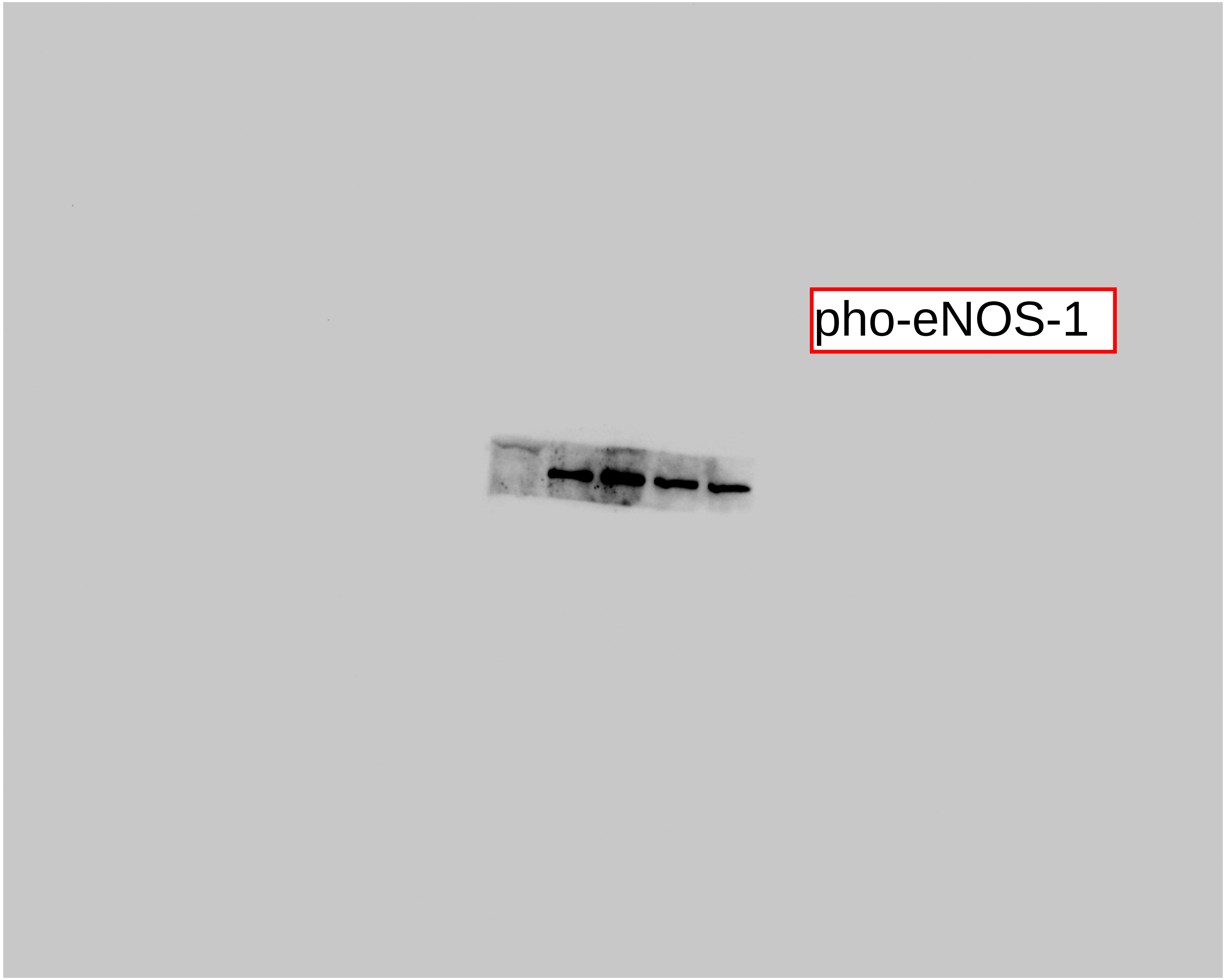
pho-eNOS-1

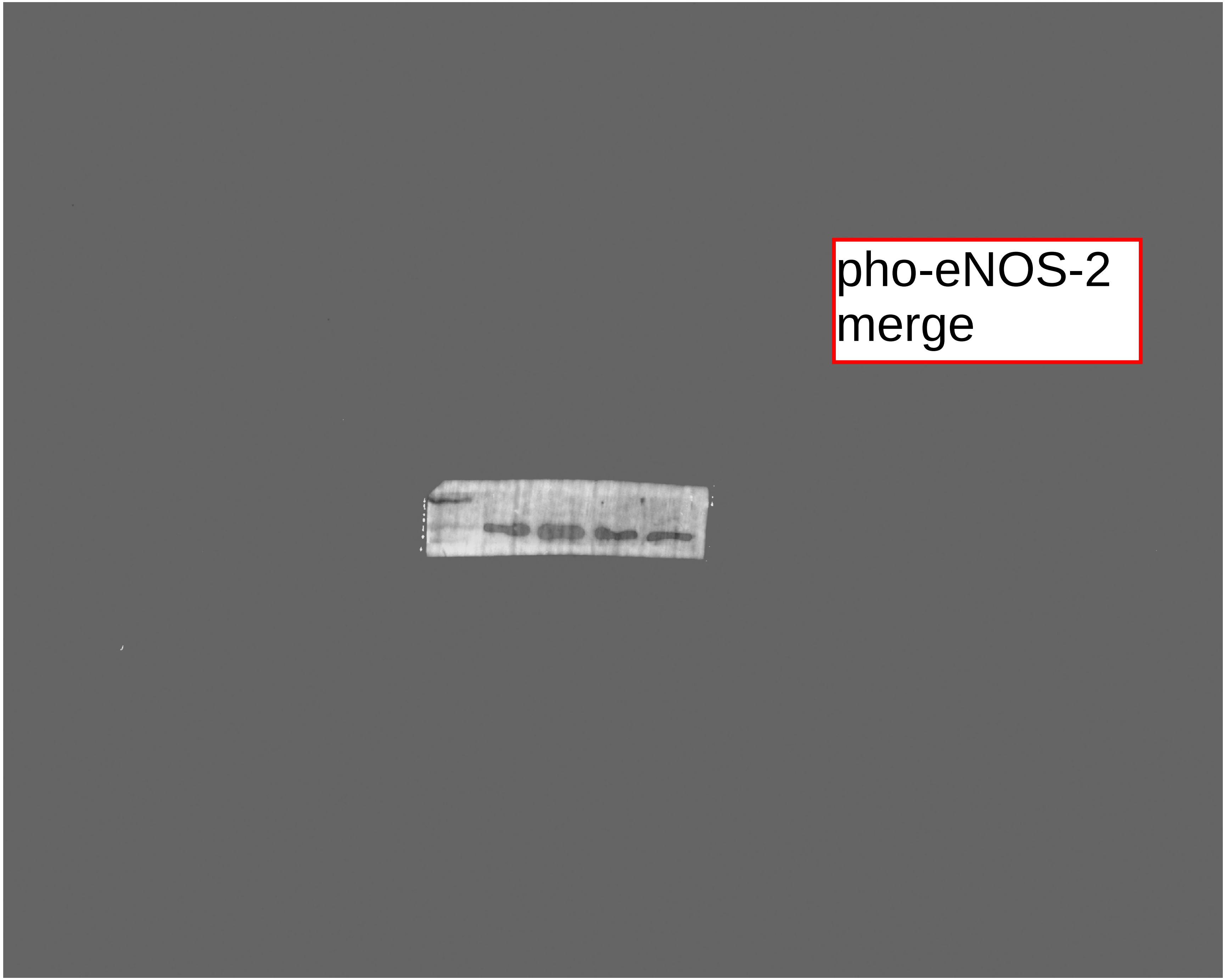

pho-eNOS-2
merge

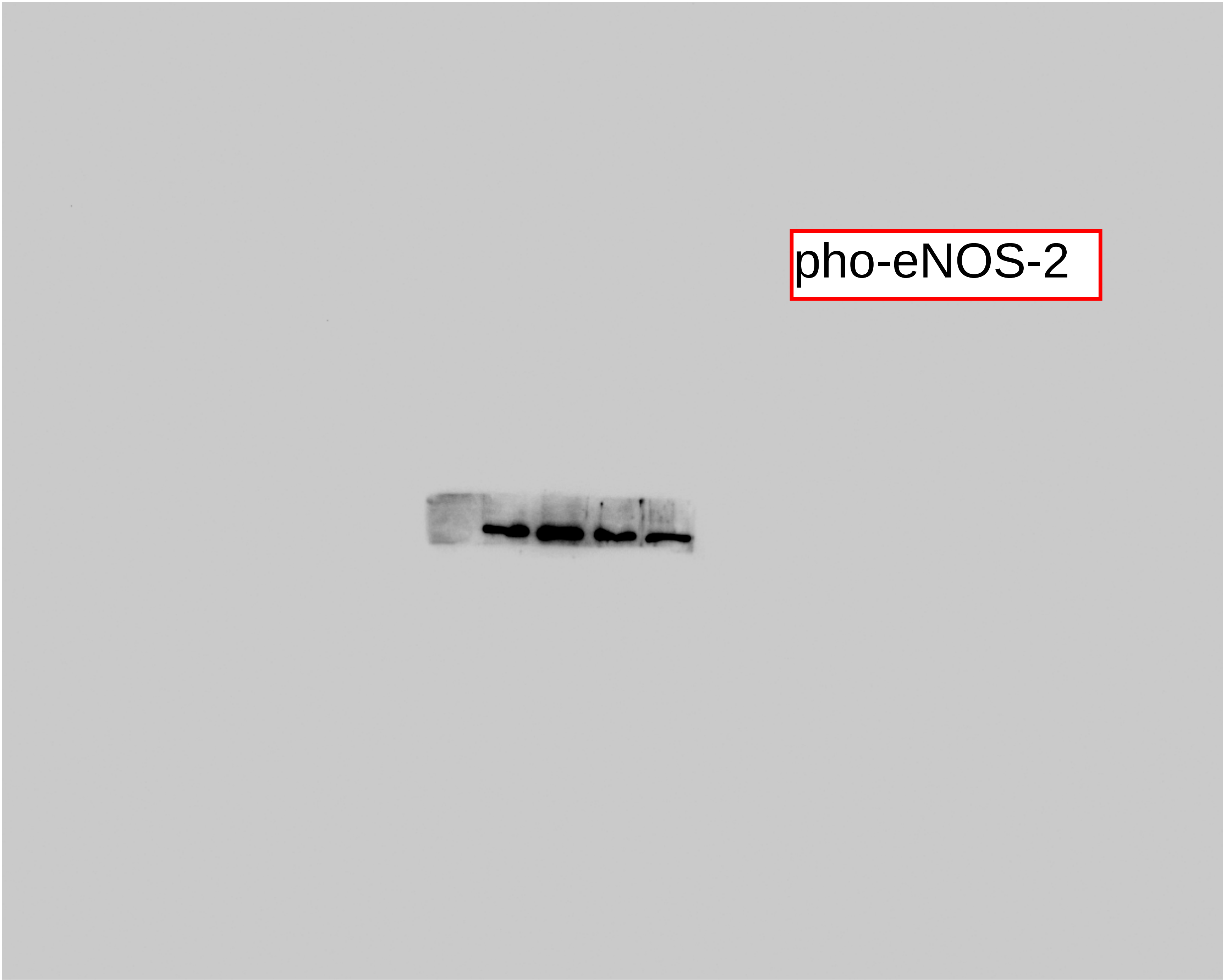
pho-eNOS-2

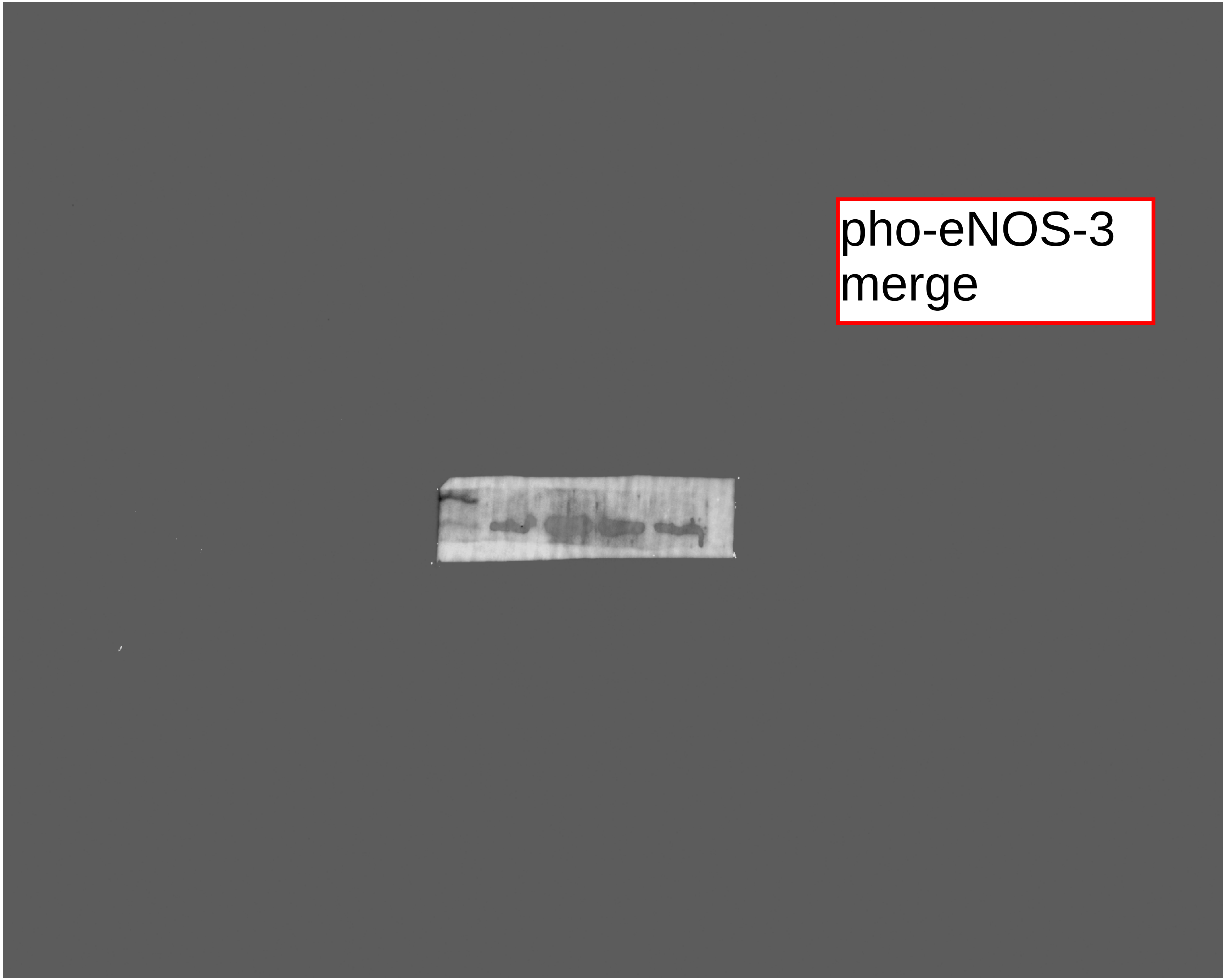
pho-eNOS-3
merge

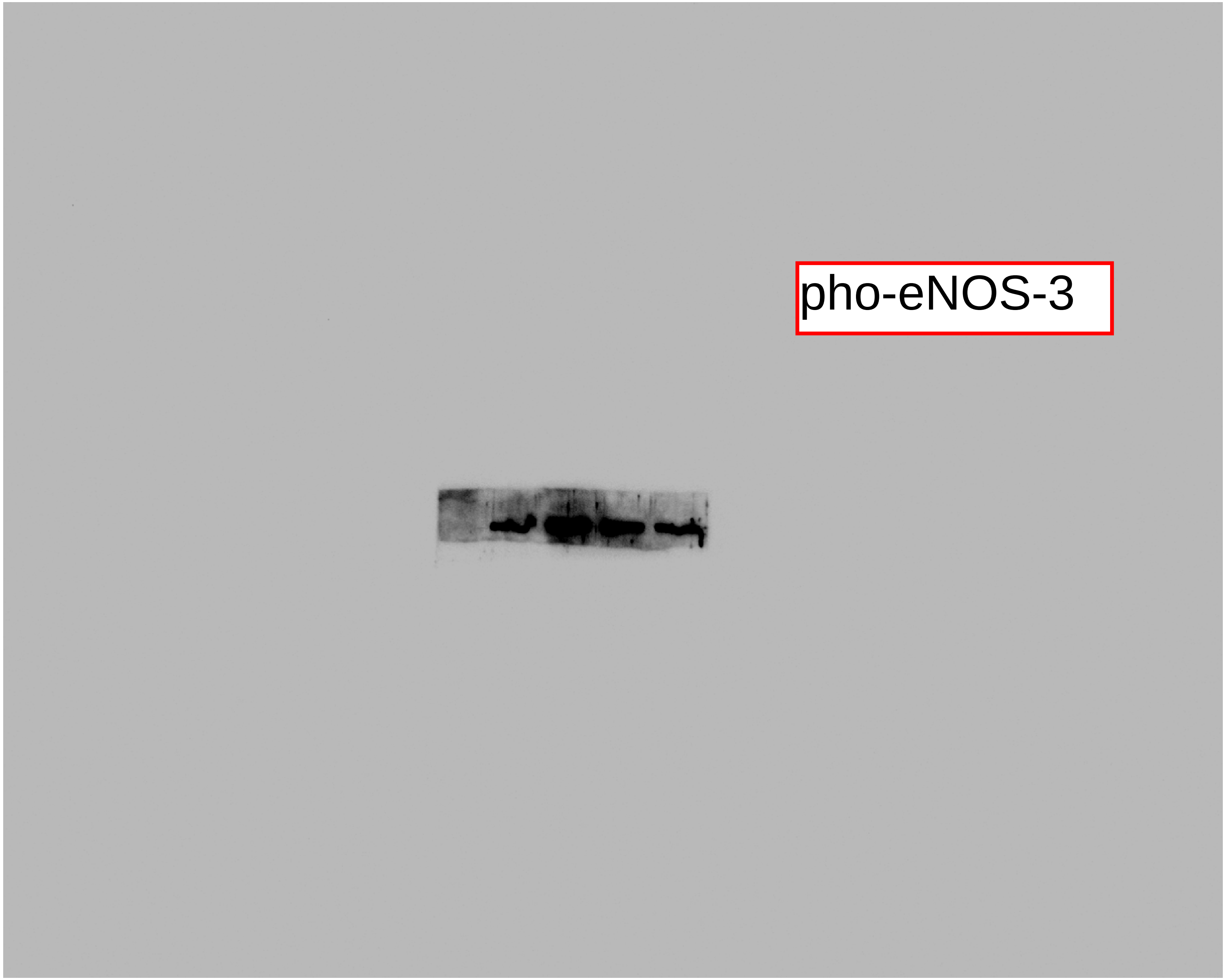
pho-eNOS-3

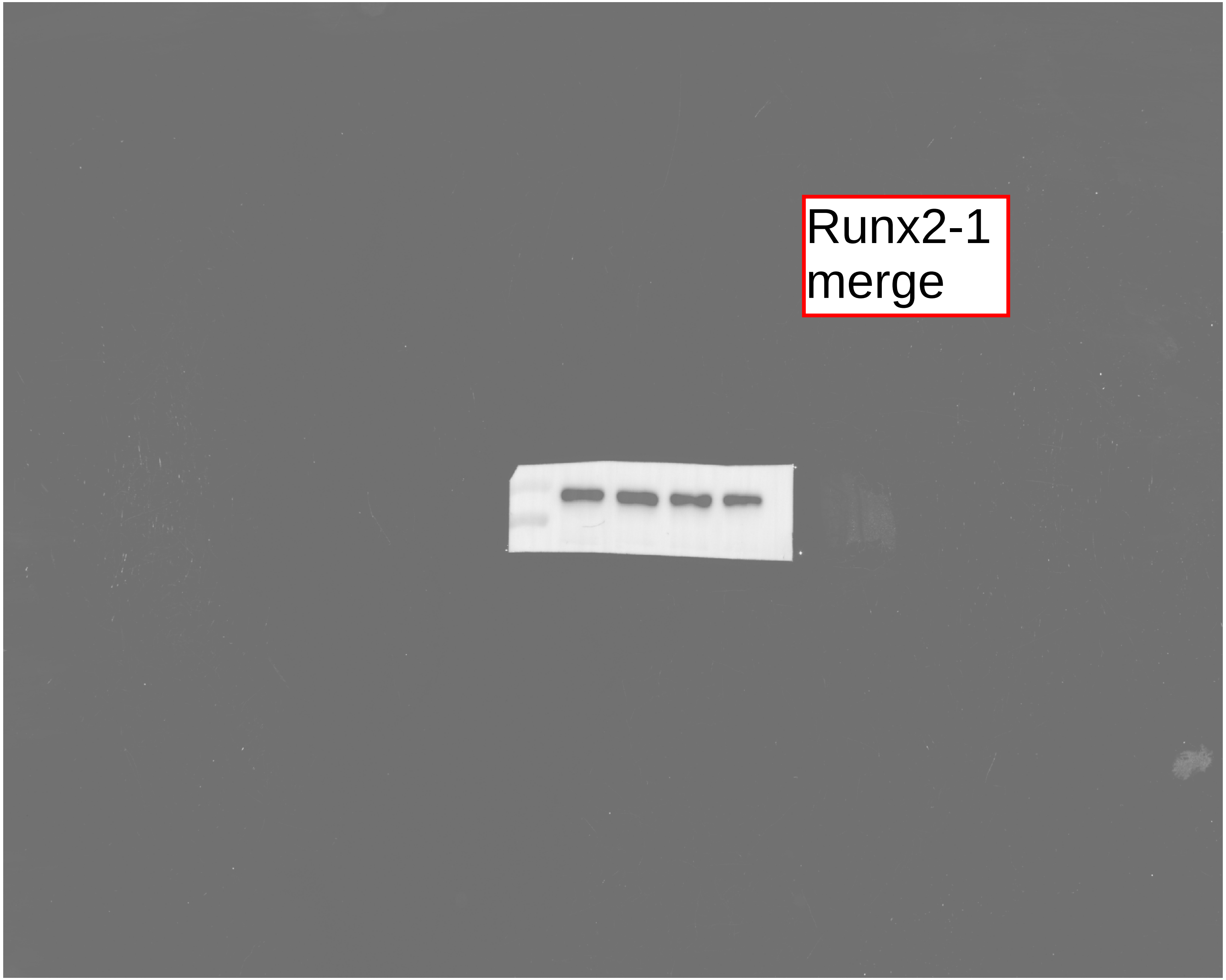
Runx2-1
merge

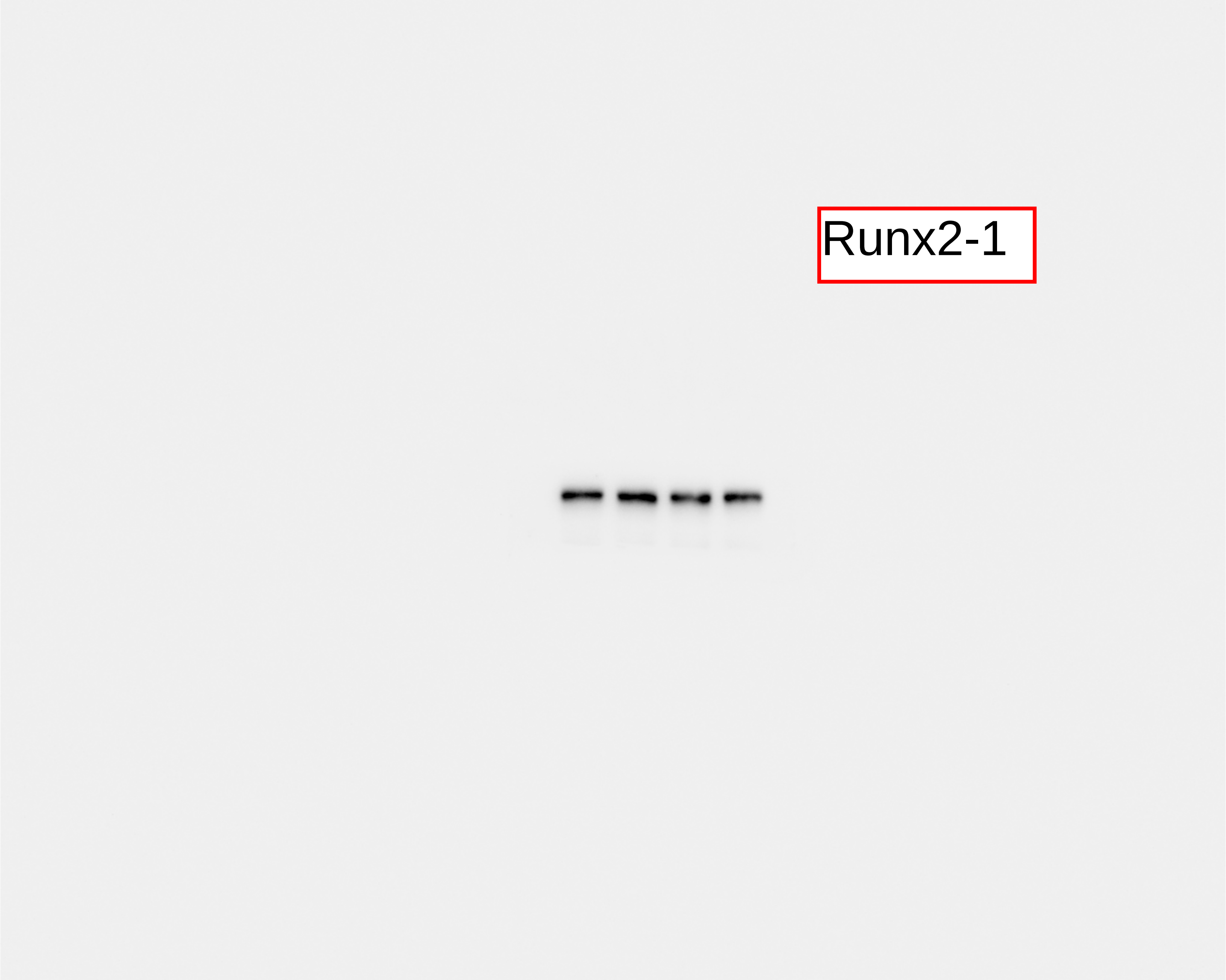
Runx2-1

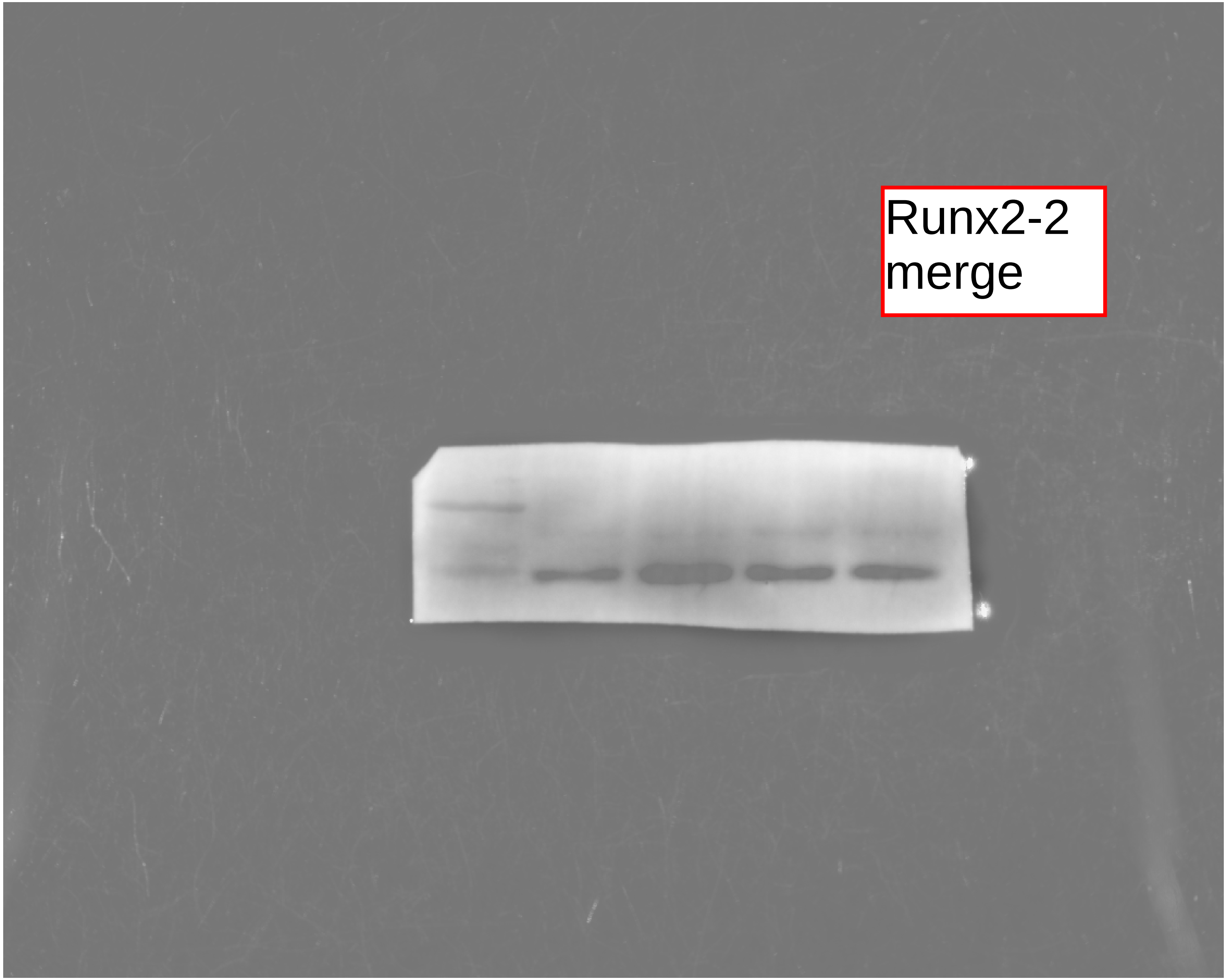
Runx2-2
merge

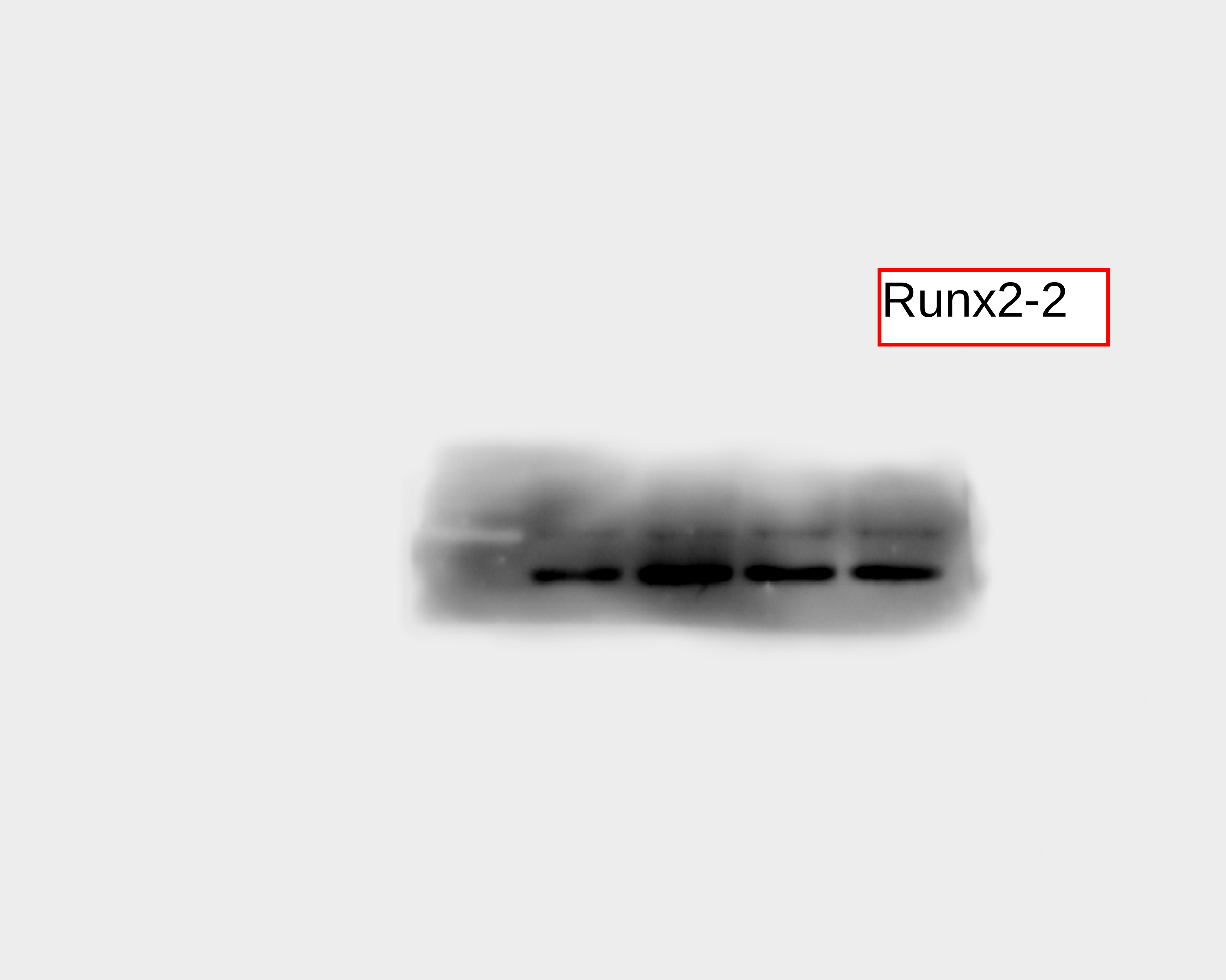
Runx2-2

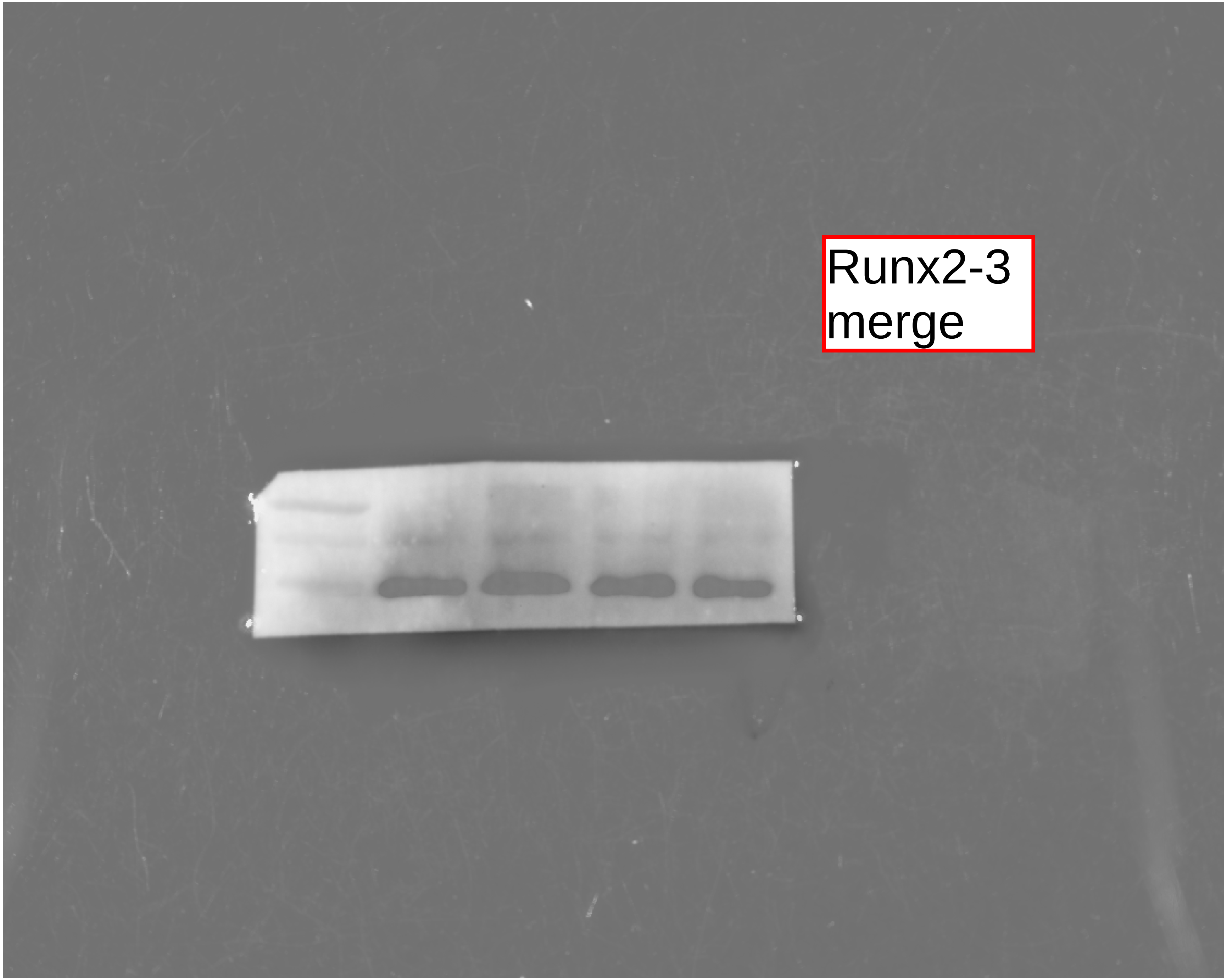
Runx2-3
merge

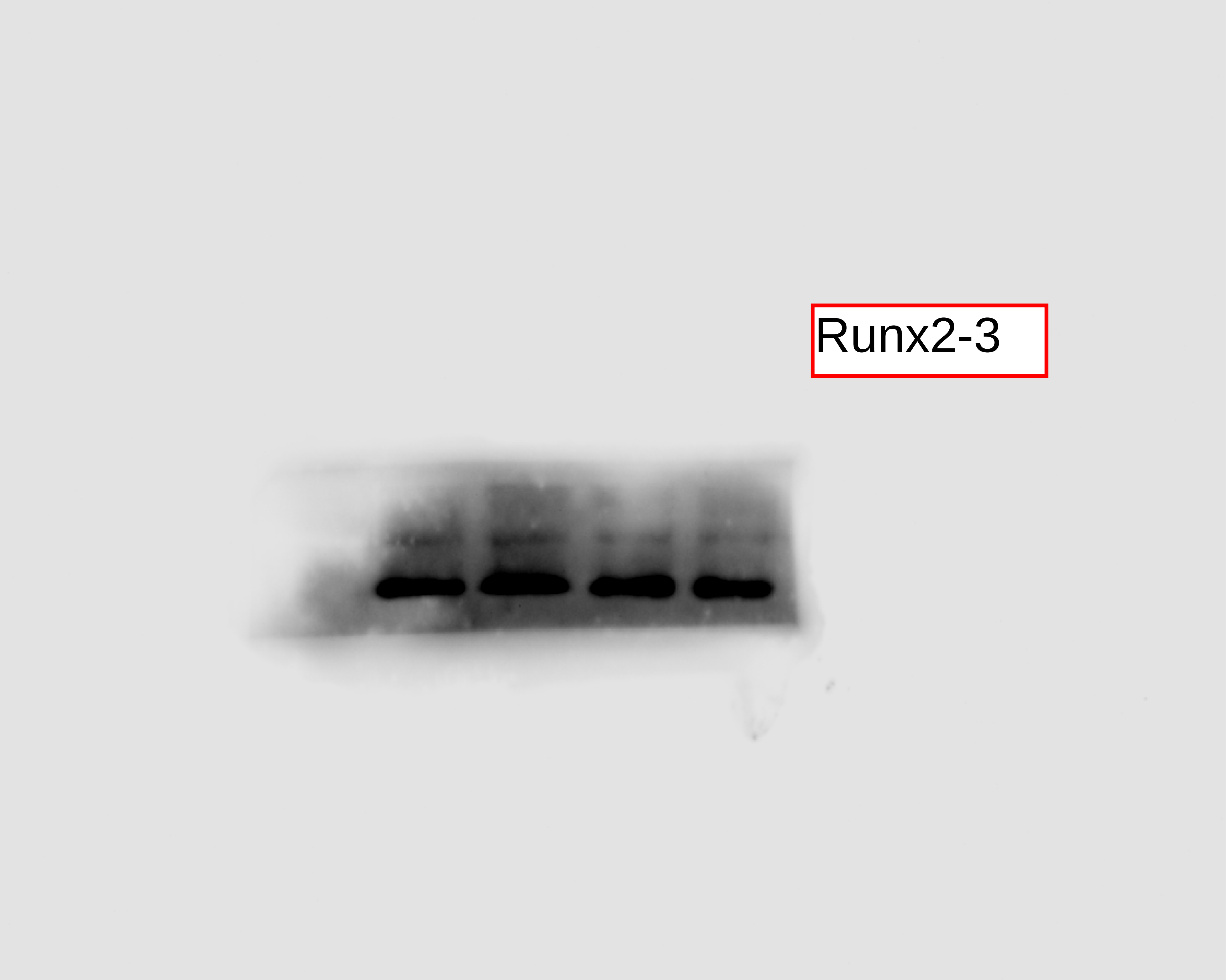
Runx2-3
